# Chemical Constituents with Anti-Proliferative Activity on Pulmonary Arterial Smooth Muscle Cells from the Roots of *Anthriscus sylvestris* (L.) Hoffm.

**DOI:** 10.3390/molecules29112547

**Published:** 2024-05-28

**Authors:** Yanling Liu, Yangang Cao, Yajuan Zheng, Ying Niu, Lan Chen, Xu Chen, Xinyi Ma, Xiangda Li, Xiaoke Zheng, Weisheng Feng

**Affiliations:** 1 School of Pharmacy, Henan University of Chinese Medicine, Zhengzhou 450046, China; liuyl9696@163.com (Y.L.); caoyangang1987@126.com (Y.C.); zyj18790251004@163.com (Y.Z.); 15516170034@163.com (Y.N.); m18638931582@163.com (L.C.); 18638197038@163.com (X.C.); maxinyi243028@163.com (X.M.); 15612430015@163.com (X.L.); zhengxk@hactcm.edu.cn (X.Z.); 2 The Engineering and Technology Center for Chinese Medicine Development of Henan Province China, Zhengzhou 450046, China; 3 Co-Construction Collaborative Innovation Center for Chinese Medicine and Respiratory Disease Diagnosis by Henan and Education Ministry of P. R. China, Zhengzhou 450046, China

**Keywords:** *Anthriscus sylvestris*, phenylpropanoid, phenol glycoside, anti-proliferative activity

## Abstract

A chemical investigation of *Anthriscus sylvestris* roots led to the isolation and characterization of two new nitrogen-containing phenylpropanoids (**1**–**2**) and two new phenol glycosides (**8**–**9**), along with fifteen known analogues. Structure elucidation was based on HRESIMS, 1D and 2D NMR spectroscopy, and electronic circular dichroism (ECD). In addition, compounds **3**, **6**, **9**–**10**, **12**, and **17** exhibited inhibitory effects against the abnormal proliferation of pulmonary arterial smooth muscle cells with IC50 values ranging from 10.7 ± 0.6 to 57.1 ± 1.1 μM.

## 1. Introduction

*Anthriscus sylvestris* L. Hoffm. (Umbelliferae), known as wild chervil, is a common perennial plant in Europe, North America, and Asia [1,2]. Its dried root has been used as an anti-pyretic, analgesic, diuretic, and anti-tussive agent in traditional Chinese medicine [3]. The major classes of phytochemicals of *A. sylvestris* include lignans, phenylpropanoids, and flavonoids [4,5]. Pharmacological investigations have demonstrated that this plant possesses certain biological properties such as anti-tumor, anti-proliferative, anti-asthmatic, and anti-inflammatory [6,7,8].

Pulmonary arterial hypertension (PAH) is a chronic, progressive disease of the pulmonary circulation characterized by vascular remodeling [9]. Pulmonary vascular remodeling involves a variety of cells, including vascular endothelial cells, pulmonary artery smooth muscle cells (PASMCs), and multiple inflammatory cells [10,11]. The previous study demonstrated that diarylpentanoids and phenylpropanoids isolated from the roots of *A. sylvestris* could inhibit the abnormal proliferation of PASMCs induced by hypoxia [12], which attracted our interest to search for more natural products with anti-proliferative effects from this plant. In this study, two new nitrogen-containing phenylpropanoids (**1**–**2**), and two new phenol glycosides (**8**–**9**), along with fifteen known analogues, were isolated from the roots of *A. sylvestris*, and their anti-proliferative effects against the abnormal proliferation of pulmonary arterial smooth muscle cells were evaluated.

## 2. Results and Discussion

### 2.1. Structure Characterization

**Figure 1:**
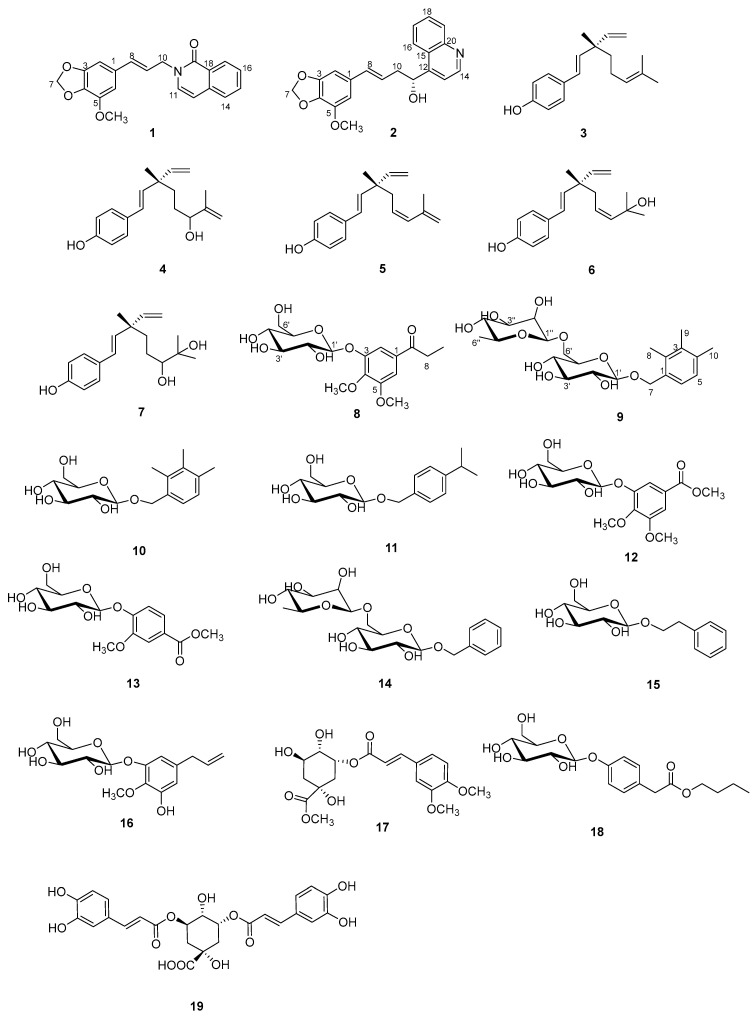
The chemical structures of compounds **1**–**19**.

The chemical investigations on the extract of the roots of *A. sylvestris* resulted in the characterization of compounds (**1**–**19**) (Figure 1).

**Figure 2:**
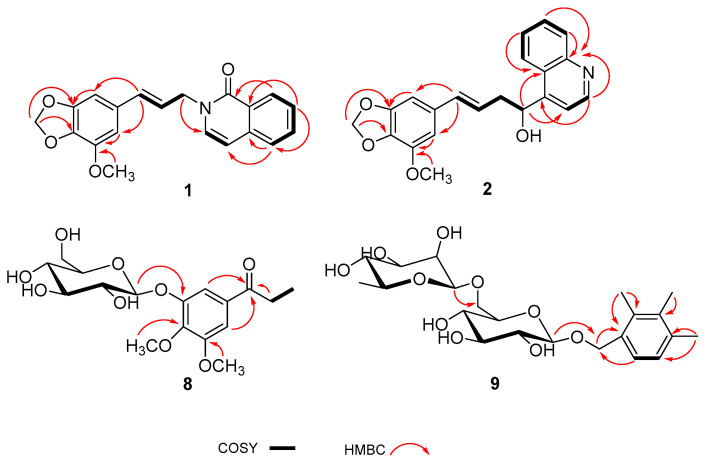
The key HMBC and ^1^H-^1^H COSY correlations of compounds **1**–**2** and **8**–**9**.

Compound **1**, a white amorphous powder, was assigned a molecular formula of C_20_H_17_NO_4_ due to the HRESIMS ion at *m*/*z* 336.1230 [M + Na]^+^ (calcd. 336.1232). The 1D NMR spectrum of **1** exhibited the signals of a carbonyl carbon [*δ*_C_ 180.3 (C-19)], a tetrasubstituted aromatic ring [*δ*_H_ 6.62 (1H, s, H-2), 6.56 (1H, s, H-6); *δ*_C_ 150.8 (C-3), 145.0 (C-5), 136.8 (C-4), 132.3 (C-1), 108.8 (C-6), 100.9 (C-2)], a disubstituted aromatic ring [*δ*_H_ 8.35 (1H, dd, *J* = 7.8, 1.3 Hz, H-15), 7.80 (2H, overlap, H-14,17), 7.47 (1H, t, *J* = 7.8 Hz, H-16); *δ*_C_ 141.6 (C-13), 133.9 (C-14), 127.8 (C-18), 127.1 (C-15), 125.4 (C-16), 118.2 (C-17)], two olefinic groups [*δ*_H_ 8.11 (1H, d, *J* = 7.6 Hz, H-11), 6.47 (1H, d, *J* = 15.9 Hz, H-8), 6.36 (1H, d, *J* = 7.6 Hz, H-12), 6.29 (1H, dt, *J* = 15.9, 5.8 Hz, H-9); *δ*_C_ 146.4 (C-11), 134.6 (C-8), 122.6 (C-9), 110.2 (C-12)], an oxygenated methylene group [*δ*_H_ 5.88 (2H, s, H-7); *δ*_C_ 102.7 (C-7)], a nitrogenated methylene group [*δ*_H_ 5.07 (2H, d, *J* = 5.8 Hz, H-10); *δ*_C_ 55.8 (C-10)], and a methoxy group [*δ*_H_ 3.82 (3H, s, OCH_3_-5); *δ*_C_ 57.2 (OCH_3_-5)]. The 1D NMR data of compound **1** were very similar to those of 2-[2-(1,3-benzodioxol-5-yl)ethyl]-6-methoxy-1(2H)-isoquinolinone [13]. Major discrepancies were concentrated on the absence of the methoxy group at C-16 and the presence of the methoxy group at C-5, and the Δ^8(9)^ double bond linked with a methylene carbon, which was confirmed by the ^1^H-^1^H COSY correlations of H-9 with H-8 and H-10 and the HMBC crosspeaks from H-8 to C-2 and C-6, from H-10 to C-11, and from the hydrogens of the methoxy group (*δ*_H_ 3.82) to C-5 (Figure 2). Based on these data, the structure of compound **1** was identified and named as anthriscusin O.

**Figure 3:**
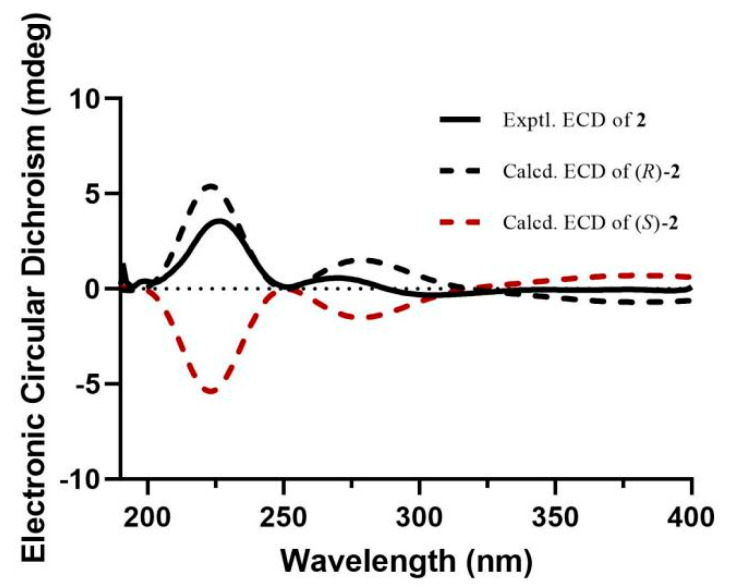
Experimental and calculated ECD spectra of compound **2**.

Compound **2** was obtained as a colorless solid. Its molecular formula, C_21_H_19_NO_4_, was established by the HRESIMS ion at *m*/*z* 372.1205 [M + Na]^+^ (calcd. for 372.1206). The ^1^H and ^13^C NMR data (Table 1) of **2** revealed the presence of a tetrasubstituted aromatic ring [*δ*_H_ 6.52 (1H, s, H-2), 6.47 (1H, s, H-6); *δ*_C_ 150.6 (C-3), 144.9 (C-5), 135.9 (C-4), 133.9 (C-1), 108.0 (C-6), 100.5 (C-2)], a quinoline unit [*δ*_H_ 8.82 (1H, d, *J* = 4.6 Hz, H-14), 8.20 (1H, d, *J* = 8.4 Hz, H-16), 8.05 (1H, d, *J* = 8.4 Hz, H-19), 7.78 (1H, t, *J* = 8.4 Hz, H-17), 7.70 (1H, d, *J* = 4.6 Hz, H-13), 7.64 (1H, t, *J* = 8.4 Hz, H-18); *δ*_C_ 153.3 (C-12), 151.0 (C-14), 148.7 (C-20), 130.6 (C-17), 129.9 (C-19), 127.9 (C-18), 127.0 (C-15), 124.7 (C-16), 119.1 (C-13)], an olefinic group [*δ*_H_ 6.27 (1H, d, *J* = 15.8 Hz, H-8), 6.17 (1H, dt, *J* = 15.8, 5.8 Hz, H-9); *δ*_C_ 133.6 (C-8), 125.6 (C-9)], an oxygenated methine group [*δ*_H_ 5.60 (1H, dd, *J* = 7.2, 5.8 Hz, H-11); *δ*_C_ 70.4 (C-11)], an oxygenated methylene group [*δ*_H_ 5.87 (2H, s, H-7); *δ*_C_ 102.5 (C-7)], a methylene group [*δ*_H_ 2.76 (1H, m, H-10a), 2.68 (1H, dd, *J* = 14.3, 7.2 Hz, H-10b); *δ*_C_ 43.0 (C-10)], and a methoxy group [*δ*_H_ 3.82 (3H, s, OCH_3_-5); *δ*_C_ 57.2 (OCH_3_-5)]. The ^1^H and ^13^C NMR data of **2** were similar to those of (2*E*)-3-(1,3-benzodioxol-5-yl)-2-propen-1-yl]benzenemethanol [14], except for the absence of the aromatic group and the presence of a quinoline unit at C-11 and an additional methoxy group at C-5, which was deduced by the HMBC correlations from H-11 to C-13 and C-15, and from the hydrogens of the methoxy group (*δ*_H_ 3.82) to C-5 (Figure 2). To define the absolute configuration, the ECD calculation was performed at the B3LYP/6-311G(d) level using the TDDFT method. Furthermore, the agreement of the Cotton effects of the calculated ECD spectrum of (*R*)-**2** with the experimental ECD spectrum of **2** allowed the absolute configuration of **2** to be assigned as (*R*) (Figure 3). Thus, the structure of **2** was elucidated as shown and named as anthriscusin P.

Compound **8** was acquired as a white amorphous powder with a molecular formula of C_17_H_24_O_9_, as determined by its HRESIMS and ^13^C NMR data. The ^1^H and ^13^C NMR data (Table 2) of **8** were similar to those of **12** [15], except for the replacement of the methoxy group at C-8 by the ethyl group in **8**, which was corroborated by the HMBC correlations (Figure S31, Supplementary Materials) of H-8 with *δ*_C_ 201.9 (C-7) and 8.7 (C-9). Additionally, the hexose moiety was determined as d-glucose through the chiral-phase HPLC analysis of a monosaccharide produced by the hydrolysis of compound **8** (Figure S63, Supplementary Materials). The anomeric proton (*δ*_H_ 4.95) of hexose moiety had a large coupling constant (*J* = 7.4 Hz), indicating a *β*-configuration. Thus, the structure of **8** was defined as shown and named as 1-(3-*β*-d-glucopyranosyloxy)-4,5-dimethoxyphenyl)propan-1-one.

Compound **9** was acquired as colorless crystals. According to the HRESIMS at *m*/*z* 481.2053 [M + Na]^+^, its molecular formula was inferred as C_22_H_34_O_10_. The ^1^H NMR spectrum showed signals for two aromatic protons [*δ*_H_ 7.06 (1H, d, *J* = 7.7 Hz, H-6), 6.94 (1H, d, *J* = 7.7 Hz, H-5)], an oxygenated methylene group [*δ*_H_ 4.89 (1H, overlap, H-7a), 4.58 (1H, d, *J* = 11.2 Hz, H-7b)], three methyl groups [*δ*_H_ 2.28 (3H, s, H-8), 2.25 (1H, s, H-10), 2.18 (1H, s, H-9)], and two anomeric protons [*δ*_H_ 4.79 (1H, d, *J* = 1.2 Hz, H-1′′), 4.25 (1H, d, *J* = 7.8 Hz, H-1′)]. The ^13^C NMR spectrum exhibited resonances for six aromatic carbons [*δ*_C_ 137.5 (C-4), 137.0 (C-2), 136.3 (C-3), 133.8 (C-1), 128.5 (C-6), 127.9 (C-5)], an oxygenated methylene carbon [*δ*_C_ 70.9 (C-7)], and three methyl groups [*δ*_C_ 20.9 (C-10), 15.7 (C-8, 9)]. Additionally, two anomeric carbons (*δ*_C_ 102.6 and 102.2), as well as ten carbon signals [*δ*_C_ 78.1 (C-3′), 76.9 (C-5′), 75.0 (C-2′), 74.0 (C-4′′), 72.4 (C-3′′), 72.2 (C-2′′), 71.4 (C-4′), 69.8 (C-6′), 68.1 (C-5′′), 18.1 (C-6′′)] indicated the occurrence of two sugar substituents. The hexose moieties were identified as d-glucose and l-rhamnose by chiral-phase HPLC analysis (Figure S64, Supplementary Materials). Moreover, the anomeric proton (*δ*_H_ 4.25) of d-glucose had a large coupling constant (*J* = 7.8 Hz), and the anomeric proton (*δ*_H_ 4.79) of l-rhamnose had a small coupling constant (*J* = 1.2 Hz), which suggested the configurations of the anomeric carbons of d-glucose and l-rhamnose were *β* and *α*, respectively. In the HMBC spectrum, the anomeric proton of the d-glucose unit showed a correlation with C-7, and the anomeric proton of the l-rhamnose unit was correlated with the C-6′ of d-glucose unit. These NMR data mentioned above were similar to those of compound **14 [16]**, except for the presence of three methyl groups. The three methyl groups were respectively located at C-2, C-3, and C-4, which was determined by the key HMBC correlations from the methyl protons at *δ*_H_ 2.28 to C-1, C2, and C-3, from the methyl protons at *δ*_H_ 2.18 to C-3 and C-4, and from the methyl protons at *δ*_H_ 2.25 to C-4 and C-5 (Figure 2). Based on these data, the structure of compound **9** was identified and named as 2,3,4-trimethylbenzylalcohol-*α*-l-rhamnopyranosyl-(6→1)-*β*-d-glucopyranoside.

Fifteen known compounds were identified as bakuchiol (**3**) [17], 1,3-hydroxybakuchiol (**4**) [17], 15-demetyl-12,13-dihydro-13-ketobakuchiol (**5**) [17], 3,2-hydroxybakuchiol (**6**) [17], 12,13-diolbakuchiol (**7**) [18], 2,3,4-trimethylbenzylalcohol-*O*-*β*-d-glucopyranoside acid (**10**) [19], p-cymen-7-yloxy-*β*-d-glucopyranoside (**11**) [20], methyl di-*O*-methyl-*O*-glucosylgallate (**12**) [15], methyl 4-(*β*-d-glucopyranosyloxy)-3-methoxybenzoate (**13**) [21], benzyl *α*-l-rhamnopyranosyl-(1→6)-*β*-d-glucopyranoside (**14**) [16], 2-phenylethyl-*β*-d-glucopyranoside (**15**) [22], 3,5-dihydroxyestragole 3-*O*-*β*-d-glucopyranoside (**16**) [23], 5-*O*-feruloylquinic acid methyl ester (**17**) [24], butyl(4-*β*-d-glucopyranosyloxy-phenyl)acetate (**18**) [25], and 3,5-dicaffeoylquinic acid (**19**) [26].

### 2.2. Biological Activity

**Figure 4:**
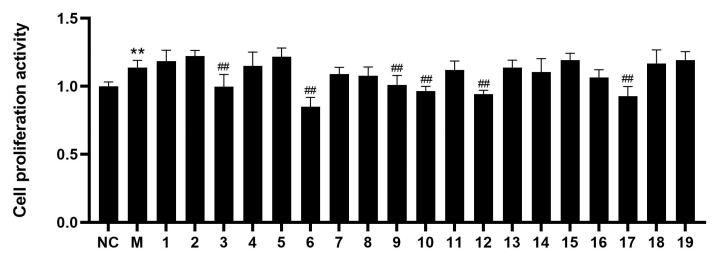
The inhibitory effects of compounds **1**–**19** were tested in hypoxia-stimulated PASMCs by MTT assay. (Compared with NC group, ** *p* < 0.01; Compared with M group, ^##^ *p* < 0.01.)

The isolates **1**–**19** were screened for their inhibitory effects on PASMCs’ abnormal proliferation induced by hypoxia in vitro. As shown in Figure 4, compared with the normal (NC) group, the proliferation rate of PASMCs in the model (M) group was significantly increased (*p* < 0.01). Compared with the M group, the proliferation rates of the compounds **3**, **6**, **9**–**10**, **12**, and **17** groups were significantly decreased (*p* < 0.01), which indicated that compounds **3**, **6**, **9**–**10**, **12**, and **17** can significantly inhibit the abnormal proliferation of PASMCs at 5 μM. Then, these cells were treated with the compounds (**3**, **6**, **9**–**10**, **12**, and **17**) with different concentrations (1, 2.5, 5, 10, 20, 50, and 100 μM). Compounds **3**, **6**, **9**–**10**, **12**, and **17** suppressed the abnormal proliferation of PASMCs, with IC_50_ values of 56.3 ± 0.5, 10.7 ± 0.65, 57.1 ± 1.1, 44.0 ± 0.75, 41.5 ± 0.87, and 35.3 ± 0.42 μM, respectively. Notably, the comparison of compounds **8** and **12** showed that the methoxy fragment connected to C-8 may be responsible for the inhibition. Additionally, compounds **9** and **10** exhibited an inhibitory effect in contrast to compounds **14** and **15**, which might be attributed to the presence of a 2,3,4-trimethylphenyl group.

Pharmacological studies have shown that deoxypodophyllotoxin isolated from *A. sylvestris* possesses antitumor, antibacterial, and antiviral activities [7,8,27,28], which has led to the pharmacological effects of *A. sylvestris* being mainly focused on antitumor effects, with less research and attention paid to other pharmacological effects. In this study, the inhibitory effects of compounds isolated from *A. sylvestris* on the hypoxia-induced cell proliferation of PASMCs were investigated for the first time. The preliminary results of this experiment have an important potential value for future development and research on *A. sylvestris*.

## 3. Experimental

### 3.1. General Experimental Procedures

MS spectra were obtained using a Bruker maXis HD mass spectrometer (Bruker, Bremen, Germany). Optical rotations were measured on a Rudolph AP-Ⅳ polarimeter (Rudolph, Hackettstown, NJ, USA). IR spectra were recorded on a Thermo Nicolet IS 10 spectrometer (Thermo, Waltham, MA, USA). UV spectra were recorded on a ThermoEVO 300 spectrometer (Thermo, Waltham, MA, USA). NMR spectra were acquired using a Bruker Avance III 500 spectrometer (Bruker, Bremen, Germany). ECD spectra were recorded on an Applied Photophysics Chirascan qCD spectropolarimeter (AppliedPhotophysics, Leatherhead, Surrey, UK). Semipreparative HPLC separations were conducted on a Saipuruisi LC 52 HPLC system with a UV/vis 50 detector (Saipuruisi, Beijing, China) and a YMC Pack ODS A column (20 × 250 mm, 5 μm; YMC, Kyoto, Japan). Monosaccharide elucidation was conducted on a Waters 2695 separation module equipped with an evaporative light-scattering detector (ELSD) (Waters, Milford, MA, USA) using a CHIRALPAK AD-H column (4.6 × 250 mm) (Daicel Chiral Technologies Co., Ltd., Shanghai, China). Column chromatography was performed using the Toyopearl HW-40C (TOSOH Corp, Tokyo, Japan), Sephadex LH-20 (40–70 mm, Amersham Pharmacia Biotech AB, Uppsala, Sweden), and silica gel (200–300 mesh, Marine Chemical Industry, Qingdao, China). The chemical reagents were supplied by the Tianjin Fuyu Fine Chemical Industry, Tianjin, China.

### 3.2. Plant Material

The dried roots of *A. sylvestris* were collected in November 2021 from Leshan city, Sichuan province, China, and identified by Professor Chengming Dong of Henan University of Chinese Medicine. A voucher specimen (No. 20211116A) was deposited at the Department of Natural Medicinal Chemistry, Henan University of Chinese Medicine, Zhengzhou, China.

### 3.3. Extraction and Isolation

The chopped dried roots (48.0 kg) were extracted with 70% aqueous acetone (smashed tissue extraction). The extract (15 kg) was suspended in water and sequentially partitioned with petroleum ether, EtOAc, and *n*-BuOH for fifteen times, respectively.

The EtOAc fraction (100.0 g) was separated by silica gel column chromatography (CC) eluted with a petroleum ether-EtOAc (100:0−0:100) gradient system and an EtOAc-CH_3_OH (100:0−0:100) gradient system and yielded seven subfractions (E1−E7). Subfraction E6 (6.5 g) was chromatographed using silica gel CC eluted with a CH_2_Cl_2_-CH_3_OH (200:1–1:1) gradient system to give seven subfractions (E6-1–E6-7). Subfraction E6-2 (510.0 mg) was separated by the Toyopearl HW-40C CC (CH_3_OH-H_2_O 70:30) to obtain three subfractions (E6-2-1–E6-2-3). Subfraction E6-2-2 (200.0 mg) was purified by semipreparative HPLC (CH_3_OH-H_2_O 84:16) to produce compounds **4** (5.3 mg), **6** (3.1 mg), and **7** (8.0 mg). Subfraction E6-3 (400.0 mg) was separated by the Sephadex LH-20 (CH_3_OH-H_2_O 70:30) to obtain three subfractions (E6-3-1–E6-3-3). Subfraction E6-3-1 (49.0 mg) was purified by semipreparative HPLC (CH_3_OH-H_2_O 89:11) to yield compounds **3** (3.9 mg) and **5** (3.5 mg). Subfraction E6-3-3 (88.0 mg) was purified by semipreparative HPLC (CH_3_OH-H_2_O 70:30) to yield compounds **1** (7.5 mg) and **2** (5.3 mg). Subfraction E6-4 (280.0 mg) was purified by semipreparative HPLC (CH_3_OH-H_2_O 52:48) to yield compounds **10** (20.4 mg) and **16** (5.0 mg). Subfraction E6-5 (1.9 g) was subjected to silica gel CC eluted with a petroleum EtOAc-CH_3_OH gradient system (80:1–1:1) to give five subfractions (E-6-5-1–E6-5-5). Subfraction E6-5-3 (550.0 mg) was separated by Toyopearl HW-40C CC (CH_3_OH-H_2_O 50:50) to obtain four subfractions (E6-5-3-1–E6-5-3-4). Then subfraction E6-5-3-2 (89.9 mg) was purified by semipreparative HPLC (CH_3_OH-H_2_O 60:40) to produce compounds **8** (5.0 mg) and **12** (10.3 mg). Subfraction E6-5-4 (100.0 mg) was purified by semipreparative HPLC (CH_3_OH-H_2_O 52:48) to yield compound **9** (13.0 mg).

The *n*-BuOH fraction (160.0 g) was separated by Diaion HP-20 eluted with a petroleum ethanol-H_2_O (0:100−95:5) gradient to produce seven subfractions (N1−N7). Subfraction N3 (10.5 g) was subjected to ODS gel CC (CH_3_OH-H_2_O 10:90−50:50) to obtain five subfractions (N3-1–N3-5). Subfraction N3-3 (2.0 g) was chromatographed with the Sephadex LH-20 CC (MeOH-H_2_O 30:70) to obtain five subfractions (N3-3-1−N3-3-5). Then, subfraction N3-3-2 (480.9 mg) was subjected to silica gel CC eluted with a petroleum EtOAc-CH_3_OH gradient system (50:1–1:1) to give three subfractions (N3-3-2-1–N3-3-2-3). Subfraction N3-3-2-2 (90.0 mg) was purified by semipreparative HPLC (CH_3_OH-H_2_O 55:45) to yield compounds **13** (3.1 mg), **15** (2.5 mg), and **17** (5.1 mg). Subfraction N3-3-2-3 (120.0 mg) was purified by semipreparative HPLC (CH_3_OH-H_2_O 52:48) to yield compounds **11** (3.5 mg) and **18** (4.8 mg). Subfraction N3-4 (1.5 g) was subjected to the Toyopearl HW-40C CC (CH_3_OH-H_2_O 80:20) to obtain four subfractions (N3-4-1–N3-4-4). Then subfraction N3-4-3 (530.9 mg) was subjected to silica gel CC eluted with a petroleum EtOAc-CH_3_OH gradient system (50:1–1:1) to give four subfractions (N3-4-3-1–N3-4-3-4). Subfraction N3-4-3-3 (100.0 mg) was purified by semipreparative HPLC (CH_3_OH-H_2_O 55:45) to yield compounds **14** (7.1 mg) and **19** (16.0 mg).

Anthriscusin O (**1**): white amorphous powder; UV (CH_3_OH) *λ*_max_: 213, 280, 322, and 335 nm; IR (iTR) ν_max_: 2922, 1621, and 1025 cm^−1^; HRESIMS *m*/*z* 336.1230 [M + Na]^+^ (calcd. for C_20_H_17_NO_4_Na, 336.1232); ^1^H and ^13^C NMR data, see Table 1.

Anthriscusin P (**2**): white amorphous powder; ${\left[\alpha \right]}_{D}^{25}$ +17.1 (c 0.04, CH_3_OH); UV (CH_3_OH) *λ*_max_: 224 and 278 nm; IR (iTR) ν_max_: 3327, 2931, 1625, 1509, and 1135 cm^−1^; ECD (CH_3_OH) *λ*_max_ (Δ*ε*): 226 (+3.5) and 267 (+0.5); HRESIMS *m*/*z* 372.1205 [M + Na]^+^ (calcd. for C_21_H_19_NO_4_Na, 372.1206); ^1^H and ^13^C NMR data, see Table 1.

1-(3-*β*-d-glucopyranosyloxy)-4,5-dimethoxyphenyl)propan-1-one (**8**): white amorphous powder; UV (CH_3_OH) *λ*_max_: 202 nm; IR (iTR) ν_max_: 3354, 2926, 1454, and 1041 cm^−1^; HRESIMS *m*/*z* 395.1318 [M + Na]^+^ (calcd. for C_17_H_24_O_9_Na, 395.1312); ^1^H and ^13^C NMR data, see Table 2.

2,3,4-trimethylbenzylalcohol-*α*-l-rhamnopyranosyl-(6→1)-*β*-d-glucopyranoside (**9**): white amorphous powder; UV (CH_3_OH) *λ*_max_: 217 and 272 nm; IR (iTR) ν_max_: 3396, 2938, 1675, 1589, 1419, and 1075 cm^−1^; HRESIMS *m*/*z* 481.2053 [M + Na]^+^ (calcd. for C_22_H_34_O_10_Na, 481.2044); ^1^H and ^13^C NMR data, see Table 2.

### 3.4. Computational Analysis

The conformations of **2** were analyzed by GMMX software (6.0) using the MMFF94 force field. The conformers were optimized with density functional theory (DFT) at the B3LYP/6-31G using the Gaussian 2016 package. The ECD calculations of conformers with Boltzmann distributions over 1% were further calculated by the TDDFT method at the B3LYP/6-311G (d) level in CH_3_OH. The ECD spectra were simulated by the SpecDis 1.71 software [29].

### 3.5. MTT Assay

Briefly, the PASMCs were seeded in 96-well plates at 2 × 10^4^ cells/well at 37 °C in an atmosphere of 5% CO_2_. The cells were divided into the normal group (NC), model group (M), and treatment groups (isolated compounds). Then, the MTT assay was performed as previously described [12]. All data were analyzed by SPSS software version 26.0 (IBM, New York, NY, USA) and presented as the mean ± standard deviation. Concentration-response analysis was performed to determine the compound concentrations required to inhibit the growth of cells by 50% (IC_50_) using GraphPad Prism 8.02 software.

## 4. Conclusions

Four new compounds (**1**–**2**, **8**, and **9**), together with fifteen known analogs were isolated from the roots of *A. sylvestris*. All compounds were isolated from the plant for the first time, which greatly enriches the chemical content of this plant. In preliminary in vitro bioassays, the results showed that **3**, **6**, **9**–**10**, **12**, and **17** exhibited anti-proliferation effects on hypoxia-induced PASMCs’ cell proliferation, suggesting that they may further act as potential lead molecules for the development of therapeutic agents for PAH. Then, we will discover more bioactive compounds from *A. sylvestris* and carry out further research on the mechanism with potential compounds for the treatment of PAH.

## Figures and Tables

**Table 1:** ^1^H (500 MHz) and ^13^C (125 MHz) NMR spectroscopic and HMBC data of compounds **1**–**2** in CD_3_OD.

Position	1			2		
	*δ*_H_ (*J* in Hz)	*δ*_C_ (Type)	HMBC (H→C)	*δ*_H_ (*J* in Hz)	*δ*_C_ (Type)	HMBC (H→C)
1		132.3 (C)			133.9 (C)	
2	6.62 (brs)	100.9 (CH)	3, 4, 6, 8	6.52 (brs)	100.5 (CH)	1, 3, 4, 6, 8
3		150.8 (C)			150.6 (C)	
4		136.8 (C)			135.9 (C)	
5		145.0 (C)			144.9 (C)	
6	6.56 (brs)	108.8 (CH)	2, 4, 5, 8	6.47 (brs)	108.0 (CH)	1, 2, 4, 5, 8
7	5.88 (s)	102.7 (CH_2_)	3, 4	5.87 (s)	102.5 (CH_2_)	3, 4
8	6.47 (d, 15.9)	134.6 (CH)	2, 9, 10	6.27 (d, 15.8)	133.6 (CH)	1, 2, 6, 9, 10
9	6.29 (dt, 15.9, 5.8)	122.6 (CH)	1, 10	6.17 (dt, 15.8, 5.8)	125.6 (CH)	1, 10, 11
10	5.07 (d, 5.8)	55.8 (CH_2_)	8, 9, 11	2.76 (m) 2.68 (dd, 14.3, 7.2)	43.0 (CH_2_)	8, 9, 11, 12
11	8.11 (d, 7.6)	146.4 (CH)	10, 12, 13, 19	5.60 (dd, 7.2, 5.8)	70.4 (CH)	9, 12, 13
12	6.36 (d, 7.6)	110.2 (CH)	11, 18		153.3 (C)	
13		141.6 (C)		7.70 (d, 4.6)	119.1 (CH)	11, 14, 15
14	7.80 (overlap)	133.9 (CH)		8.82 (d, 4.6)	151.0 (CH)	12, 13, 15, 20
15	8.35 (dd, 7.8, 1.3)	127.1 (CH)	13, 14		127.0 (C)	
16	7.47 (t, 7.8)	125.4 (CH)	15, 17, 18	8.20 (d, 8.4)	124.7 (CH)	12, 15, 17, 20
17	7.80 (overlap)	118.2 (CH)		7.78 (t, 8.4)	130.6 (CH)	17, 20
18		127.8 (C)		7.64 (t, 8.4)	127.9 (CH)	16, 17
19		180.3 (C)		8.05 (d, 8.4)	129.9 (CH)	18, 20
20					148.7 (C)	
5-OCH_3_	3.82 (s)	57.2 (CH_3_)	5	3.82 (s)	57.2 (CH_3_)	5

**Table 2:** ^1^H (500 MHz) and ^13^C (125 MHz) NMR spectroscopic and HMBC data of compounds **8**–**9** in CD_3_OD.

Position	8			9		
	*δ*_H_ (*J* in Hz)	*δ*_C_ (Type)	HMBC (H→C)	*δ*_H_ (*J* in Hz)	*δ*_C_ (Type)	HMBC (H→C)
1		133.7 (C)			133.8 (C)	
2	7.52 (brs)	111.9 (CH)	1, 3, 4, 6, 7		137.0 (C)	
3		152.1 (C)			136.3 (C)	
4		144.5 (C)			137.5 (C)	
5		154.7 (C)		6.94 (d, 7.7)	127.9 (CH)	1, 10
6	7.33 (brs)	107.3 (CH)	1, 2, 4, 5, 7	7.06 (d, 7.7)	128.5 (CH)	4, 7
7		201.9 (C)		4.89 (overlap) 4.58 (d, 11.2)	70.9 (CH_2_)	1, 2, 6, 1′
8	3.02 (d, 7.2)	32.5 (CH_2_)	7, 9	2.28 (s)	15.7 (CH_3_)	1, 2, 3
9	1.15 (t, 7.1)	8.7 (CH_3_)	7, 8	2.18 (s)	15.7 (CH_3_)	3, 4
10	3.89 (s)	61.6 (CH_3_)	4, 5	2.25 (s)	20.9 (CH_3_)	4, 5
11	3.88 (s)	56.7 (CH_3_)	4, 5			
1′	4.95 (d, 7.4)	103.0 (CH)	3, 3′	4.25 (d, 7.8)	102.6 (CH)	7, 5′
2′	3.50 (m)	74.9 (CH)	3′, 4′	3.18 (m)	75.0 (CH)	1′
3′	3.46 (m)	78.5 (CH)	4′	3.31 (m)	78.1 (CH)	4′
4′	3.33 (m)	71.4 (CH)	3′, 5′	3.29 (m)	71.8 (CH)	3′, 5′
5′	3.47 (m)	78.1 (CH)	4′	3.35 (m)	76.9 (CH)	4′
6′	3.91 (overlap) 3.67 (m)	62.5 (CH_2_)	4′, 5′	4.01 (dd, 11.2, 1.6) 3.62 (dd, 11.2, 6.3)	69.8 (CH_2_)	4′, 1′′
1′′				4.79 (d, 1.2)	102.2 (CH)	6′, 5′′
2′′				3.88 (dd, 3.3, 1.2)	72.2 (CH)	3′′
3′′				3.70 (m)	72.4 (CH)	2′′, 5′′
4′′				3.40 (m)	74.0 (CH)	6′′
5′′				3.70 (m)	68.1 (CH)	3′′
6′′				1.28 (d, 6.2)	18.1 (CH_3_)	3′′, 5′′

## Data Availability

The data presented in this study are available in the Supplementary Materials.

## Supplementary Materials

The following supporting information can be downloaded at: https://www.mdpi.com/article/10.3390/molecules29112547/s1, Figures S1–S16: 1D/2D NMR, HR-ESIMS, IR, and UV spectra of compounds **1**–**2**, Figures S17–S26: 1D NMR spectra of compounds **3**–**7**; Figures S27–S42: 1D/2D NMR, HR-ESIMS, IR, and UV spectra of compounds **8**–**9**; Figures S43–S61: 1D NMR spectra of compounds **10**–**19**; Figures S62–S64: Chiral-HPLC profile from acid hydrolysis of compounds **8** and **9** compared to authentic standard.
