# Supplementary material for: Chemical Constituents with Anti-Proliferative Activity on Pulmonary Arterial Smooth Muscle Cells from the Roots of Anthriscus sylvestris (L.) Hoffm
Source: Molecules. 2024 May 28;29(11):2547. doi: 10.3390/molecules29112547 (PMC11173479; doi:10.3390/molecules29112547)
Supplement: Supplementary file 1 [file molecules-29-02547-s001.zip › molecules-3039249-supplementary.pdf]

Chemical Constituents with Anti-Proliferative Activity on Pulmonary Arterial Smooth Muscle Cells from the Roots of *Anthriscus sylvestris* (L.) Hoffm.

Yanling Liu [1,2], Yangang Cao [1,2], Yajuan Zheng [1,2], Ying Niu [1,2], Lan Chen [1,2], Xu Chen [1,2], Xinyi Ma [1,2], Xiangda Li [1,2], Xiaoke Zheng [1,2,3] and Weisheng Feng [1,2,3,*]

1 School of Pharmacy, Henan University of Chinese Medicine, Zhengzhou 450046, China; liuyl9696@163.com (Y.L.); caoyangang1987@126.com (Y.C.); zyj18790251004@163.com (Y.Z.); 15516170034@163.com (Y.N.); m18638931582@163.com (L.C.); 18638197038@163.com (X.C.); maxinyi243028@163.com (X.M.); 15612430015@163.com (X.L.); zhengxk@hactcm.edu.cn (X.Z.)

2 The Engineering and Technology Center for Chinese Medicine Development of Henan Province China, Zhengzhou 450046, China

3 Co-construction Collaborative Innovation Center for Chinese Medicine and Respiratory Disease Diagnosis by Henan and Education Ministry of P. R. China, Zhengzhou 450046, China

* Correspondence: fwsh@hactcm.edu.cn

Contents

Figure S1. ^{1}H NMR spectrum (500MHz, CD_3OD) of **1** 4
Figure S2. ^{13}C NMR spectrum (125MHz, CD_3OD) of **1** 4
Figure S3. ^{1}H-^{1}H COSY spectrum of **1** 5
Figure S4. HSQC spectrum of **1** 5
Figure S5. HMBC spectrum of **1** 6
Figure S6. HR-ESI-MS spectrum of compound **1** 6
Figure S7. UV spectrum of **1** 7
Figure S8. IR spectrum of **1** 8
Figure S9. ^{1}H NMR spectrum (500MHz, CD_3OD) of **2** 8
Figure S10. ^{13}C NMR spectrum (125MHz, CD_3OD) of **2** 9
Figure S11. ^{1}H-^{1}H COSY spectrum of **2** 9
Figure S12. HSQC spectrum of **2** 10
Figure S13. HMBC spectrum of **2** 10
Figure S14. HR-ESI-MS spectrum of compound **2** 11
Figure S15. UV spectrum of **2** 12
Figure S16. IR spectrum of **2** 13
Figure S17. ^{1}H NMR spectrum (500MHz, CD_3OD) of **3** 13
Figure S18. ^{13}C NMR spectrum (125MHz, CD_3OD) of **3** 14
Figure S19. ^{1}H NMR spectrum (500MHz, CD_3OD) of **4** 14
Figure S20. ^{13}C NMR spectrum (125MHz, CD_3OD) of **4** 15
Figure S21. ^{1}H NMR spectrum (500MHz, CD_3OD) of **5** 15
Figure S22. ^{13}C NMR spectrum (125MHz, CD_3OD) of **5** 16
Figure S23. ^{1}H NMR spectrum (500MHz, CD_3OD) of **6** 16
Figure S24. ^{13}C NMR spectrum (125MHz, CD_3OD) of **6** 17
Figure S25. ^{1}H NMR spectrum (500MHz, CD_3OD) of **7** 17

Figure S26. ^{13}C NMR spectrum (125MHz, CD_3OD) of **7** 18
Figure S27. ^{1}H NMR spectrum (500MHz, CD_3OD) of **8** 18
Figure S28. ^{13}C NMR spectrum (125MHz, CD_3OD) of **8** 19
Figure S29. ^{1}H-^{1}H COSY spectrum of **8** 19
Figure S30. HSQC spectrum of **8** 20
Figure S31. HMBC spectrum of **8** 20
Figure S32. HR-ESI-MS spectrum of compound **8** 21
Figure S33. UV spectrum of **8** 22
Figure S34. IR spectrum of **8** 23
Figure S35. ^{1}H NMR spectrum (500MHz, CD_3OD) of **9** 23
Figure S36. ^{13}C NMR spectrum (125MHz, CD_3OD) of **9** 24
Figure S37. ^{1}H-^{1}H COSY spectrum of **9** 24
Figure S38. HSQC spectrum of **9** 25
Figure S39. HMBC spectrum of **9** 25
Figure S40. HR-ESI-MS spectrum of compound **9** 26
Figure S41. UV spectrum of **9** 27
Figure S42. IR spectrum of **9** 28
Figure S43. ^{1}H NMR spectrum (500MHz, CD_3OD) of **10** 28
Figure S44. ^{13}C NMR spectrum (125MHz, CD_3OD) of **10** 29
Figure S45. ^{1}H NMR spectrum (500MHz, CD_3OD) of **11** 29
Figure S46. ^{13}C NMR spectrum (125MHz, CD_3OD) of **11** 30
Figure S47. ^{1}H NMR spectrum (500MHz, CD_3OD) of **12** 30
Figure S48. ^{13}C NMR spectrum (125MHz, CD_3OD) of **12** 31
Figure S49. ^{1}H NMR spectrum (500MHz, CD_3OD) of **13** 31
Figure S50. ^{13}C NMR spectrum (125MHz, CD_3OD) of **13** 32
Figure S51. ^{1}H NMR spectrum (500MHz, CD_3OD) of **14** 32
Figure S52. ^{13}C NMR spectrum (125MHz, CD_3OD) of **14** 33
Figure S53. ^{1}H NMR spectrum (500MHz, CD_3OD) of **15** 33
Figure S54. ^{13}C NMR spectrum (125MHz, CD_3OD) of **15** 34
Figure S55. ^{1}H NMR spectrum (500MHz, CD_3OD) of **16** 34
Figure S56. ^{13}C NMR spectrum (125MHz, CD_3OD) of **16** 35
Figure S57. ^{1}H NMR spectrum (500MHz, CD_3OD) of **17** 35
Figure S58. ^{13}C NMR spectrum (125MHz, CD_3OD) of **17** 36
Figure S59. ^{1}H NMR spectrum (500MHz, CD_3OD) of **18** 36
Figure S60. ^{13}C NMR spectrum (125MHz, CD_3OD) of **18** 37
Figure S61. ^{1}H NMR spectrum (500MHz, CD_3OD) of **19** 37
Figure S62. ^{13}C NMR spectrum (125MHz, CD_3OD) of **19** 38
Figure S63. Chiral-HPLC profile from acid hydrolysis of **8** compared to authentic standard 38
Figure S64. Chiral-HPLC profile from acid hydrolysis of **9** compared to authentic standard 39

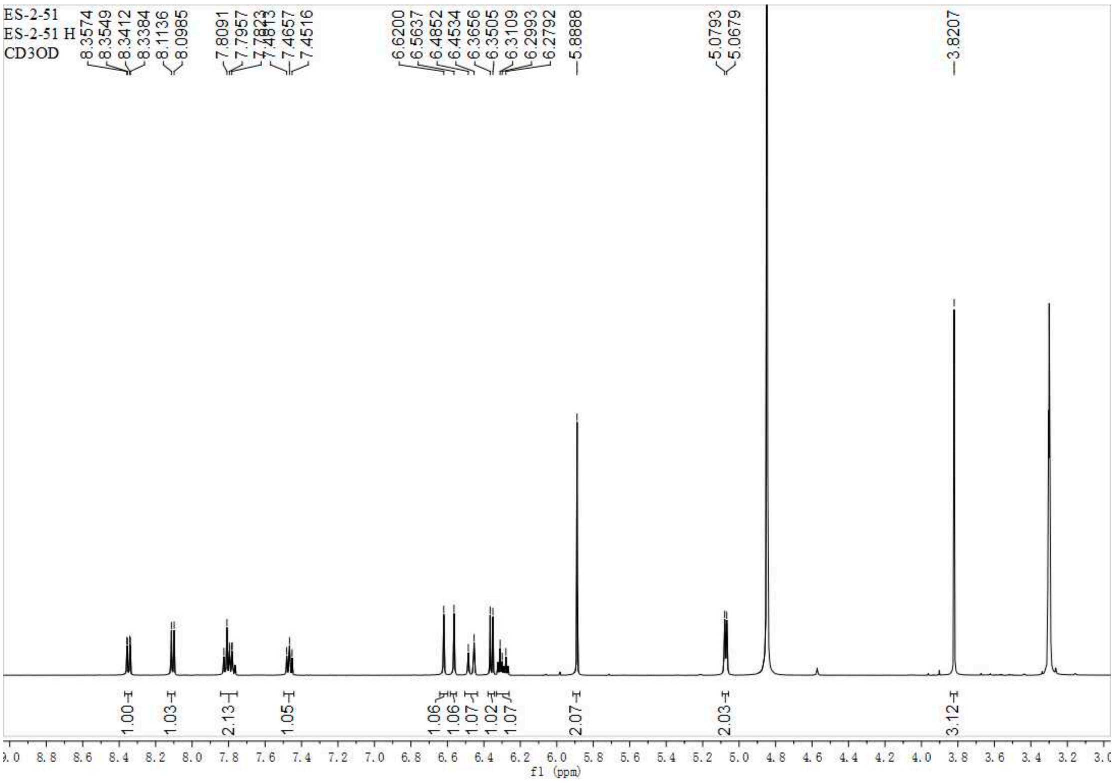

Figure S1. ^{1}H NMR spectrum (500MHz, CD_3OD) of **1**

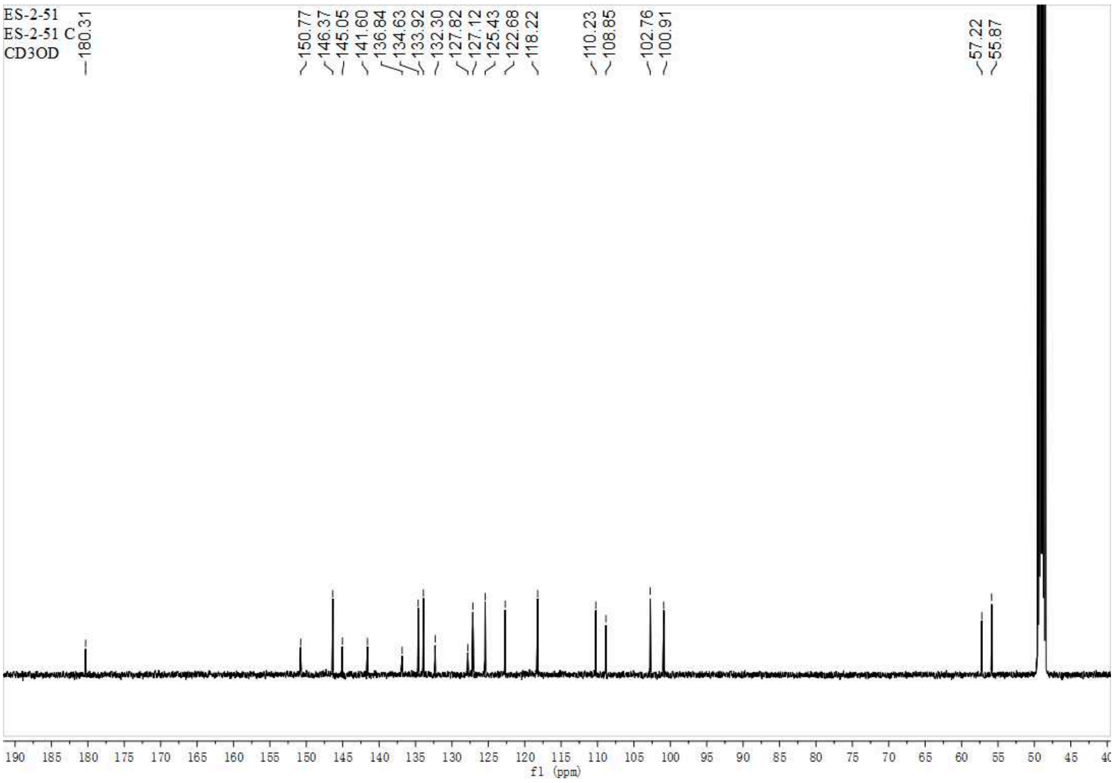

Figure S2. ^{13}C NMR spectrum (125MHz, CD_3OD) of **1**

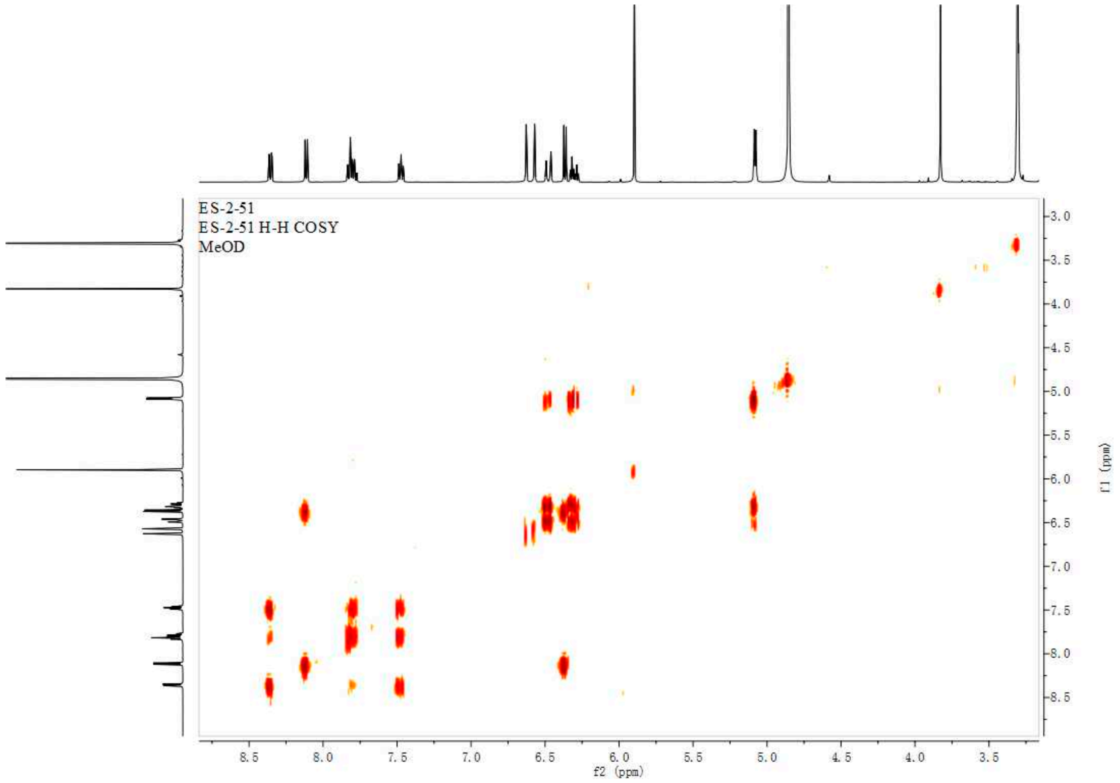

Figure S3. ^{1}H-^{1}H COSY spectrum of **1**

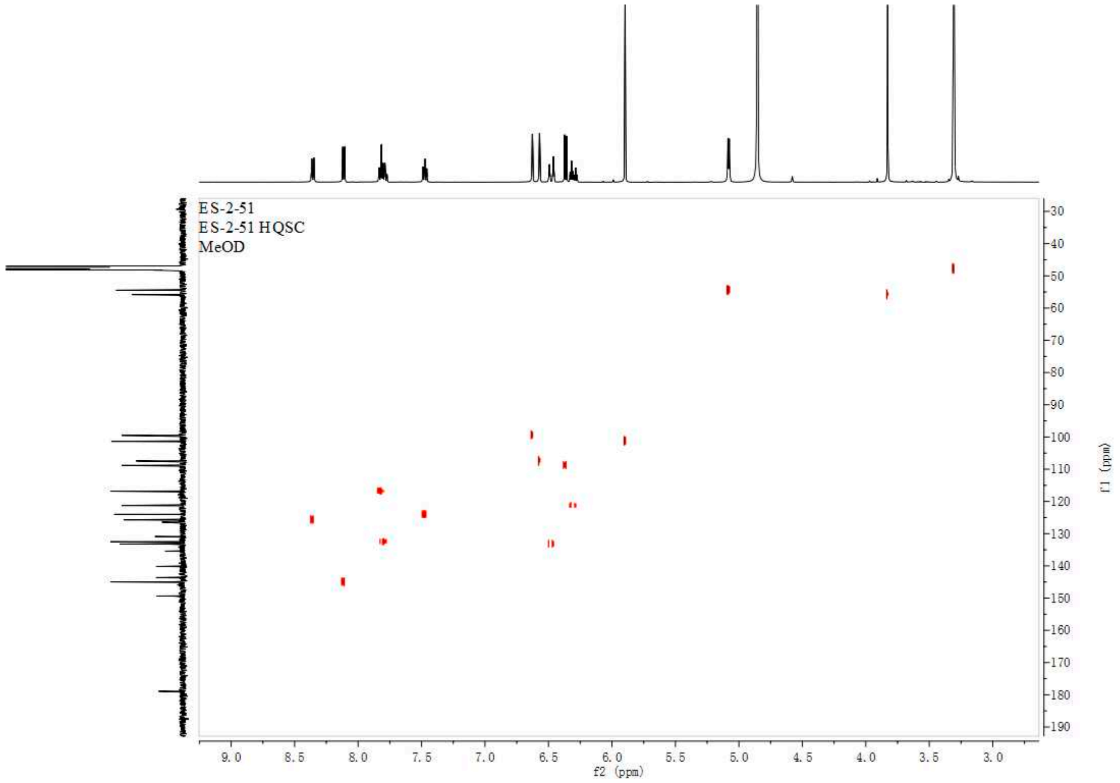

Figure S4. HSQC spectrum of **1**

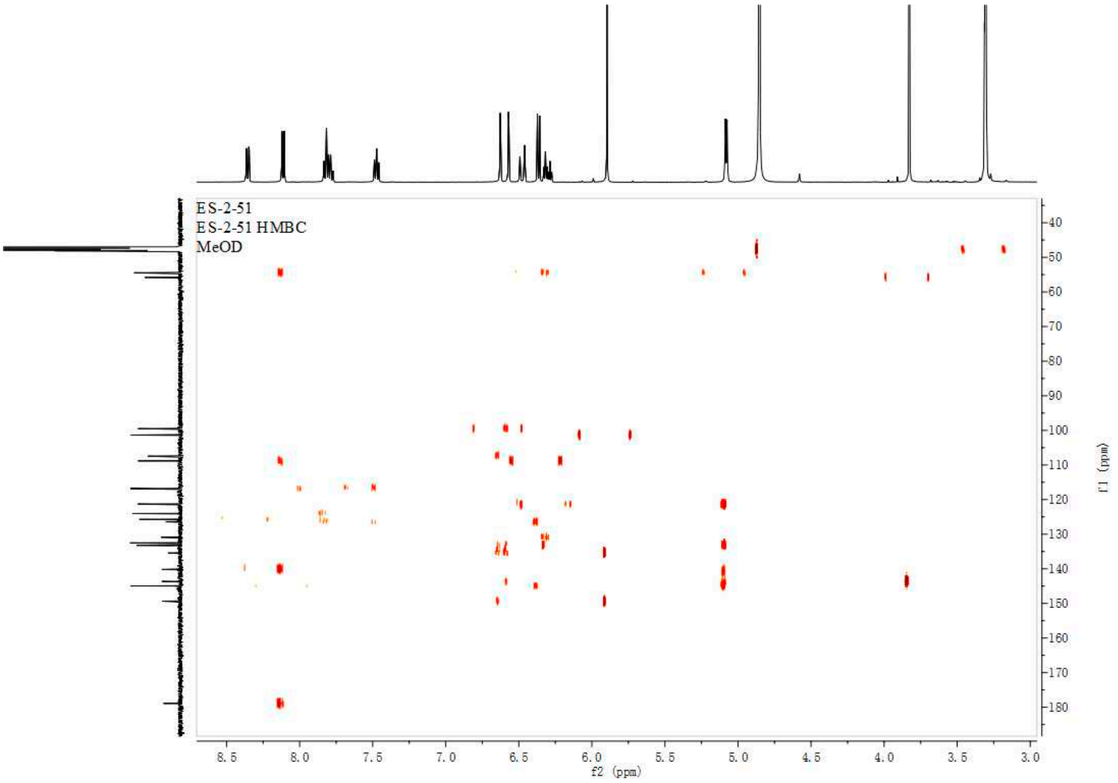

Figure S5. HMBC spectrum of **1**

Display Report

Analysis Info

Analysis Name	D:\Data\GJH\LYL\20221013\ES-2-51.d	Acquisition Date	10/13/2022 5:21:10 PM
Method	tune_pos_standard_20141031.m	Operator	Demo User
Sample Name	ES-2-51	Instrument	maXis HD 1820881.21303
Comment			

Acquisition Parameter

Source Type	ESI	Ion Polarity	Positive	Set Nebulizer	0.3 Bar
Focus	Active	Set Capillary	3500 V	Set Dry Heater	200 °C
Scan Begin	50 m/z	Set End Plate Offset	-500 V	Set Dry Gas	4.0 l/min
Scan End	3000 m/z	Set Charging Voltage	2000 V	Set Divert Valve	Waste
		Set Corona	0 nA	Set APCI Heater	0 °C

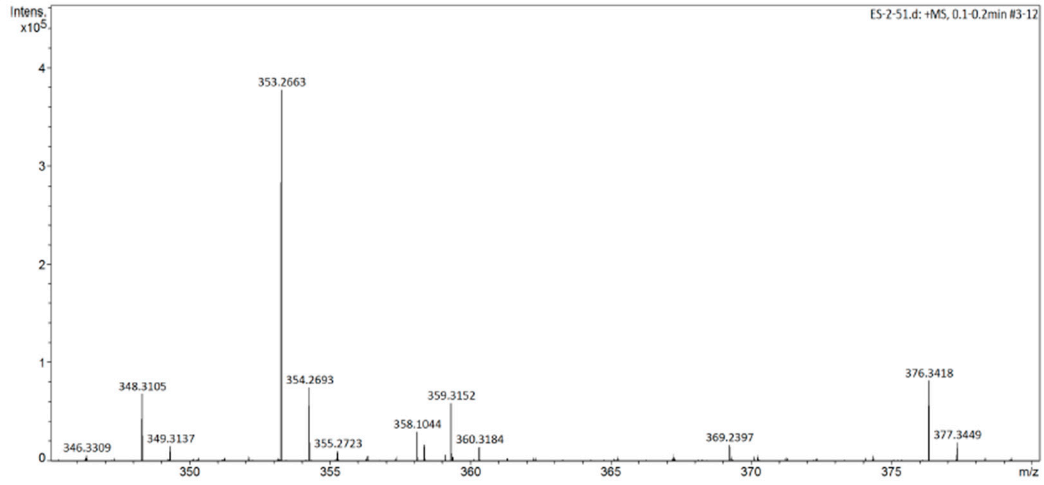

ES-2-51.d

Bruker Compass DataAnalysis 4.4 printed: 12/26/2022 5:31:28 PM by: demo Page 1 of 1

激活 Win

Figure S6. HR-ESI-MS spectrum of compound **1**

Thermo Scientific ~ VISIONpro SOFTWARE V4.41

Operator Name	(None Entered)	Date of Report	2022/12/11
Department	(None Entered)	Time of Report	22:48:05下午
Organization	(None Entered)		
Information	(None Entered)		

Scan Graph

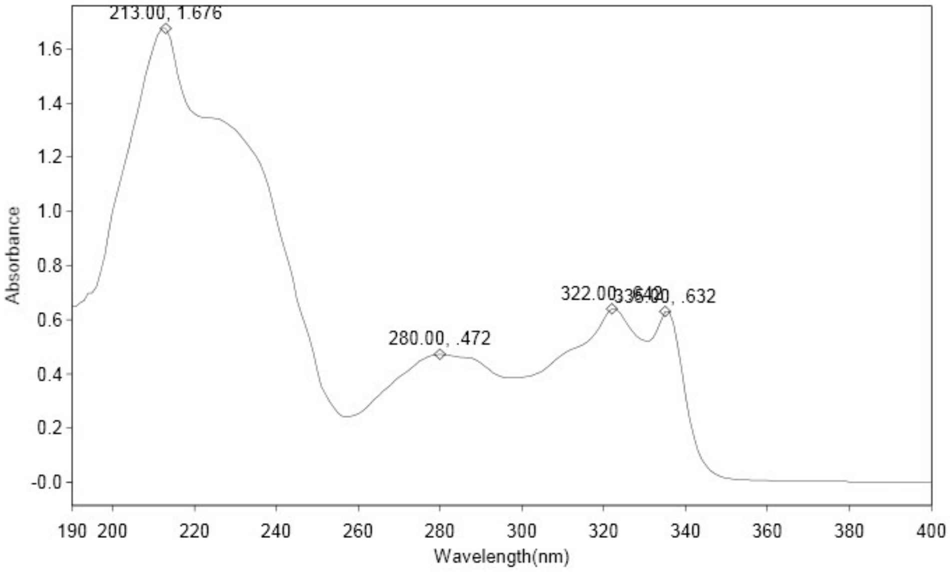

Results Table - ES-2-51.sre,ES-2-51,Cycle01

nm	A	**Peak Pick Method**
213.00	1.676	Find 8 Peaks Above -3.0000 A
280.00	.472	Start Wavelength190.00 nm
322.00	.642	Stop Wavelength400.00 nm
335.00	.632	Sort By Wavelength
Sensitivity	High	

Figure S7. UV spectrum of **1**

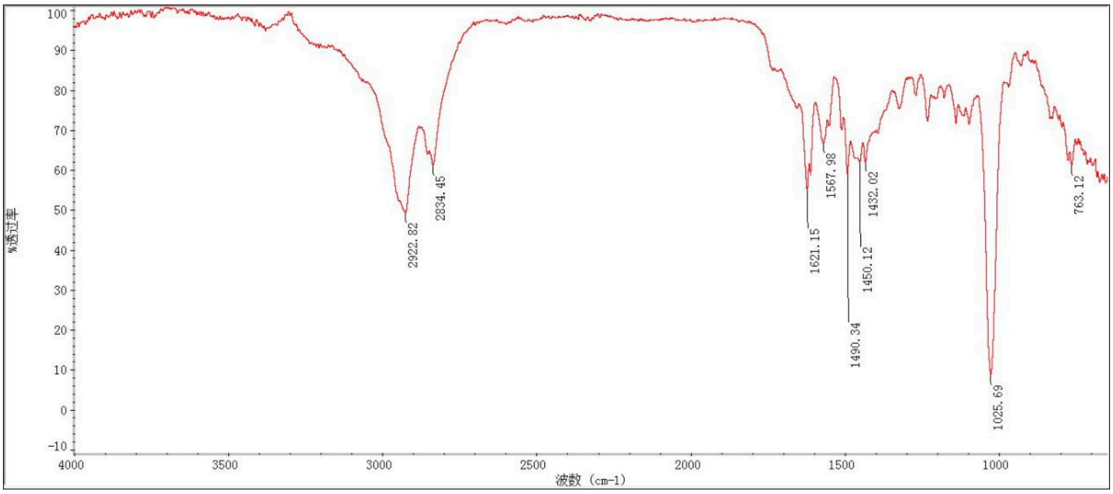

Figure S8. IR spectrum of **1**

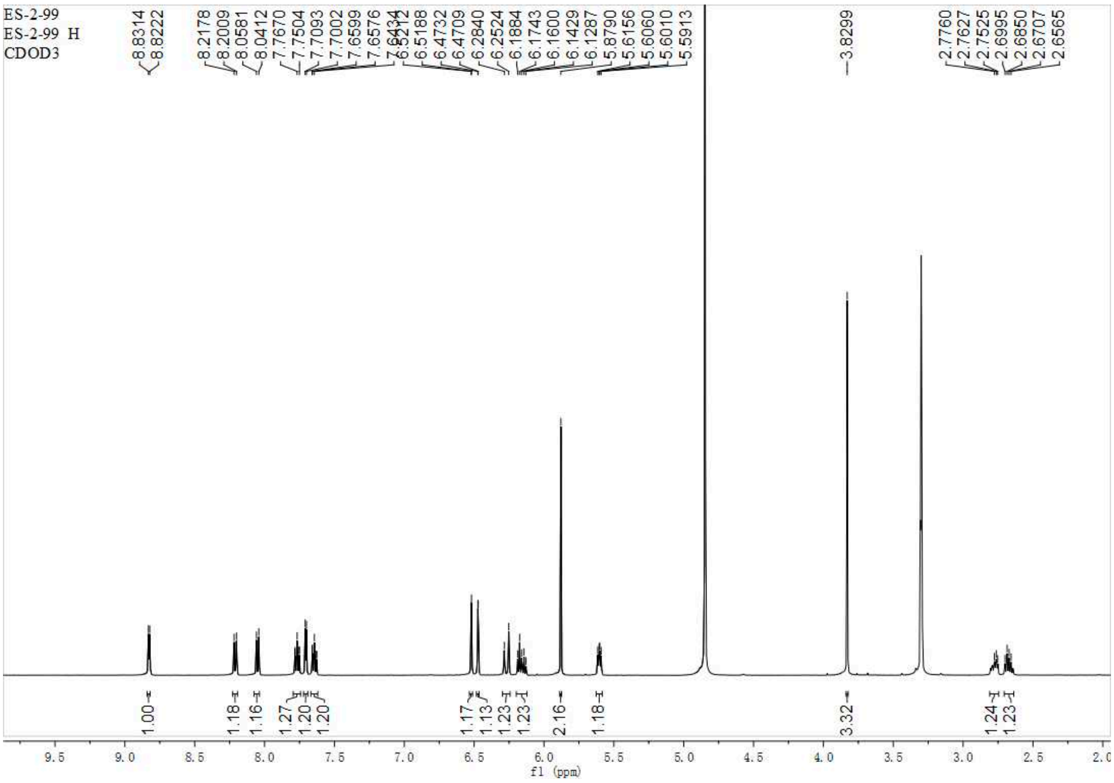

Figure S9. ^{1}H NMR spectrum (500MHz, CD_3OD) of **2**

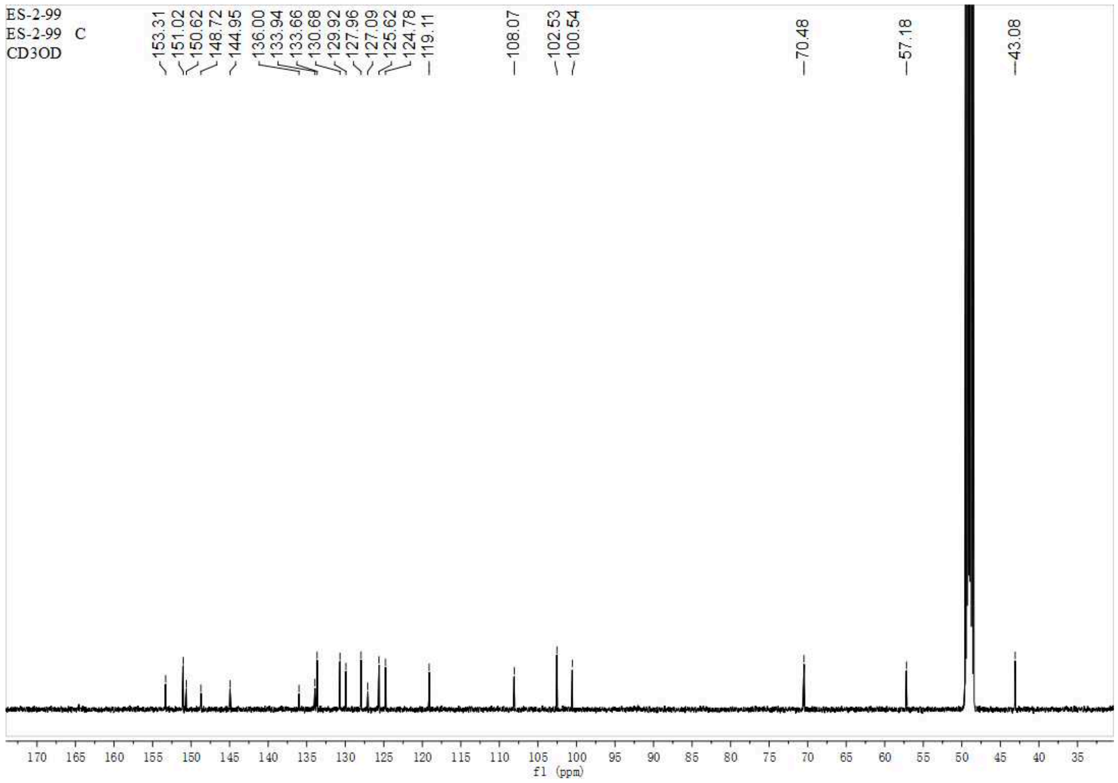

Figure S10. ^{13}C NMR spectrum (125MHz, CD_3OD) of **2**

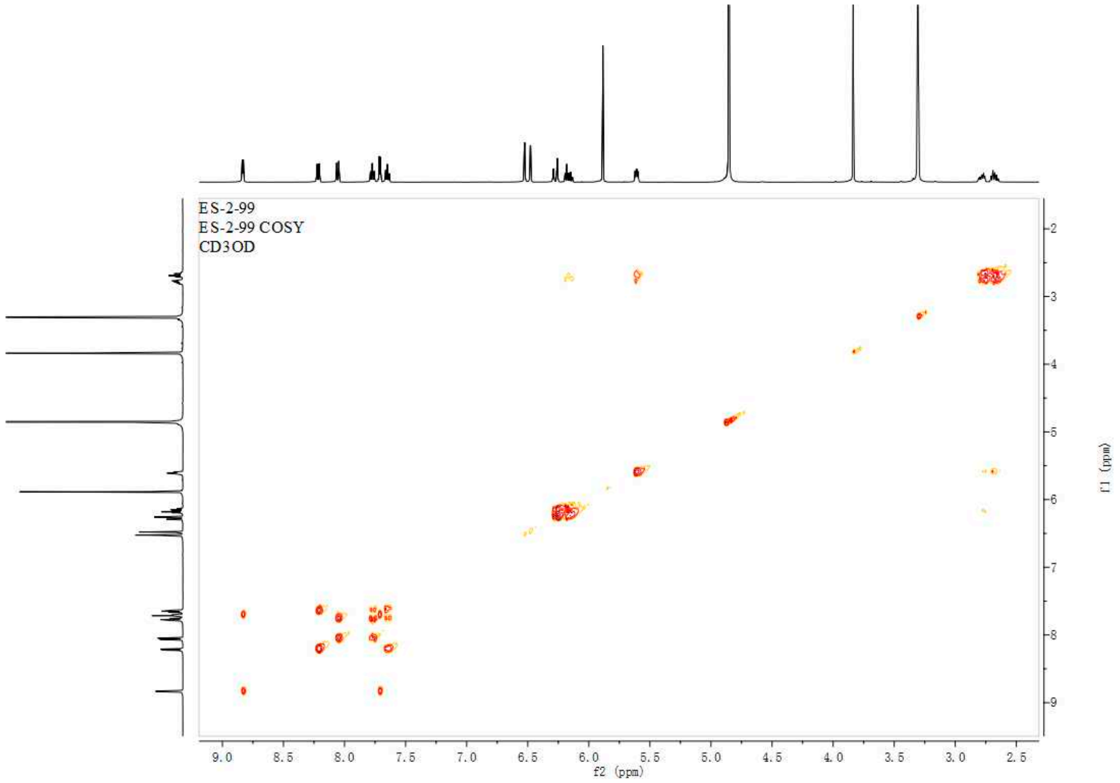

Figure S11. ^1H-^1H COSY spectrum of **2**

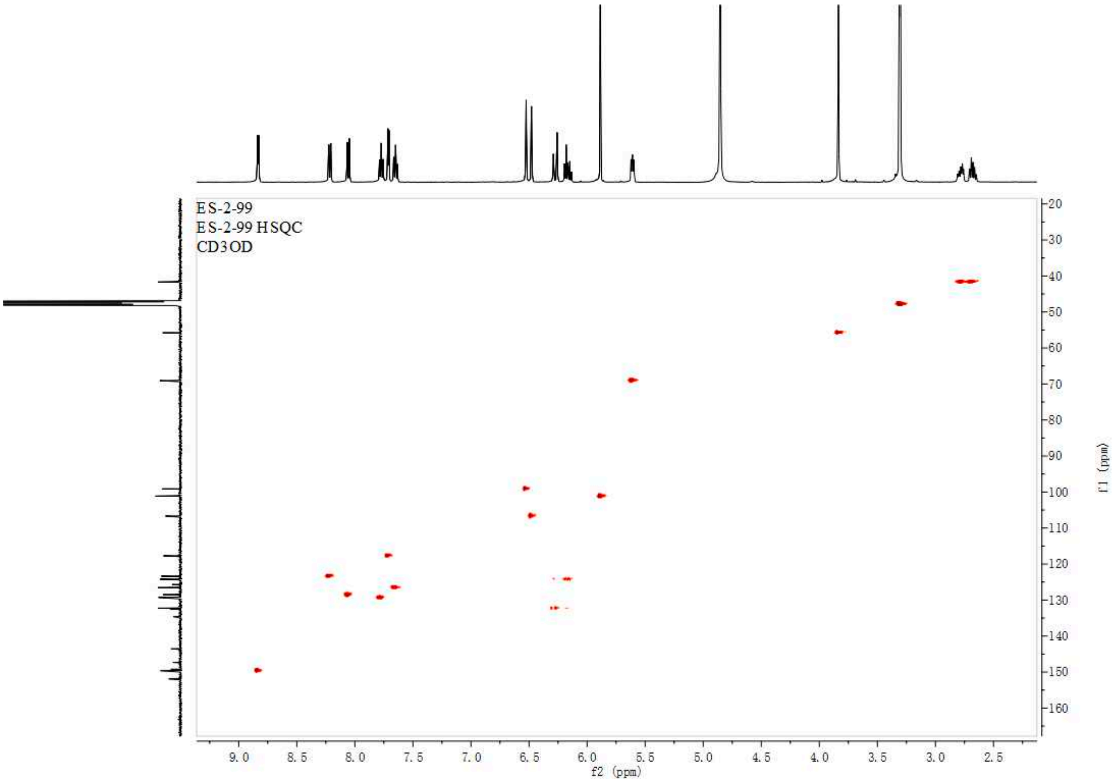

Figure S12. HSQC spectrum of **2**

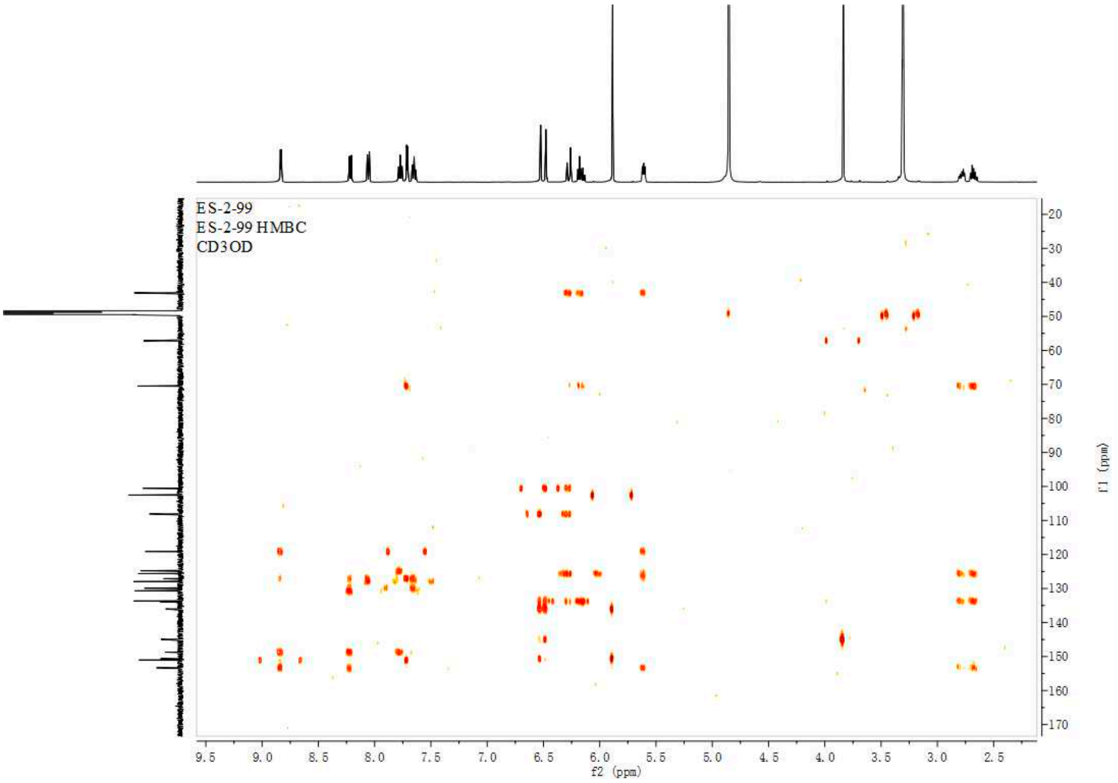

Figure S13. HMBC spectrum of **2**

Display Report

Analysis Info
Analysis Name \\ESI-PC\Data\GJH\20240203\ES-2-99.d
Method tune_pos_standard_20141031.m
Sample Name ES-2-99
Comment

Acquisition D 2024/2/3 11:46:06
Operator Demo User
Instrumen maXis HD 1820881.21303

Acquisition Paramet

Source Type	ESI	Ion Polarity	Positive	Set Nebulizer	0.3 Bar
Focus	Active	Set Capillary	3500 V	Set Dry Heater	200 °C
Scan Begin	50 m/z	Set End Plate Offset	-500 V	Set Dry Gas	4.0 l/min
Scan End	3000 m/z	Set Charging Voltage	2000 V	Set Divert Valve	Waste
		Set Corona	0 nA	Set APCI Heater	0 °C

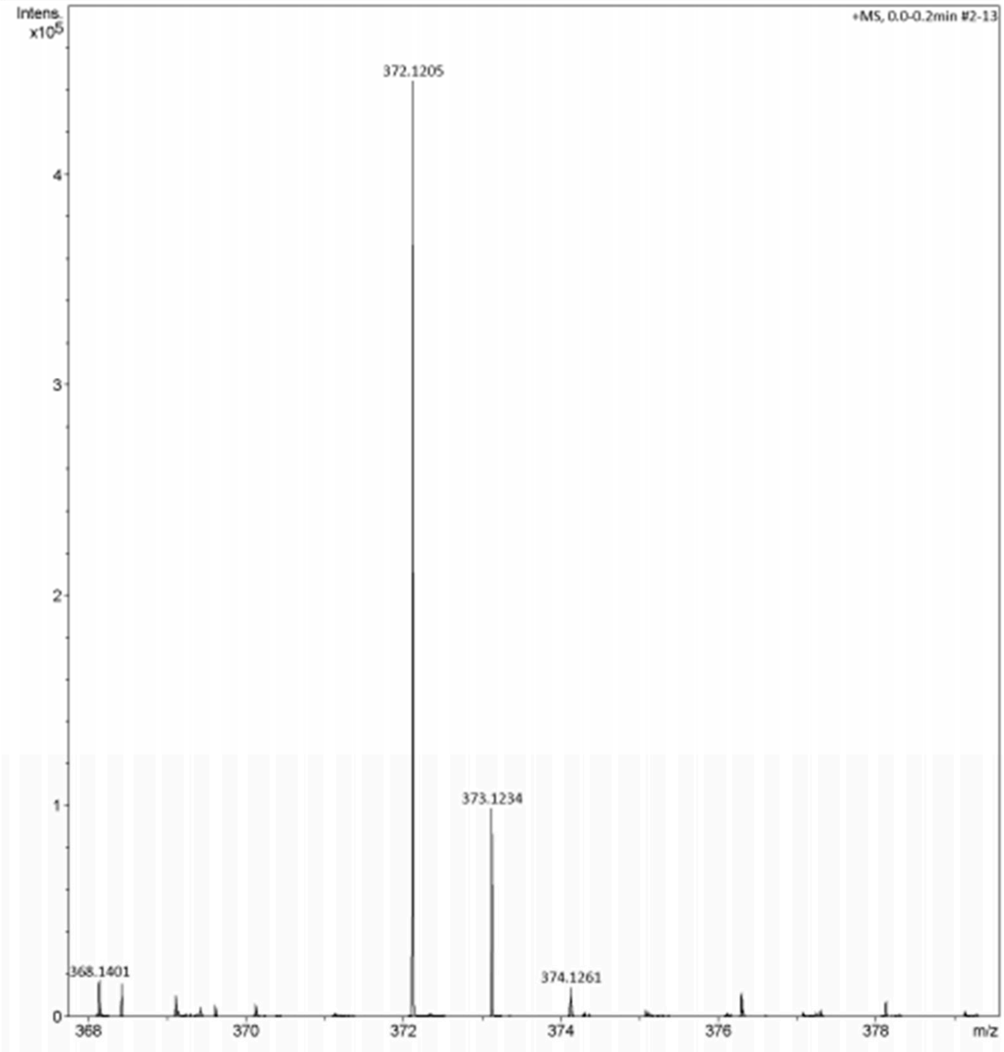

ES-2-99.d
Bruker Compass DataAnalysis 4.2 printe 2024/2/17 10:03:00 by: 1823325 Page 1 of 1

Figure S14. HR-ESI-MS spectrum of compound **2**

Thermo Scientific ~ VISIONpro SOFTWARE V4.41

Operator Name	(None Entered)	Date of Report	2022/12/11
Department	(None Entered)	Time of Report	22:46:28下午
Organization	(None Entered)		
Information	(None Entered)		

Scan Graph

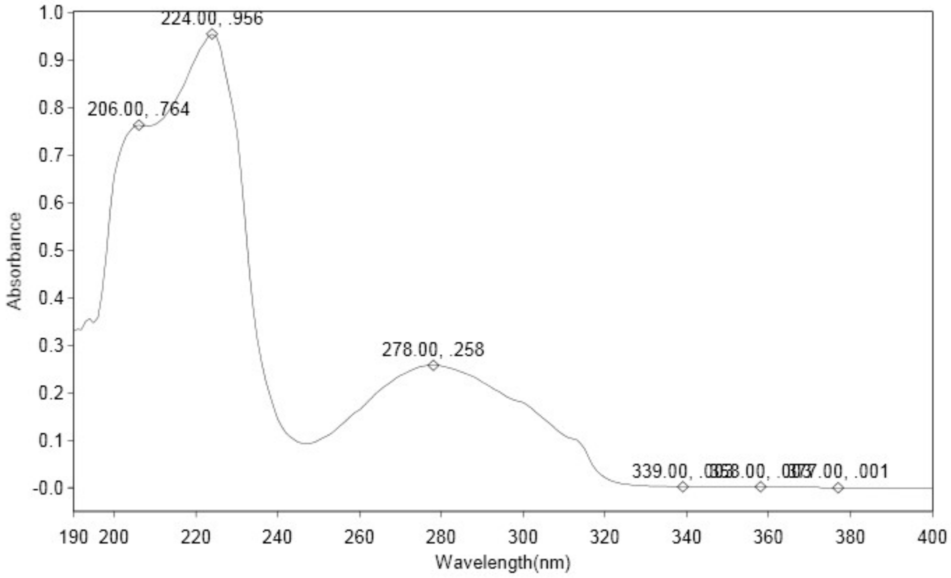

Results Table - ES-2-99.sre,ES-2-99,Cycle01

nm	A	**Peak Pick Method**	
206.00	.764	Find 8 Peaks Above -3.0000 A	
224.00	.956	Start Wavelength 190.00 nm	
278.00	.258	Stop Wavelength 400.00 nm	
339.00	.003	Sort By Wavelength	
358.00	.003	Sensitivity	Manual
377.00	.001	Rising Points	2
Falling Points	2		
Min. Change	0.0000		

Page 1, Scan Graph

Figure S15. UV spectrum of **2**

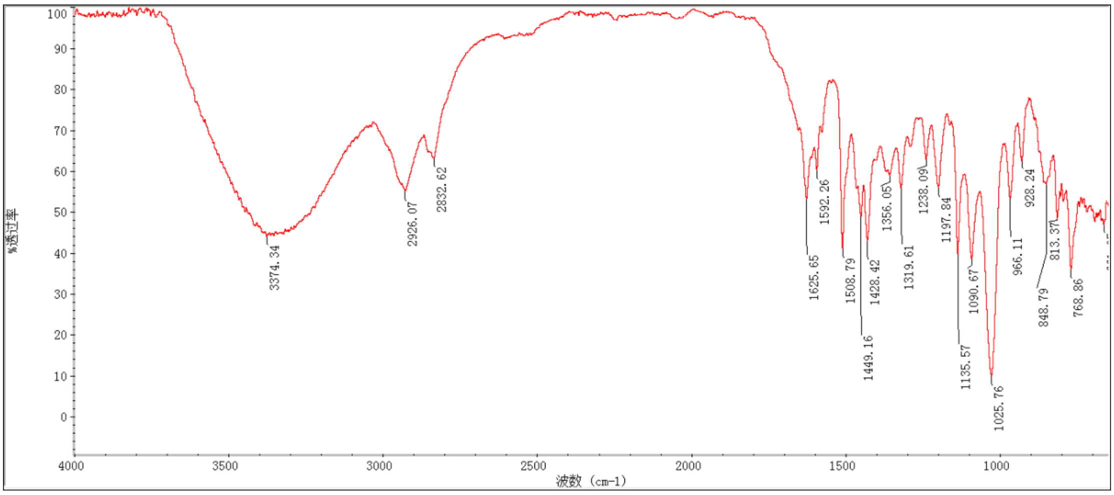

Figure S16. IR spectrum of **2**

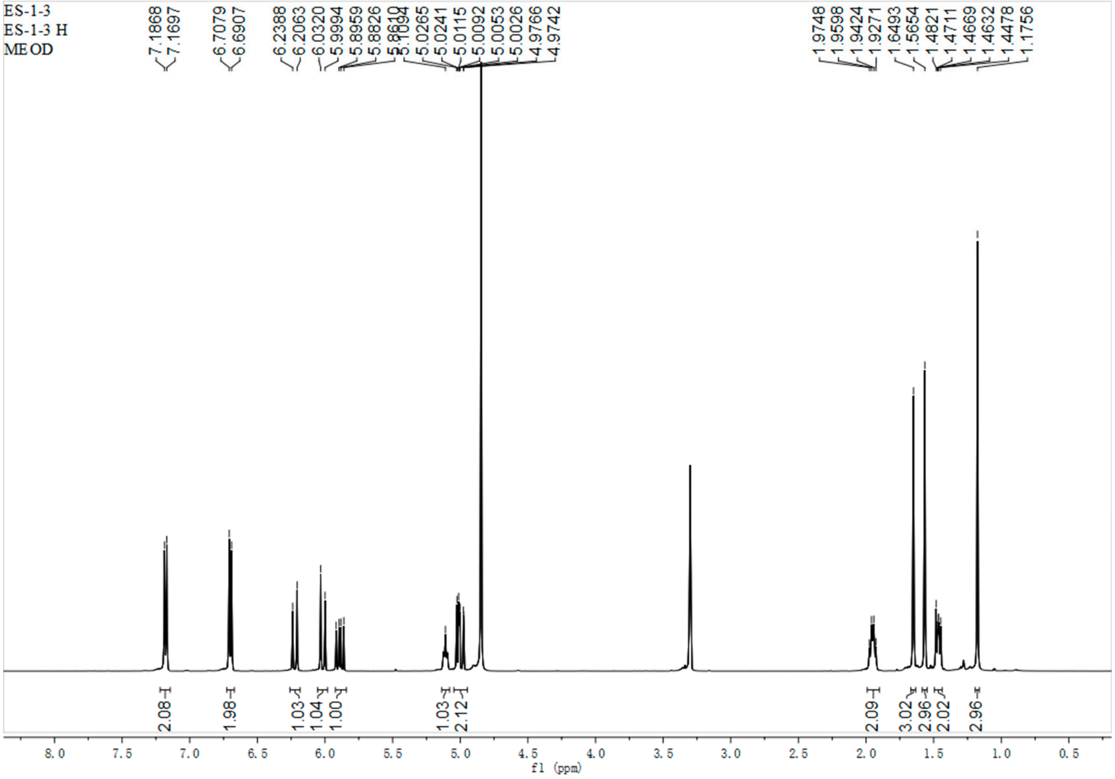

Figure S17. ^{1}H NMR spectrum (500MHz, CD_3OD) of **3**

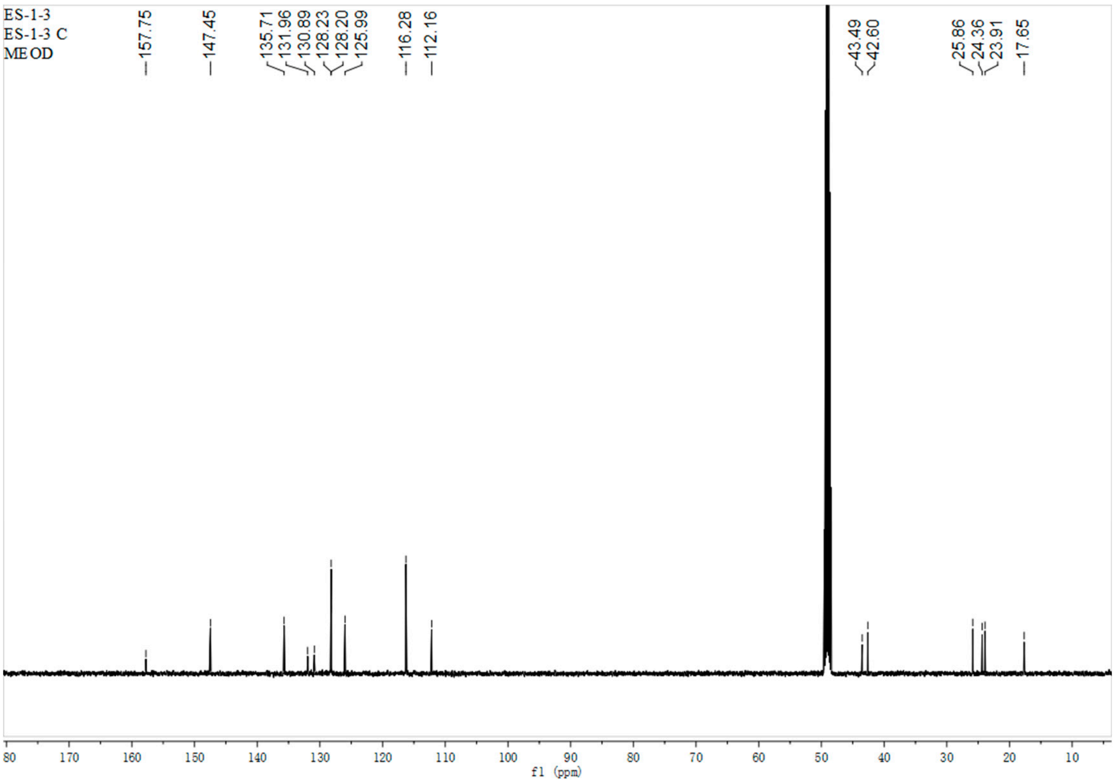

Figure S18. ^{13}C NMR spectrum (125MHz, CD_3OD) of **3**

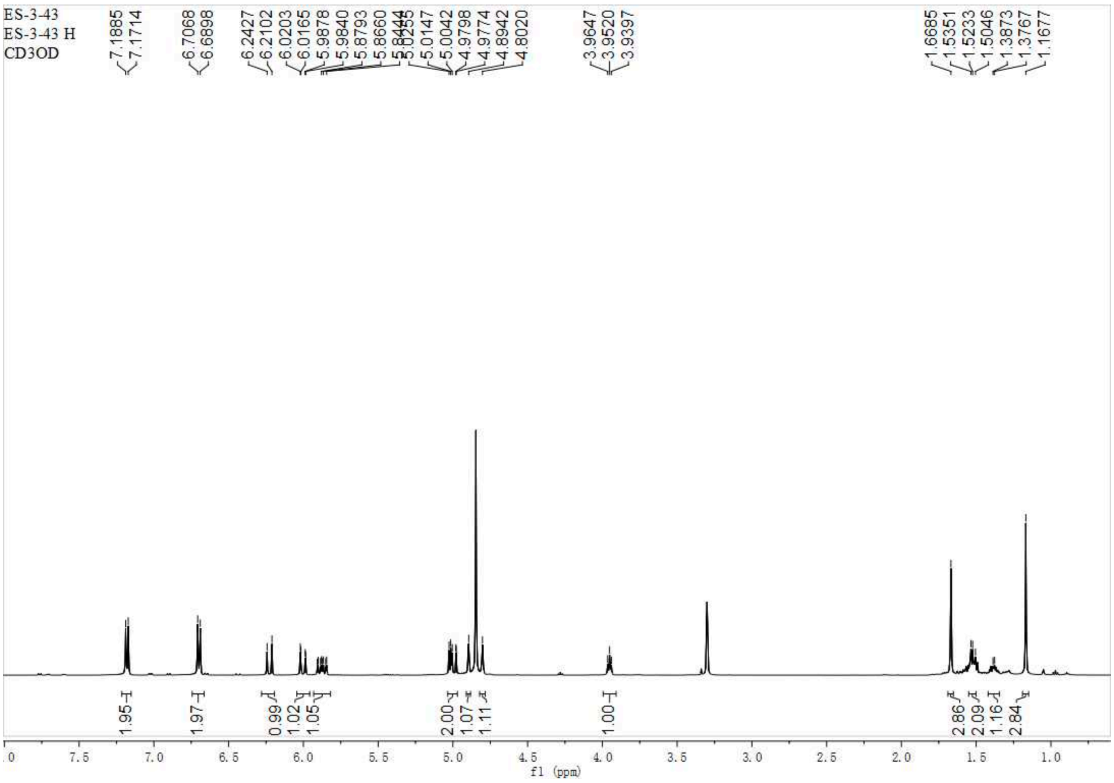

Figure S19. ^{1}H NMR spectrum (500MHz, CD_3OD) of **4**

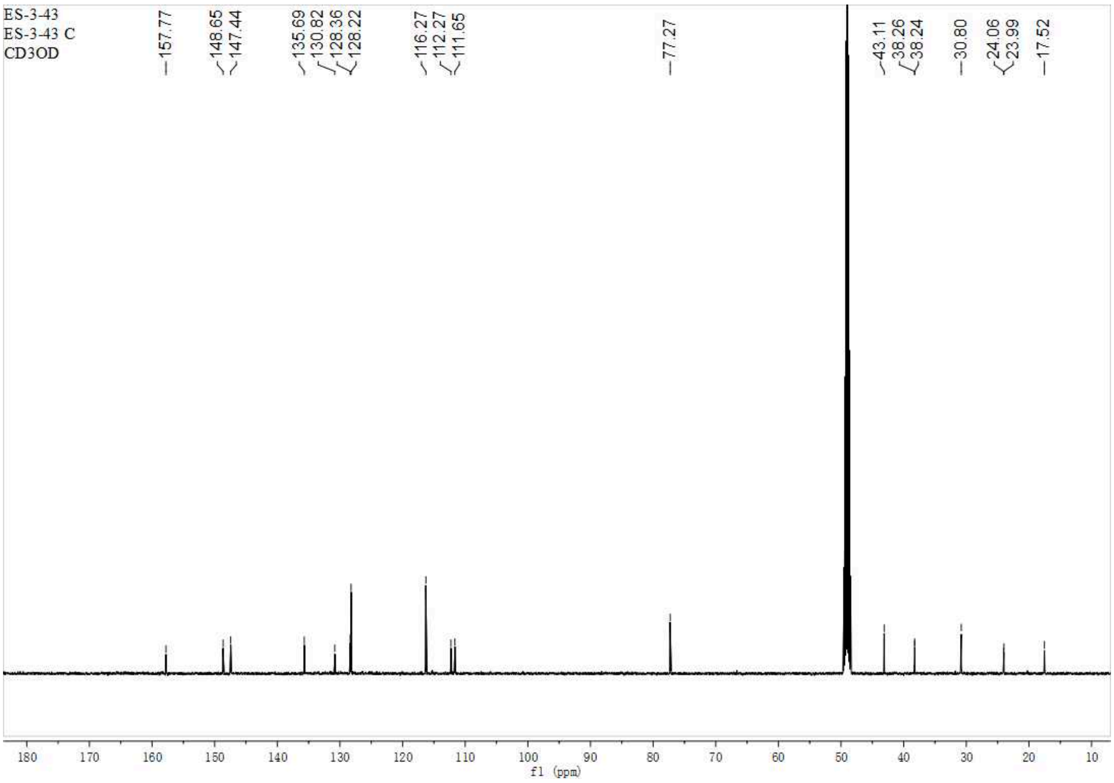

Figure S20. ^{13}C NMR spectrum (125MHz, CD_3OD) of **4**

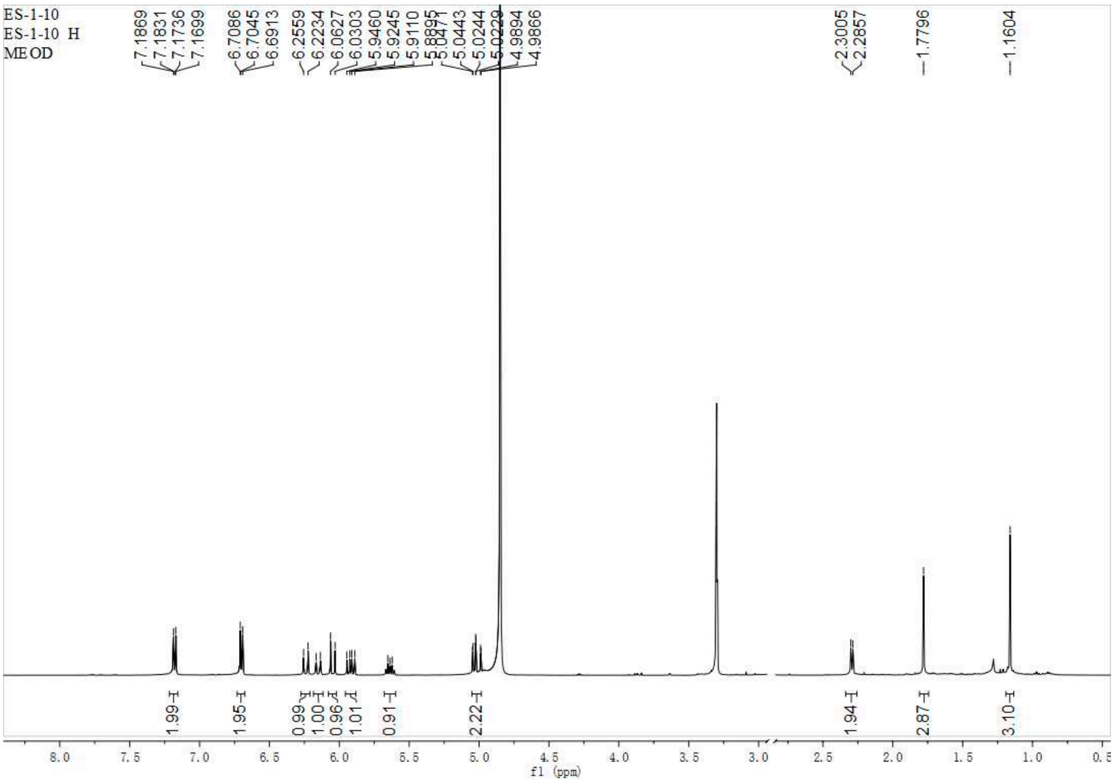

Figure S21. ^{1}H NMR spectrum (500MHz, CD_3OD) of **5**

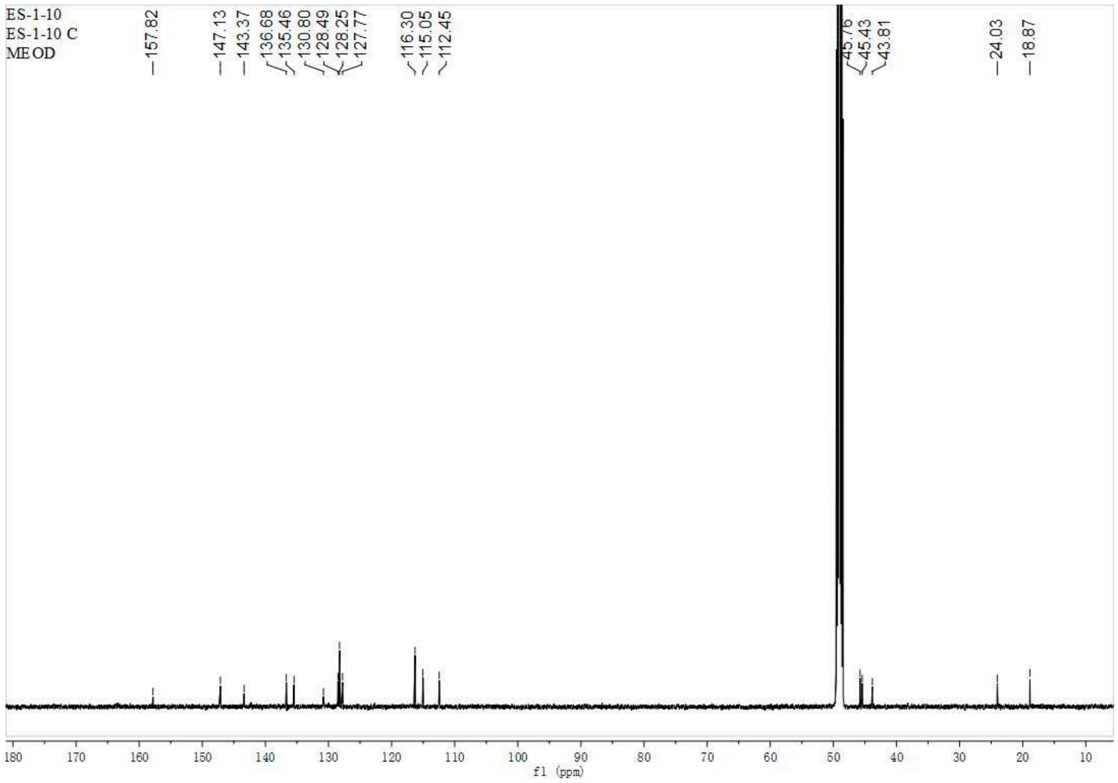

Figure S22 ^{13}C NMR spectrum (125MHz, CD_3OD) of **5**

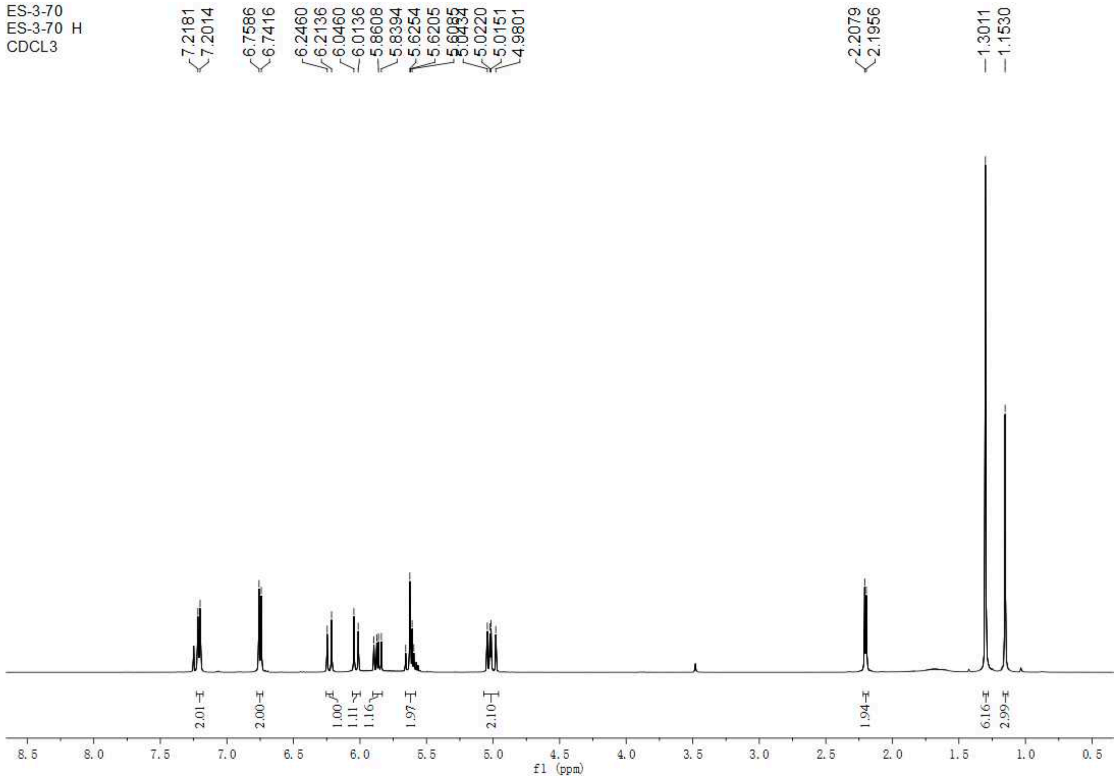

Figure S23. ^{1}H NMR spectrum (500MHz, CD_3OD) of **6**

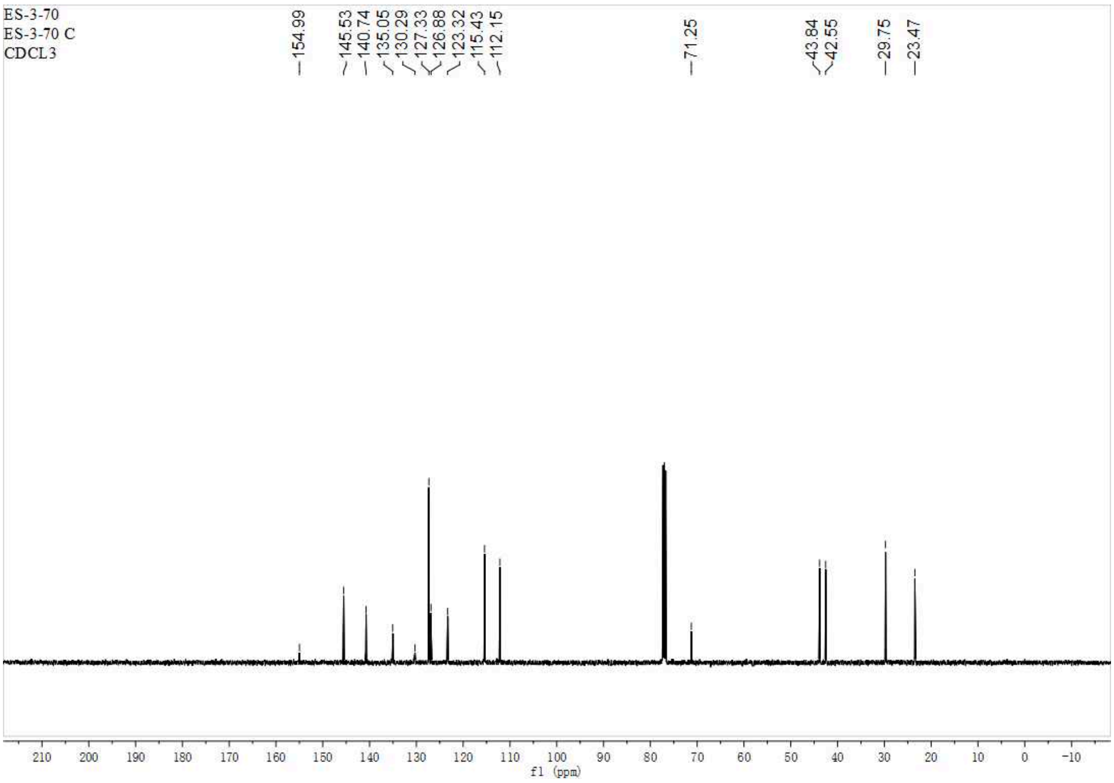

Figure S24. ^{13}C NMR spectrum (125MHz, CD_3OD) of **6**

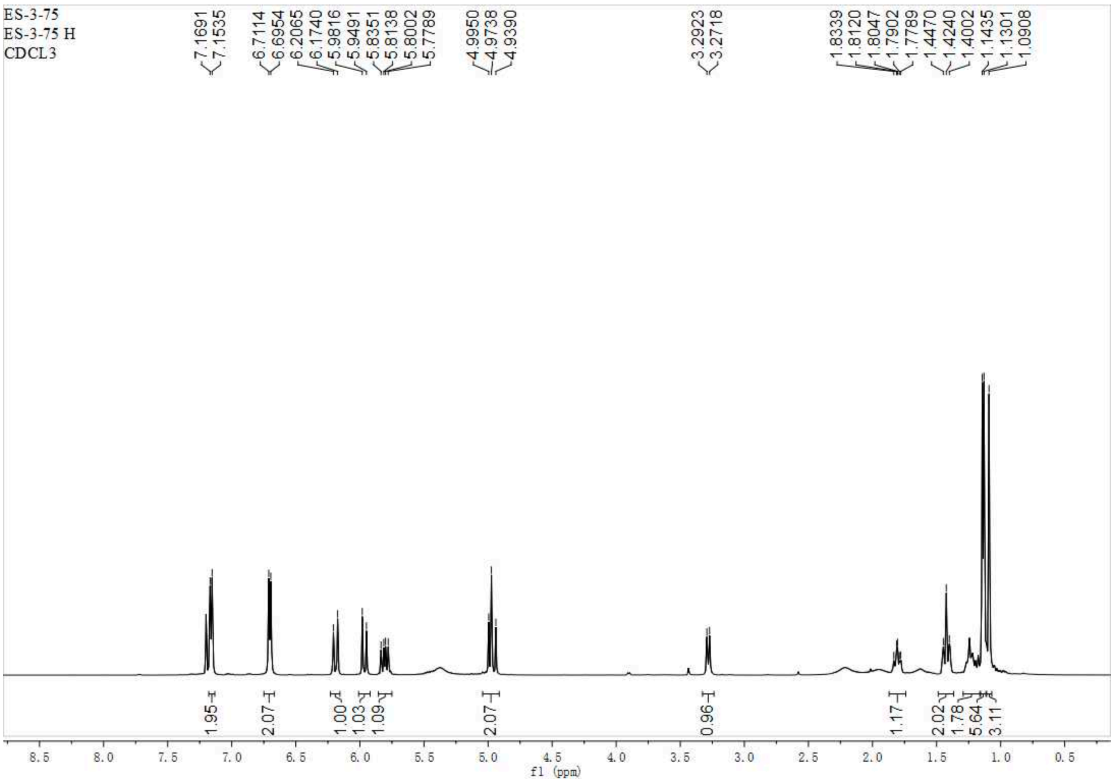

Figure S25. ^{1}H NMR spectrum (500MHz, CD_3OD) of **7**

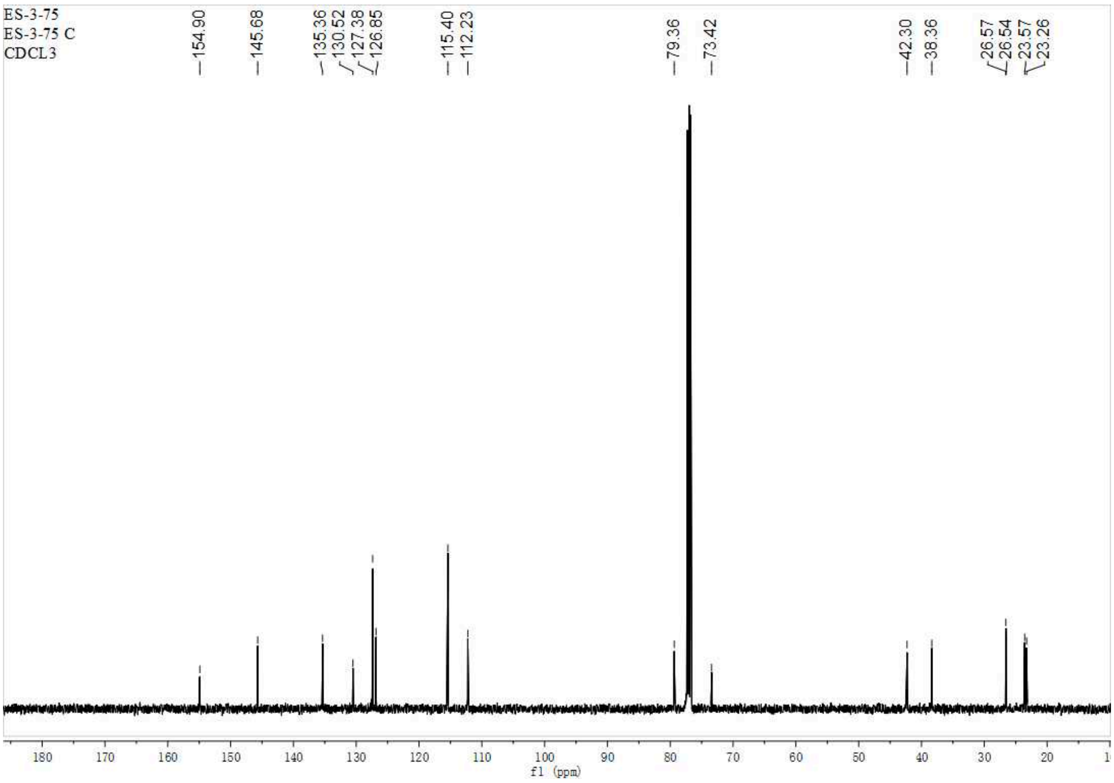

Figure S26. ^{13}C NMR spectrum (125MHz, CD_3OD) of **7**

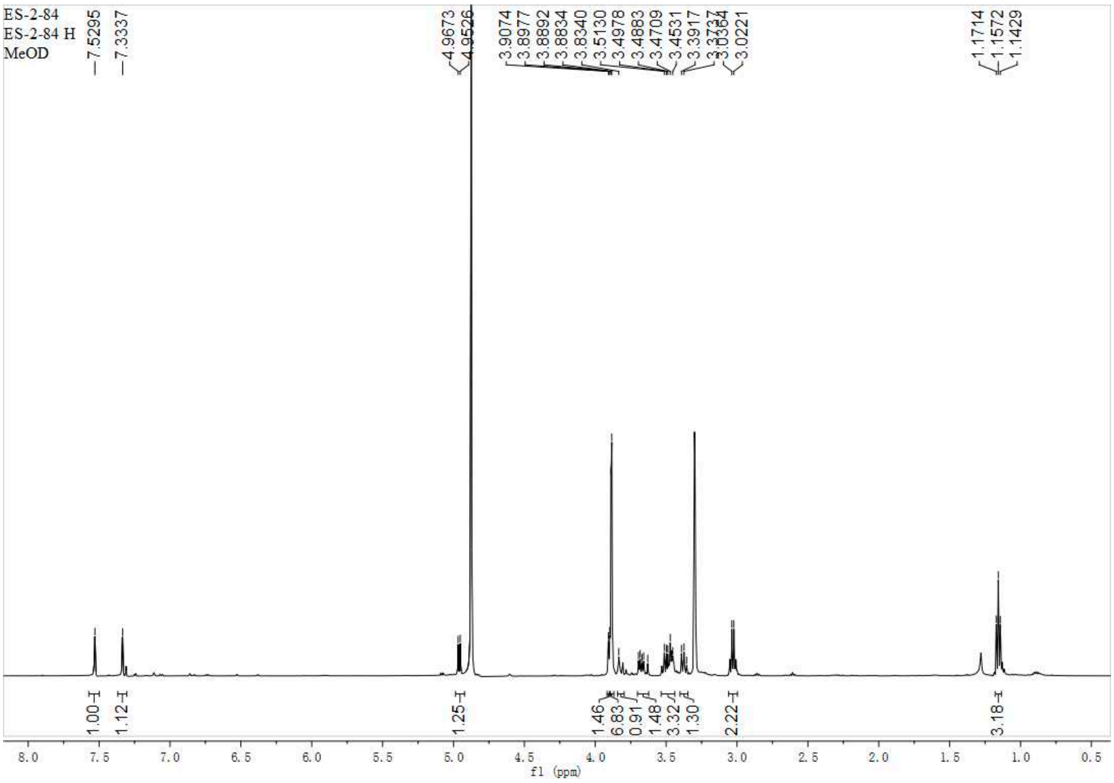

Figure S27. ^{1}H NMR spectrum (500MHz, CD_3OD) of **8**

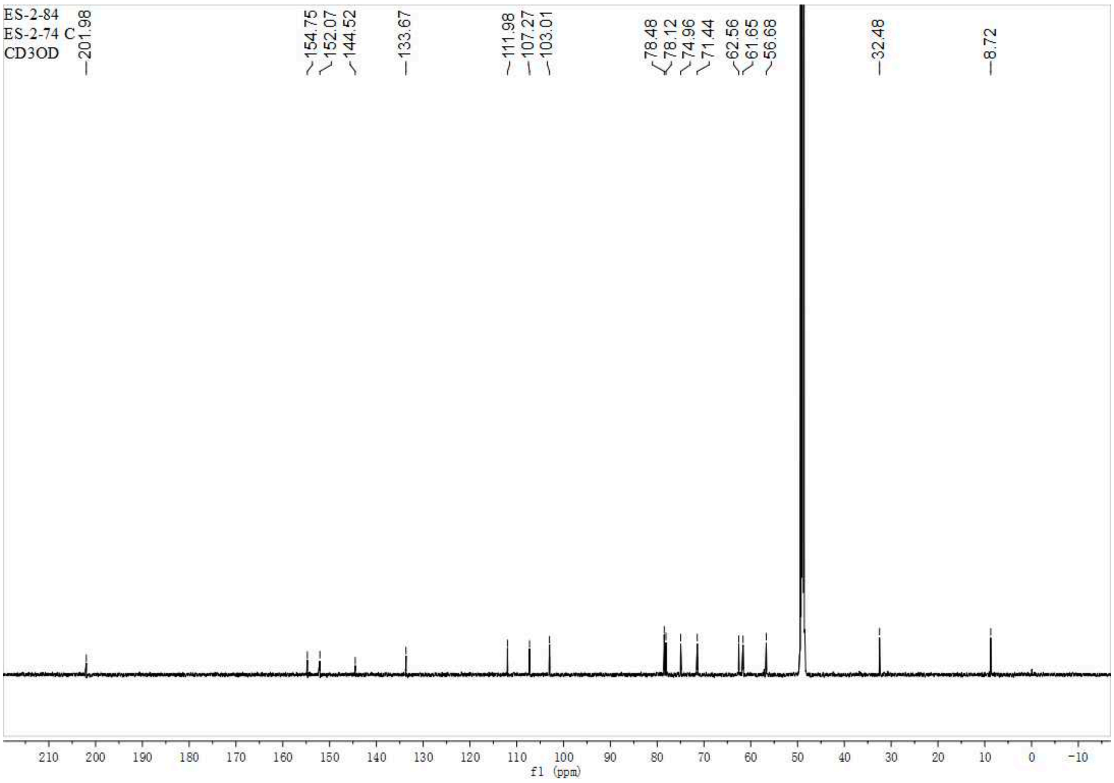

Figure S28. ^{13}C NMR spectrum (125MHz, CD_3OD) of **8**

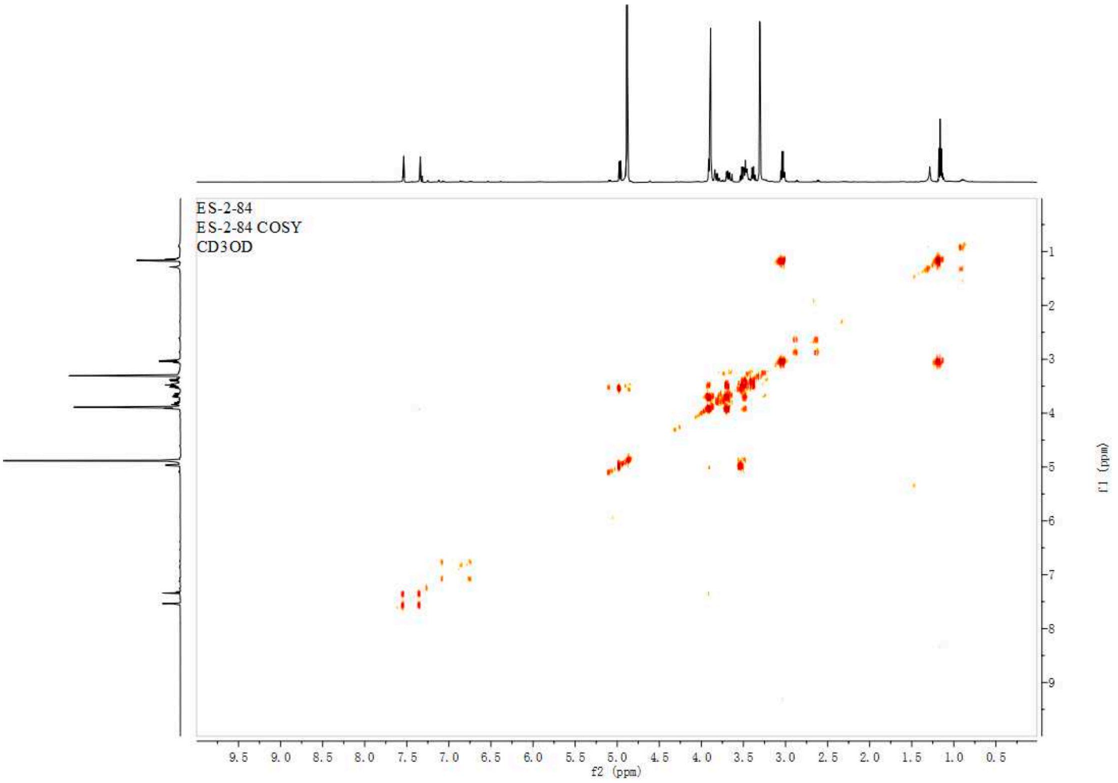

Figure S29. ^1H-^1H COSY spectrum of **8**

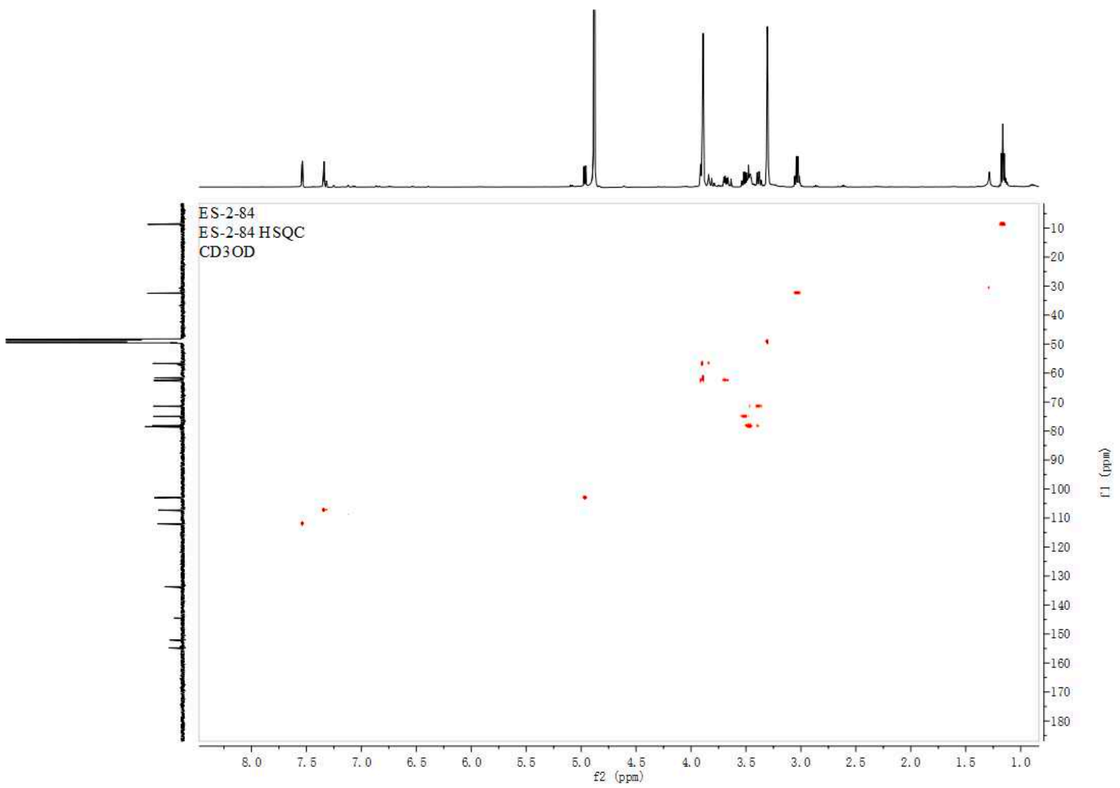

Figure S30. HSQC spectrum of **8**

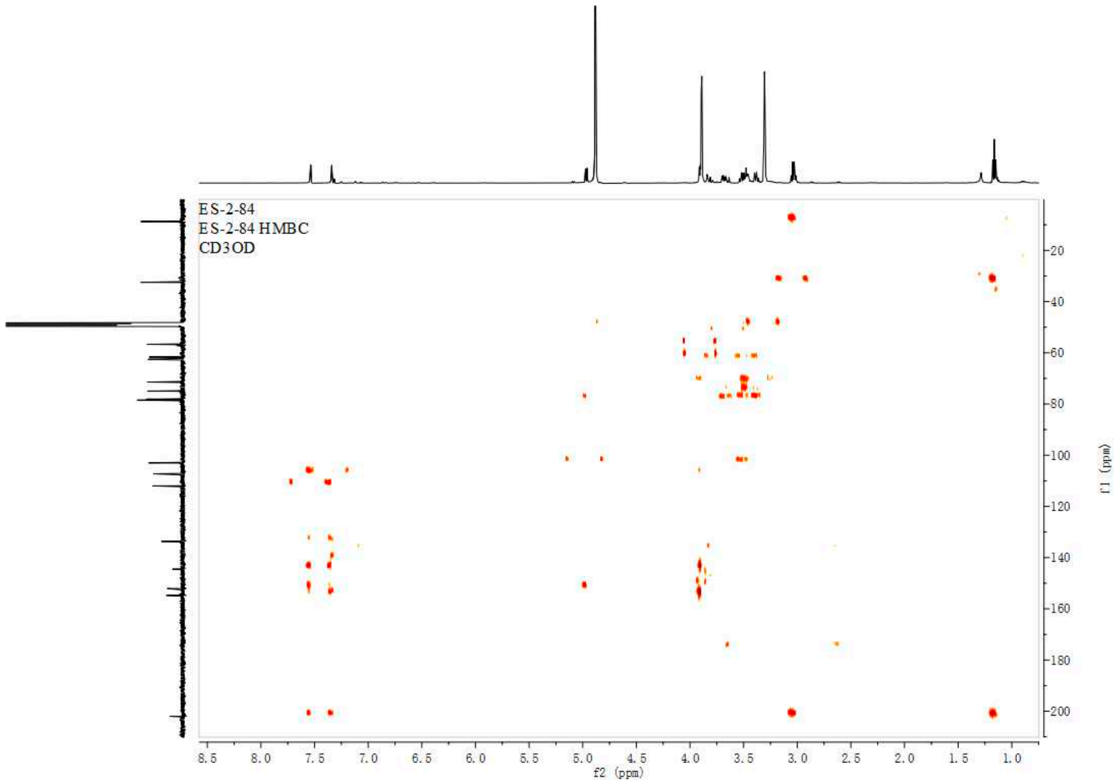

Figure S31. HMBC spectrum of **8**

Display Report

Analysis Info

Analysis Name	D:\Data\GJH\LYL\2023.1.7\ES-2-84.d	Acquisition Date	1/7/2023 5:19:13 PM
Method	tune_pos_standard_20141031.m	Operator	Demo User
Sample Name	ES-2-84	Instrument	maXis HD 1820881.21303
Comment			

Acquisition Parameter

Source Type	ESI	Ion Polarity	Positive	Set Nebulizer	0.3 Bar
Focus	Active	Set Capillary	3500 V	Set Dry Heater	200 °C
Scan Begin	50 m/z	Set End Plate Offset	-500 V	Set Dry Gas	4.0 l/min
Scan End	3000 m/z	Set Charging Voltage	2000 V	Set Divert Valve	Waste
		Set Corona	0 nA	Set APCI Heater	0 °C

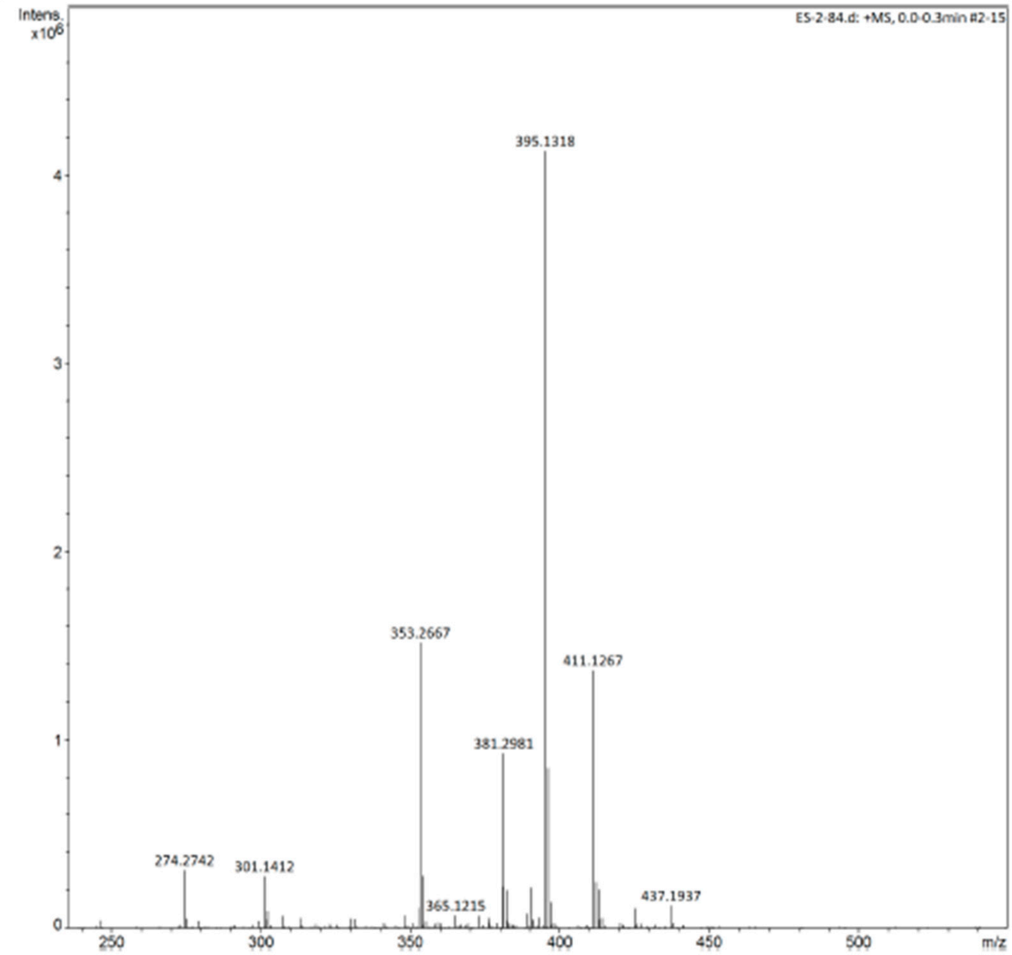

ES-2-84.d

Bruker Compass DataAnalysis 4.4 printed: 1/9/2023 11:11:06 AM by: demo Page 1 of 1

Figure S32. HR-ESI-MS spectrum of compound **8**

Thermo Scientific ~ VISIONpro SOFTWARE V4.41

Operator Name	(None Entered)	Date of Report	2024/1/17
Department	(None Entered)	Time of Report	16:05:35下午
Organization	(None Entered)		
Information	(None Entered)		

Scan Graph

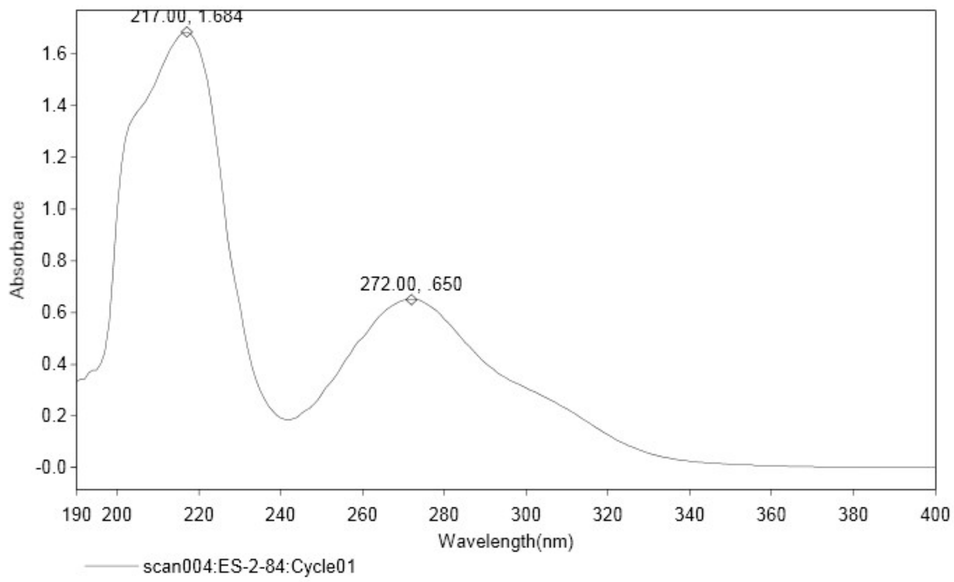

Results Table - ES-2-84.sre,ES-2-84,Cycle01

nm	A	**Peak Pick Method**
217.00	1.684	Find 8 Peaks Above -3.0000 A
272.00	.650	Start Wavelength190.00 nm
		Stop Wavelength400.00 nm
		Sort By Wavelength

Sensitivity Auto

Page 1, Scan Graph

Figure S33. UV spectrum of **8**

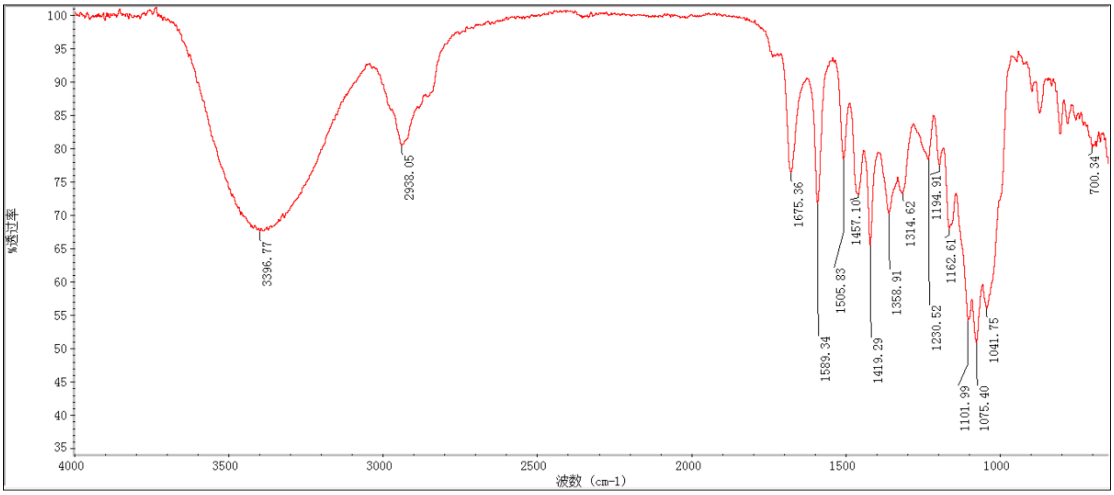

Figure S34. IR spectrum of **8**

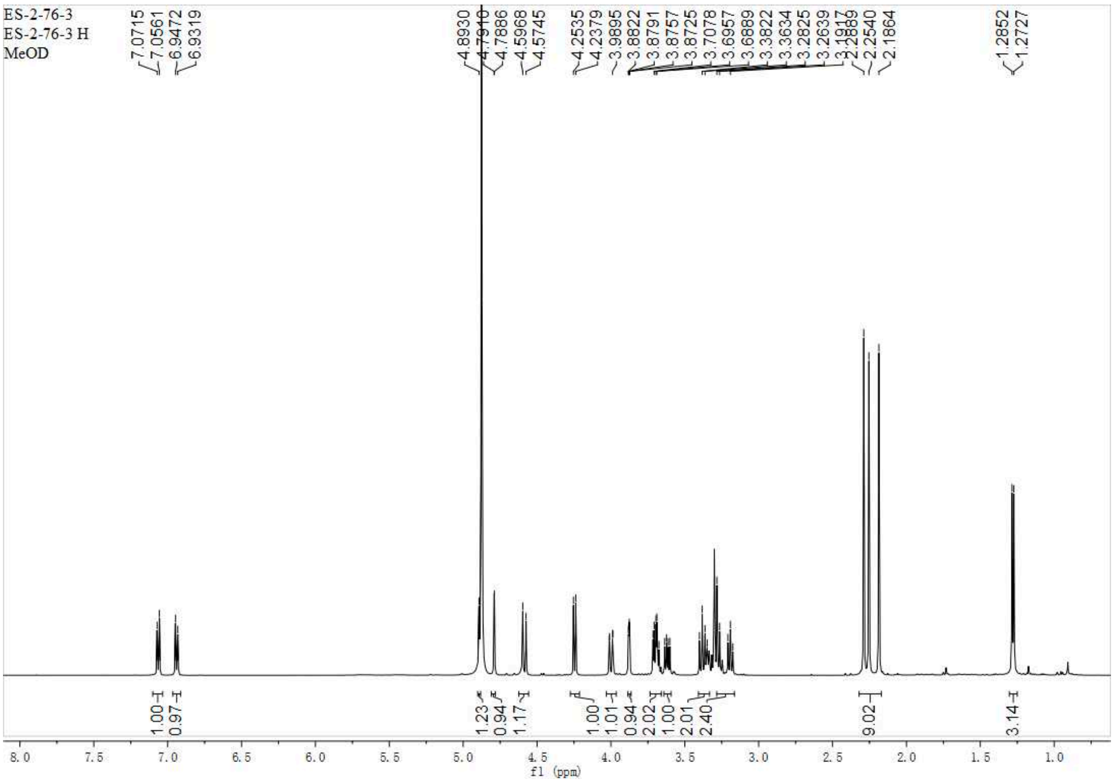

Figure S35. ^{1}H NMR spectrum (500MHz, CD_3OD) of **9**

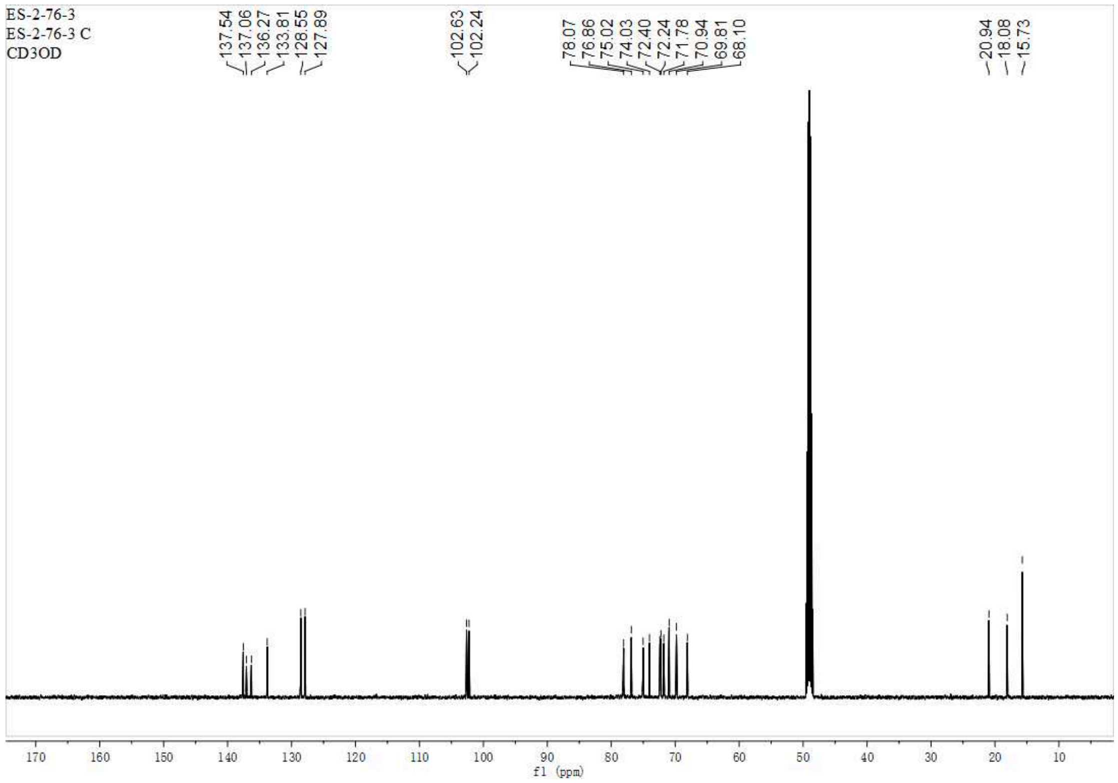

Figure S36. ^{1}H NMR spectrum (500MHz, CD_3OD) of **9**

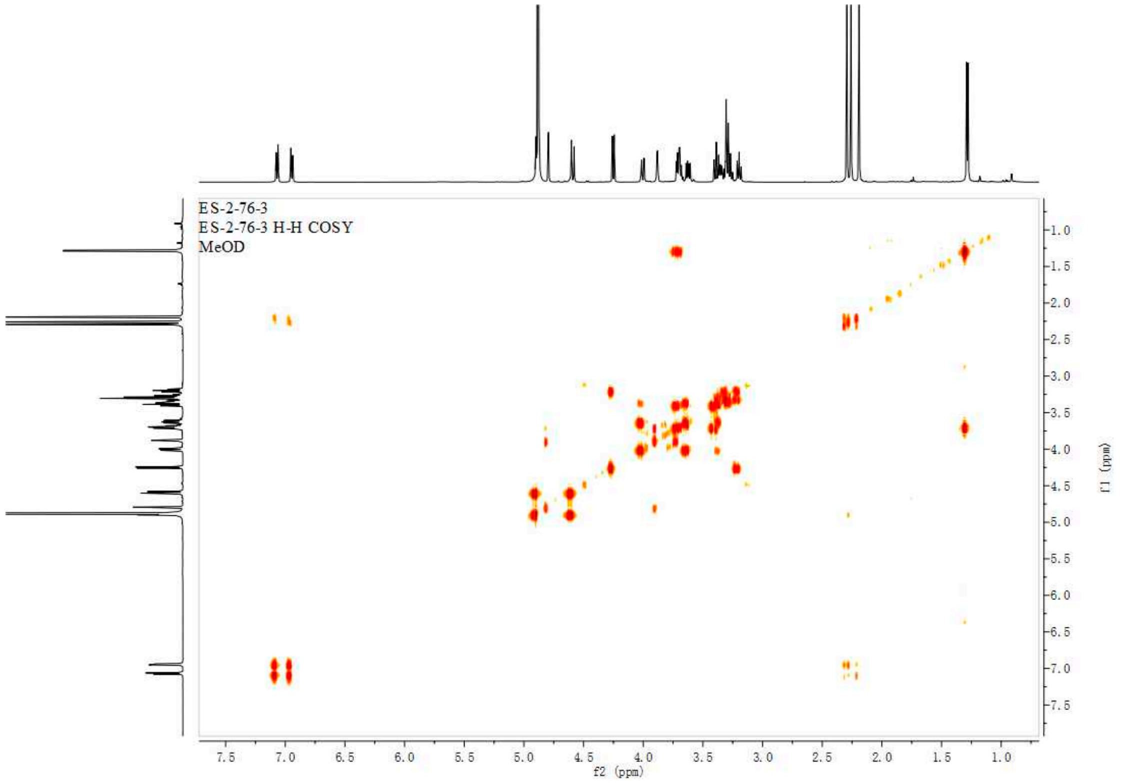

Figure S37. ^{1}H-^{1}H COSY spectrum of **9**

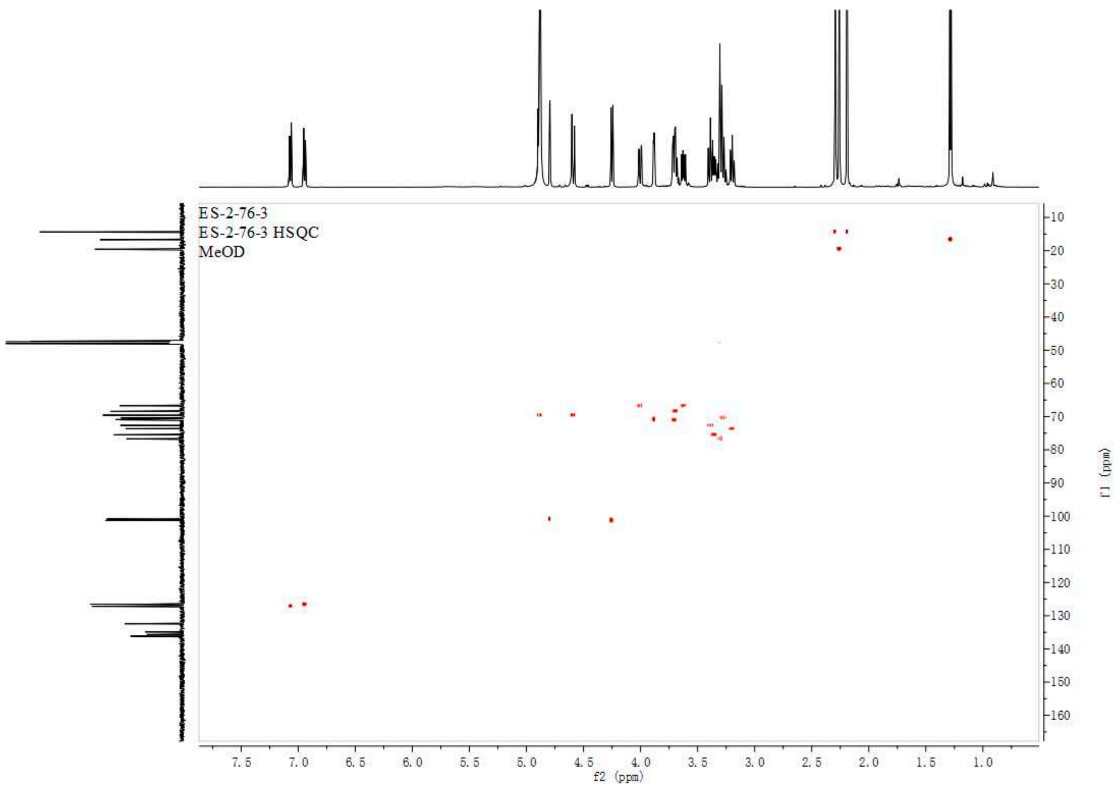

Figure S38. HSQC spectrum of **9**

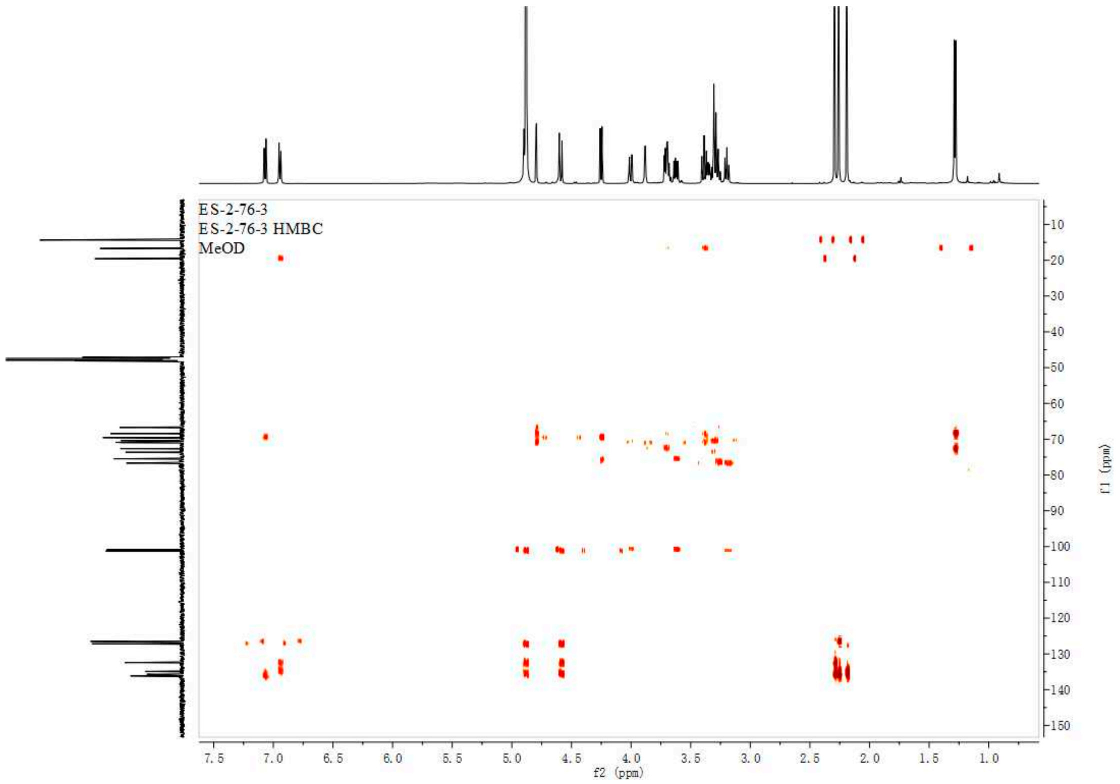

Figure S39. HMBC spectrum of **9**

Display Report

Analysis Info

Analysis Name	D:\Data\GJH\LYL\20221013\ES-2-76-3.d	Acquisition Date	10/13/2022 4:46:57 PM
Method	tune_pos_standard_20141031.m	Operator	Demo User
Sample Name	ES-2-76-3	Instrument	maXis HD 1820881.21303
Comment			

Acquisition Parameter

Source Type	ESI	Ion Polarity	Positive	Set Nebulizer	0.3 Bar
Focus	Active	Set Capillary	3500 V	Set Dry Heater	200 °C
Scan Begin	50 m/z	Set End Plate Offset	-500 V	Set Dry Gas	4.0 l/min
Scan End	3000 m/z	Set Charging Voltage	2000 V	Set Divert Valve	Waste
		Set Corona	0 nA	Set APCI Heater	0 °C

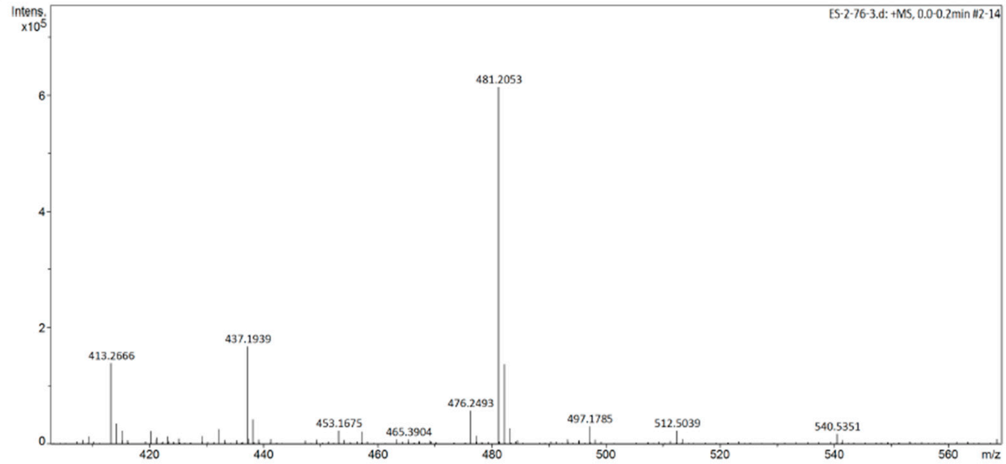

ES-2-76-3.d

Bruker Compass DataAnalysis 4.4 printed: 12/26/2022 8:05:23 PM by: demo Page 1 of 1

Figure S40. HR-ESI-MS spectrum of compound **9**

Thermo Scientific ~ VISIONpro SOFTWARE V4.41

Operator Name	(None Entered)	Date of Report	2024/1/29
Department	(None Entered)	Time of Report	17:15:30下午
Organization	(None Entered)		
Information	(None Entered)		

Scan Graph

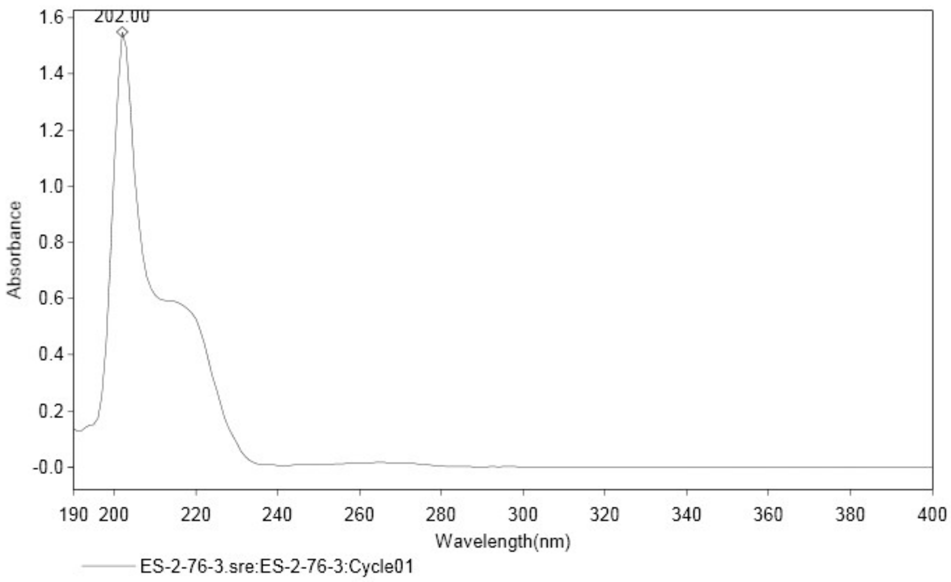

Page 1, Scan Graph

Figure S41 UV spectrum of **9**

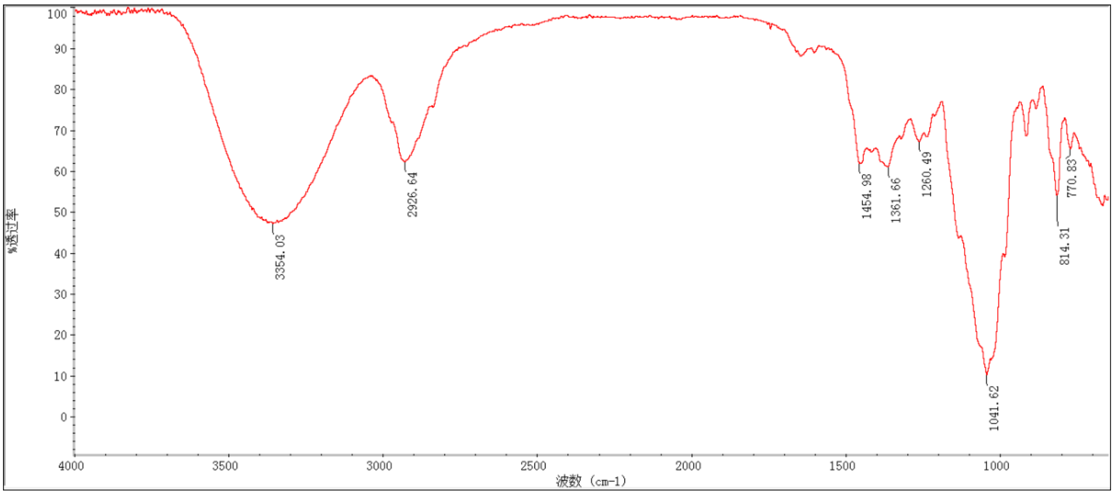

Figure S42. IR spectrum of **9**

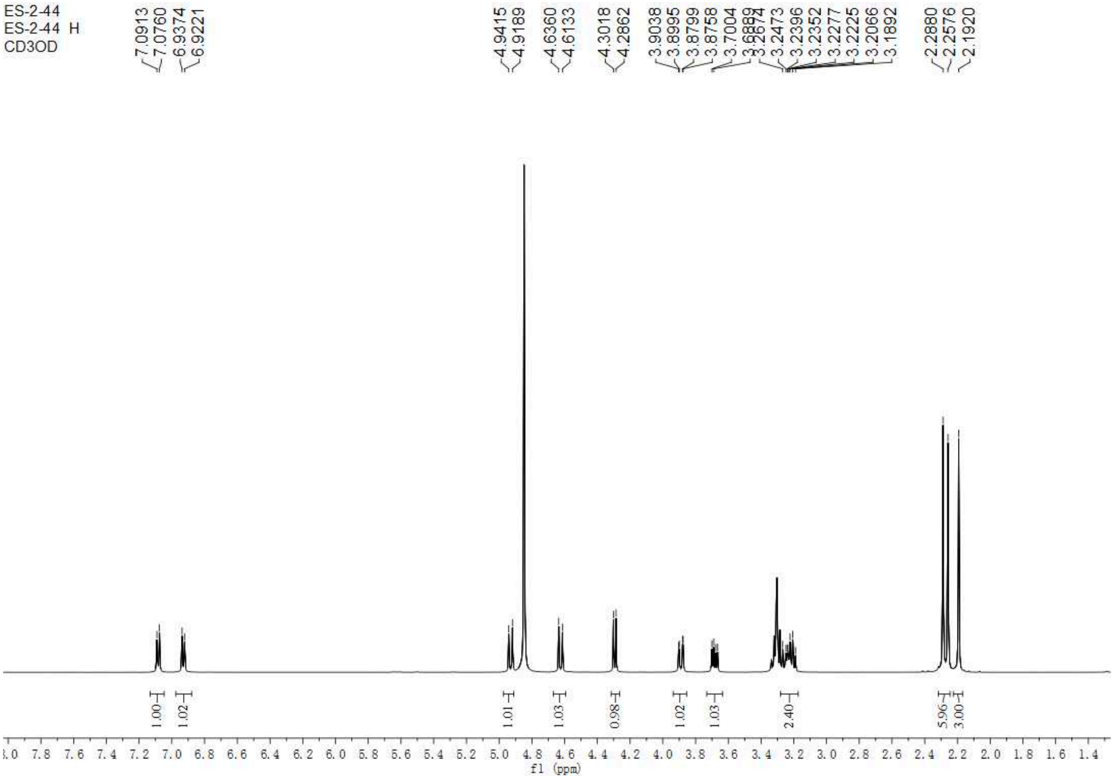

Figure S43. ^{1}H NMR spectrum (500MHz, CD_3OD) of **10**

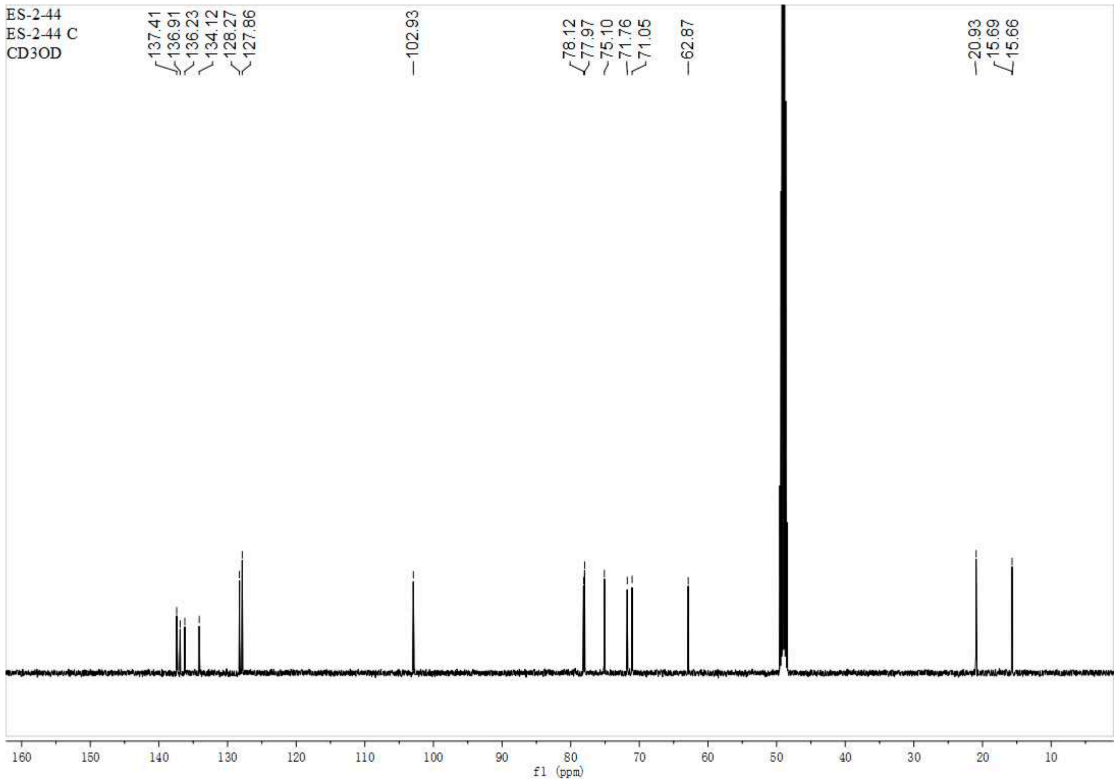

Figure S44. ^{13}C NMR spectrum (125MHz, CD_3OD) of **10**

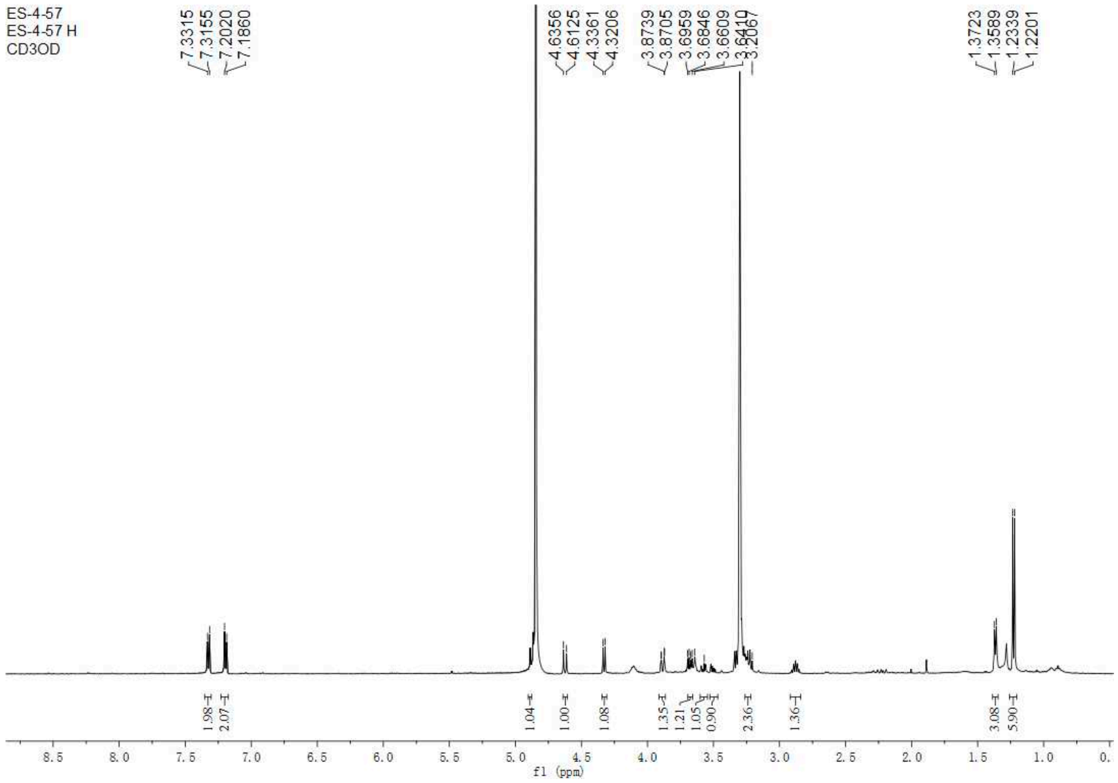

Figure S45. ^{1}H NMR spectrum (500MHz, CD_3OD) of **11**

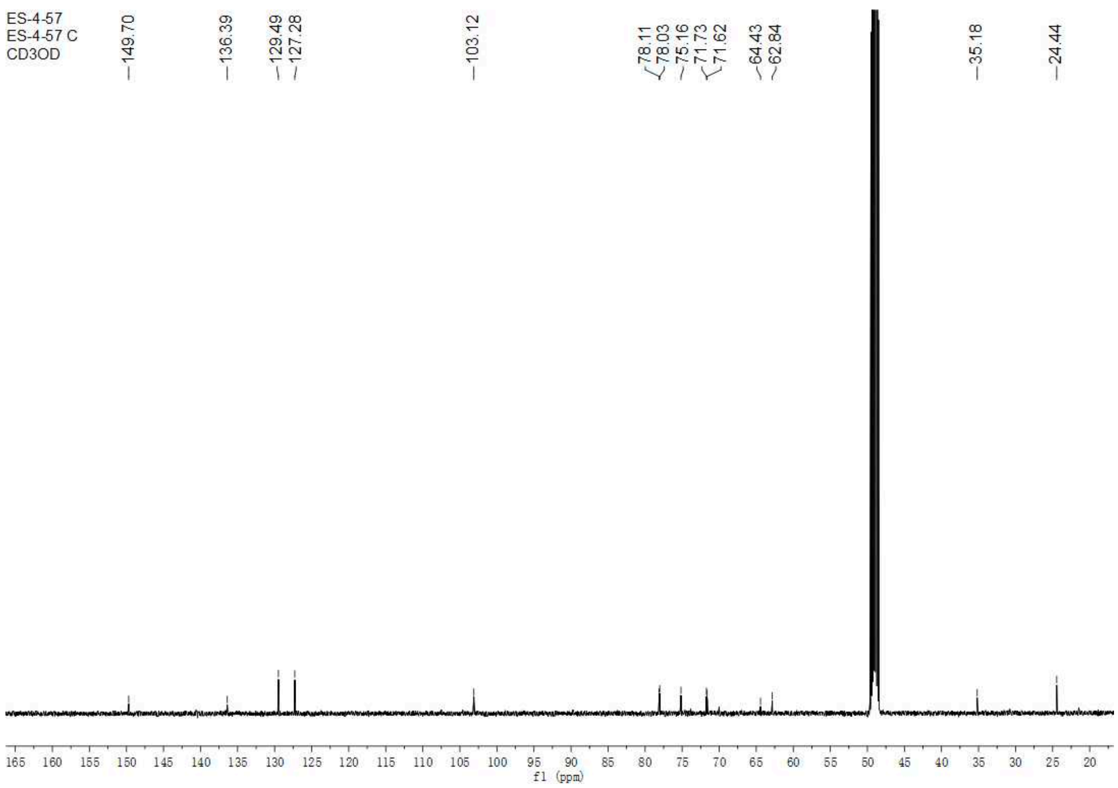

Figure S46. ^{13}C NMR spectrum (125MHz, CD_3OD) of **11**

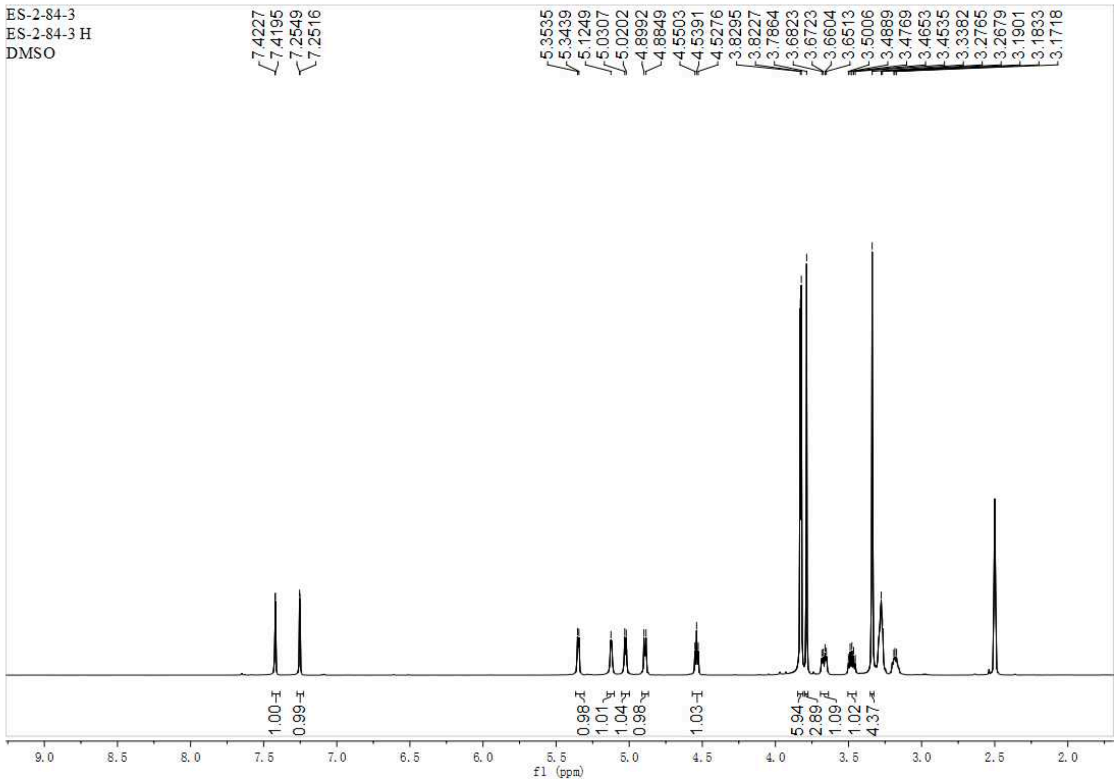

Figure S47. ^{1}H NMR spectrum (500MHz, CD_3OD) of **12**

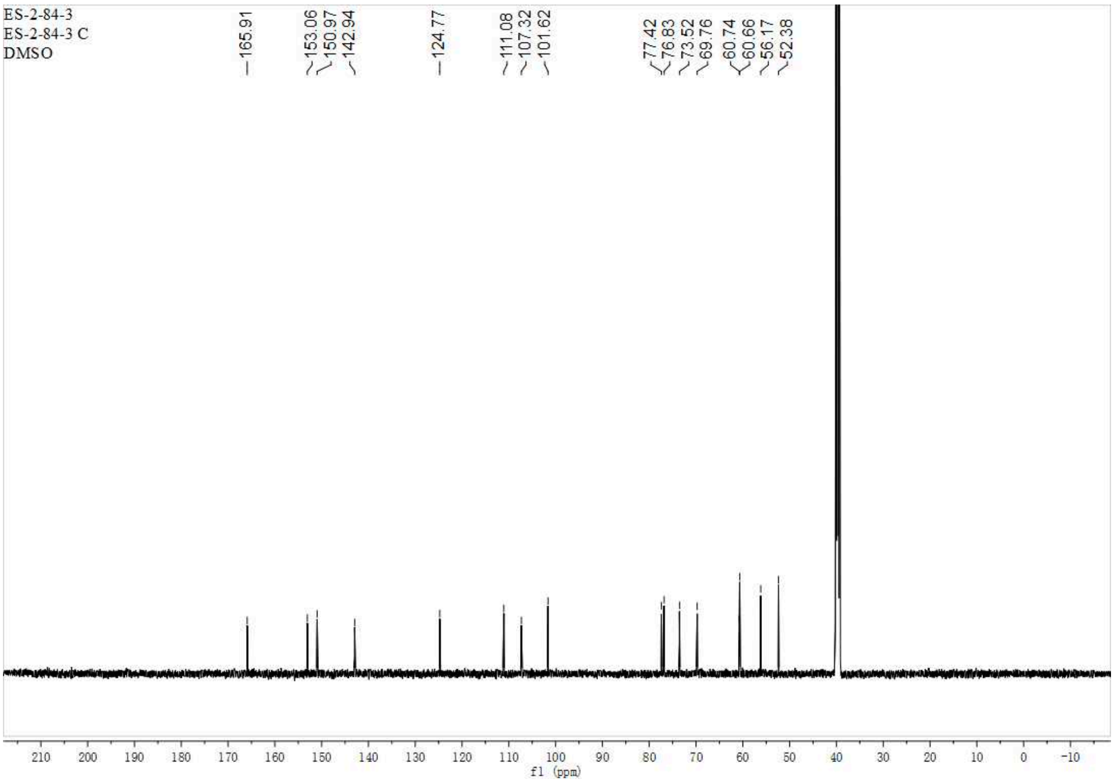

Figure S48. ^{13}C NMR spectrum (125MHz, CD_3OD) of **12**

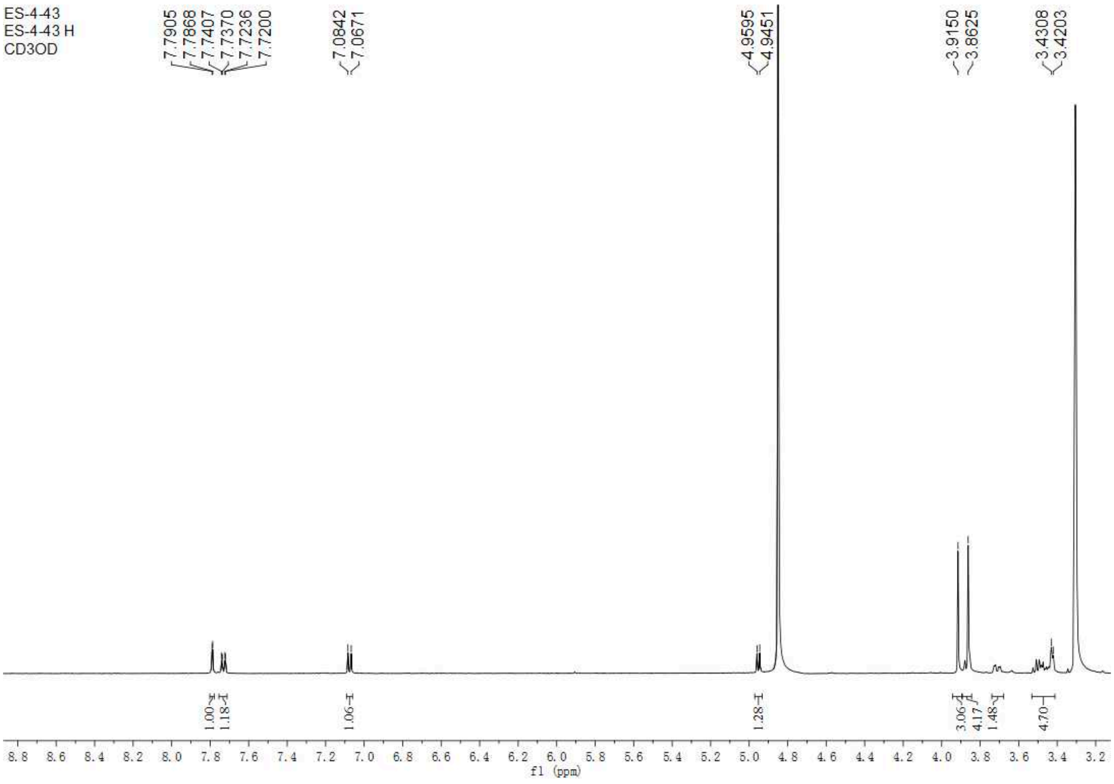

Figure S49. ^{1}H NMR spectrum (500MHz, CD_3OD) of **13**

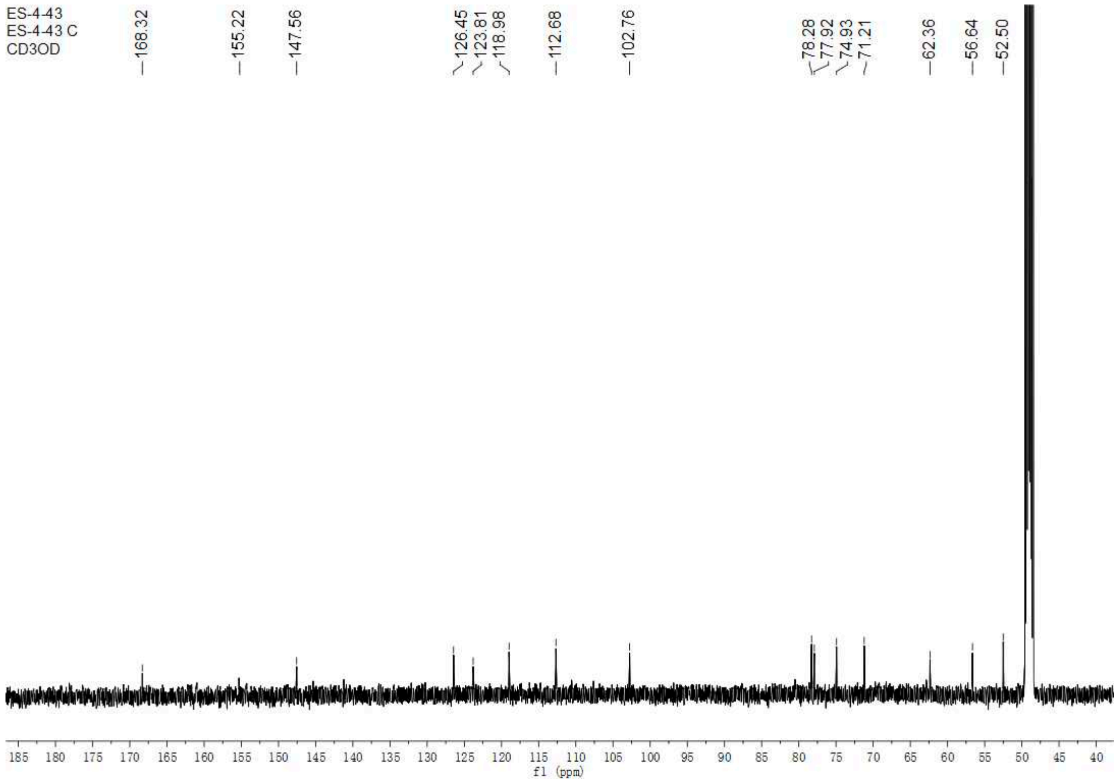

Figure S50. ^{13}C NMR spectrum (125MHz, CD_3OD) of **13**

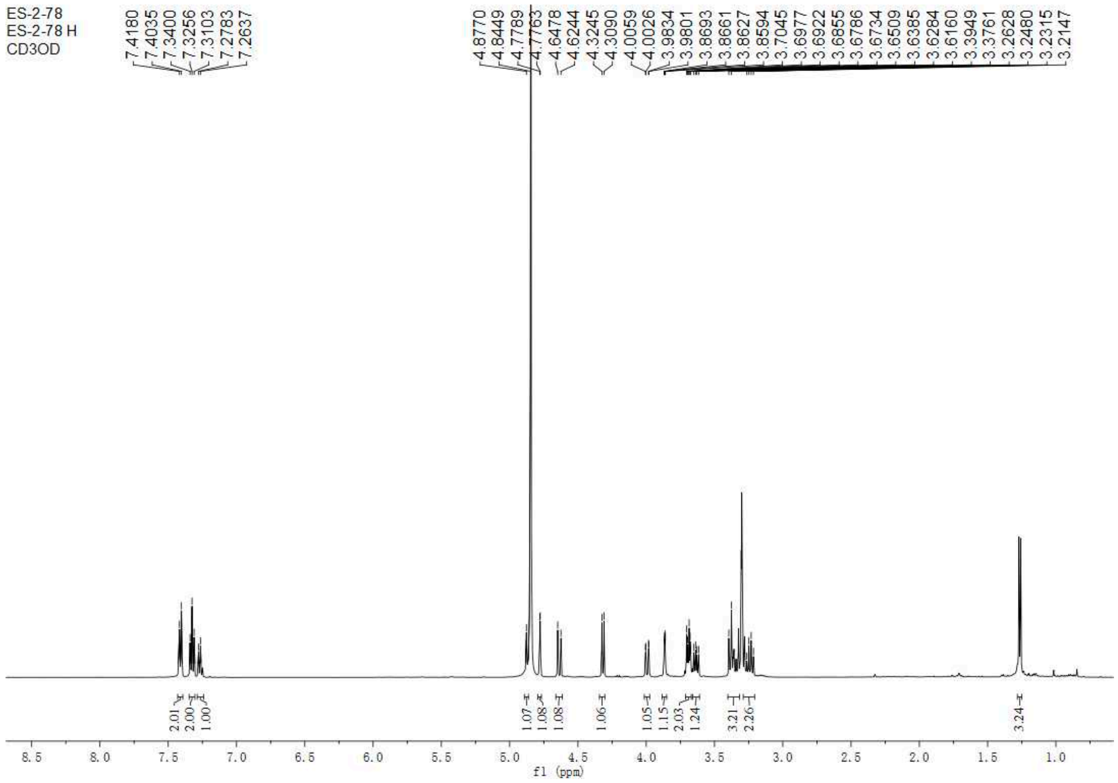

Figure S51. ^{1}H NMR spectrum (500MHz, CD_3OD) of **14**

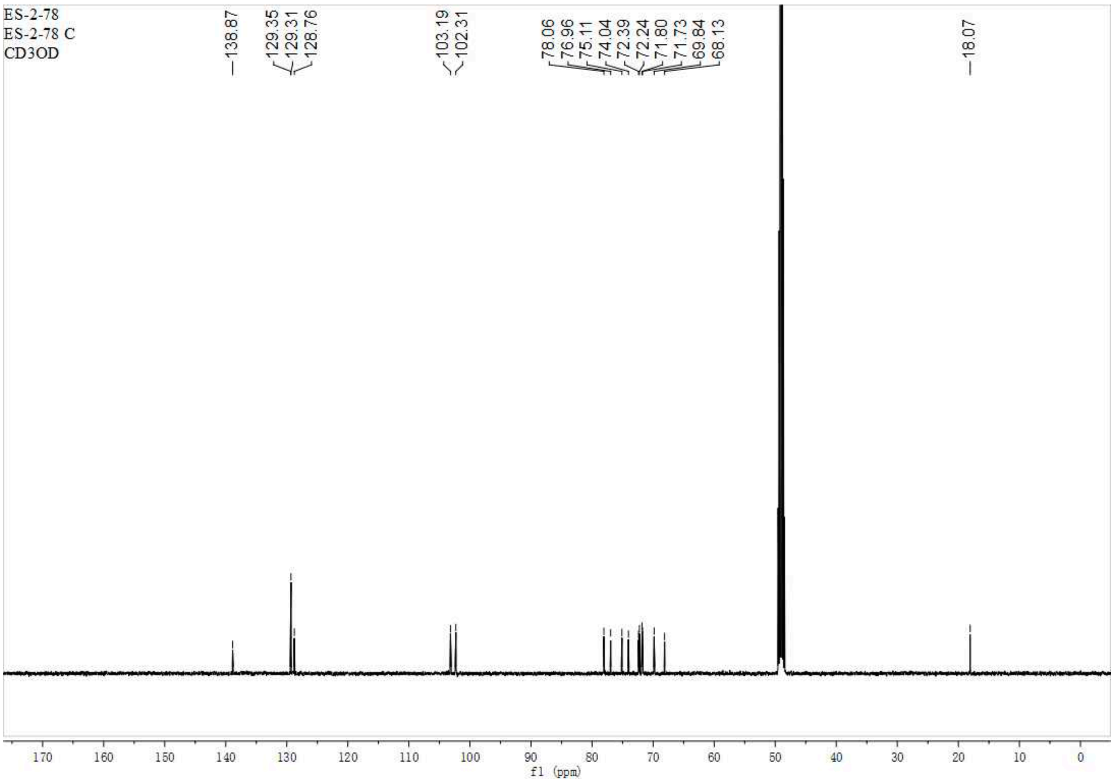

Figure S52. ^{13}C NMR spectrum (125MHz, CD_3OD) of **14**

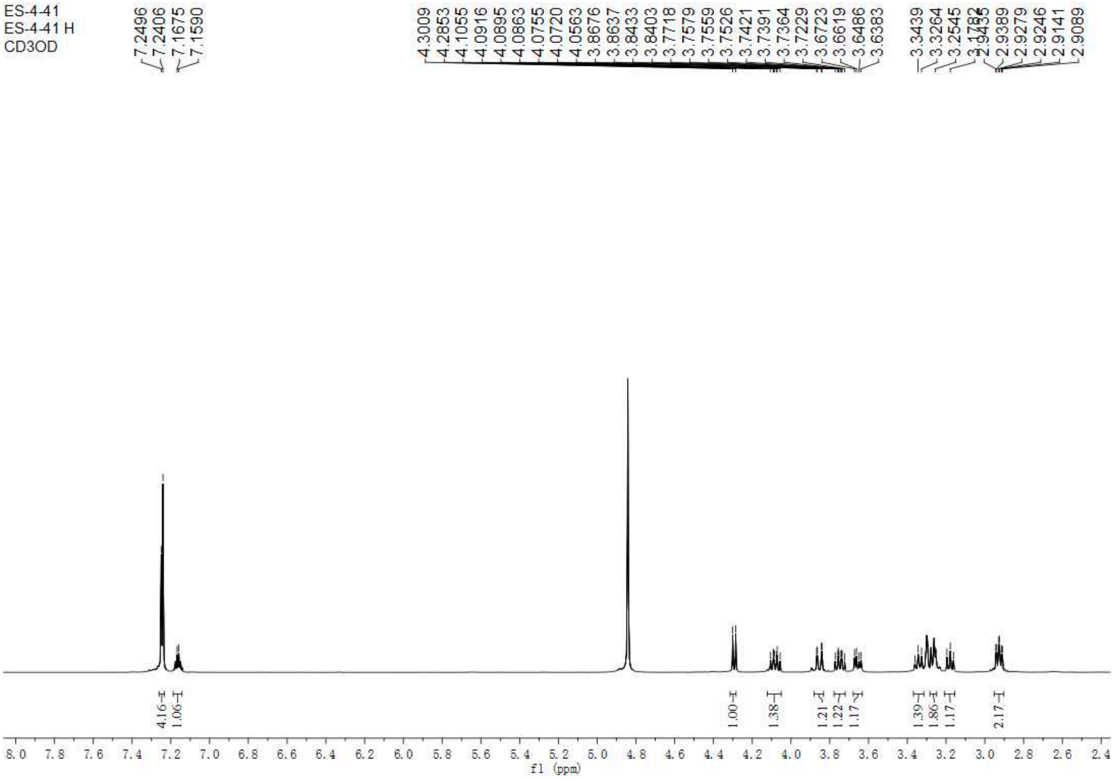

Figure S53. ^{1}H NMR spectrum (500MHz, CD_3OD) of **15**

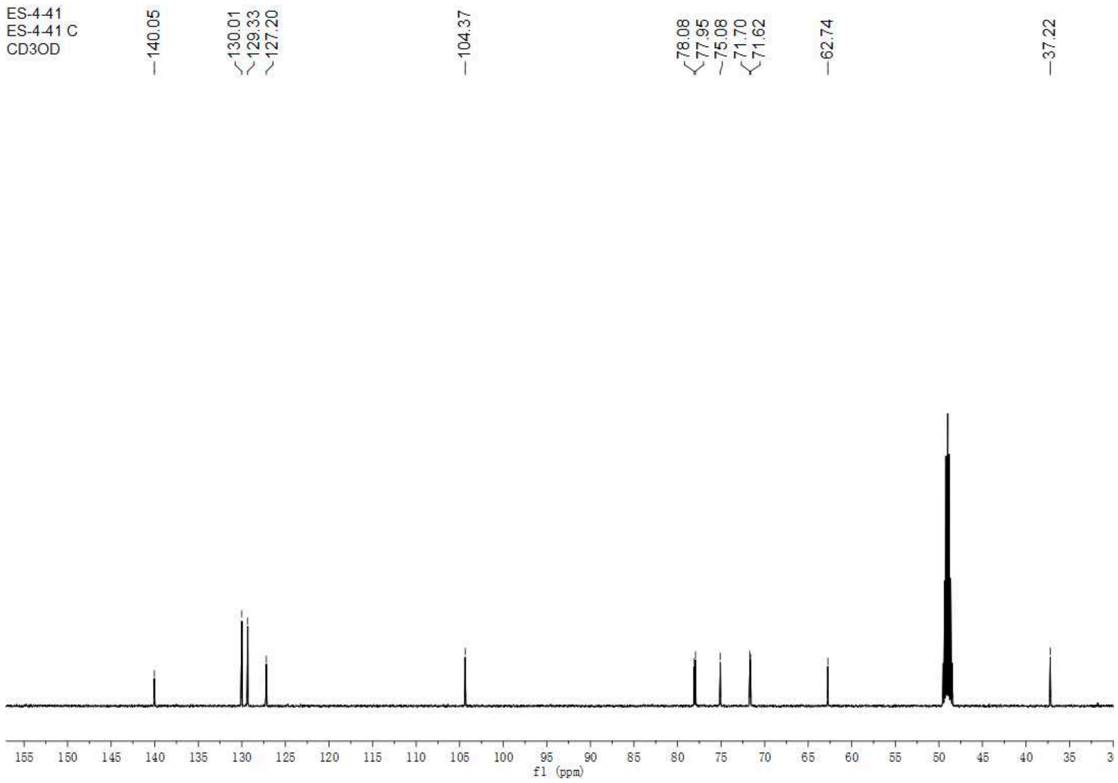

Figure S54. ^{13}C NMR spectrum (125MHz, CD_3OD) of **15**

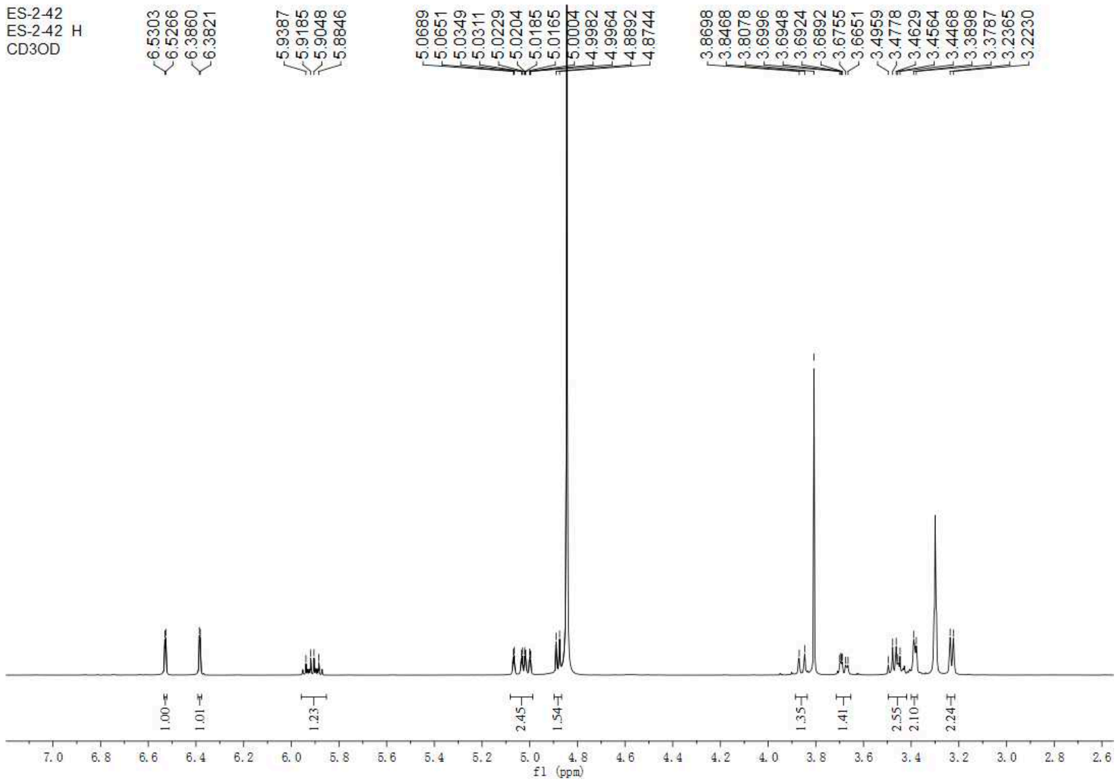

Figure S55. ^{1}H NMR spectrum (500MHz, CD_3OD) of **16**

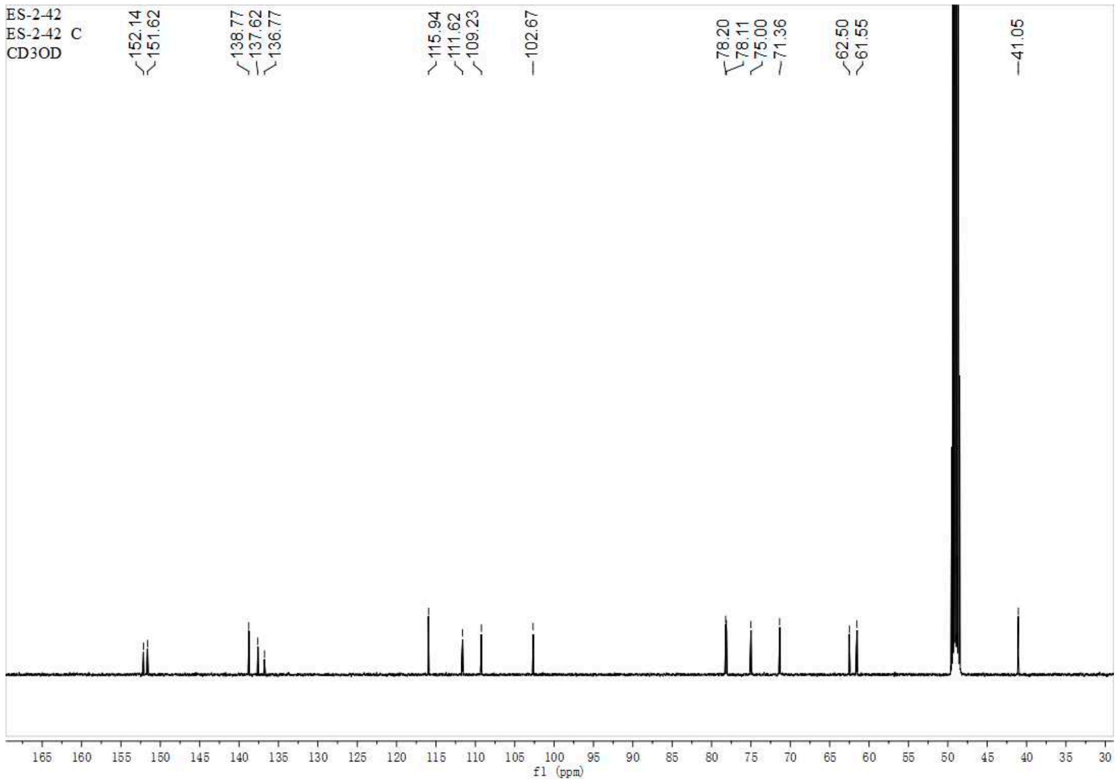

Figure S56. ^{13}C NMR spectrum (125MHz, CD_3OD) of **16**

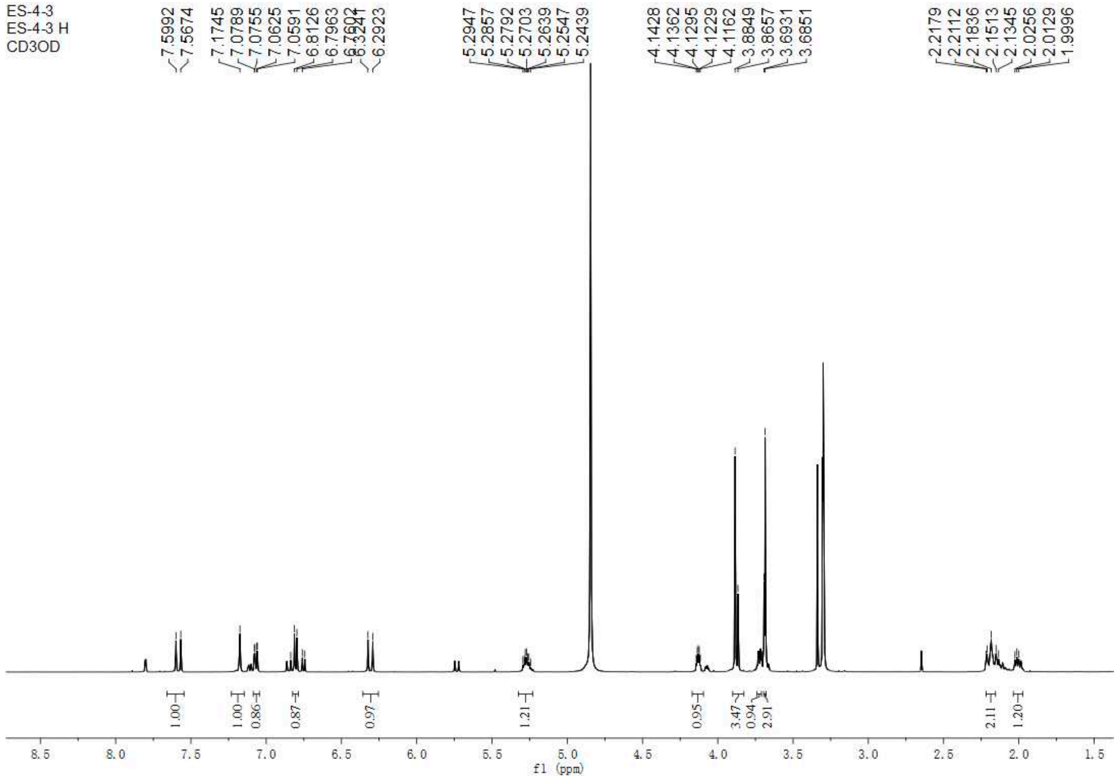

Figure S57. ^{1}H NMR spectrum (500MHz, CD_3OD) of **17**

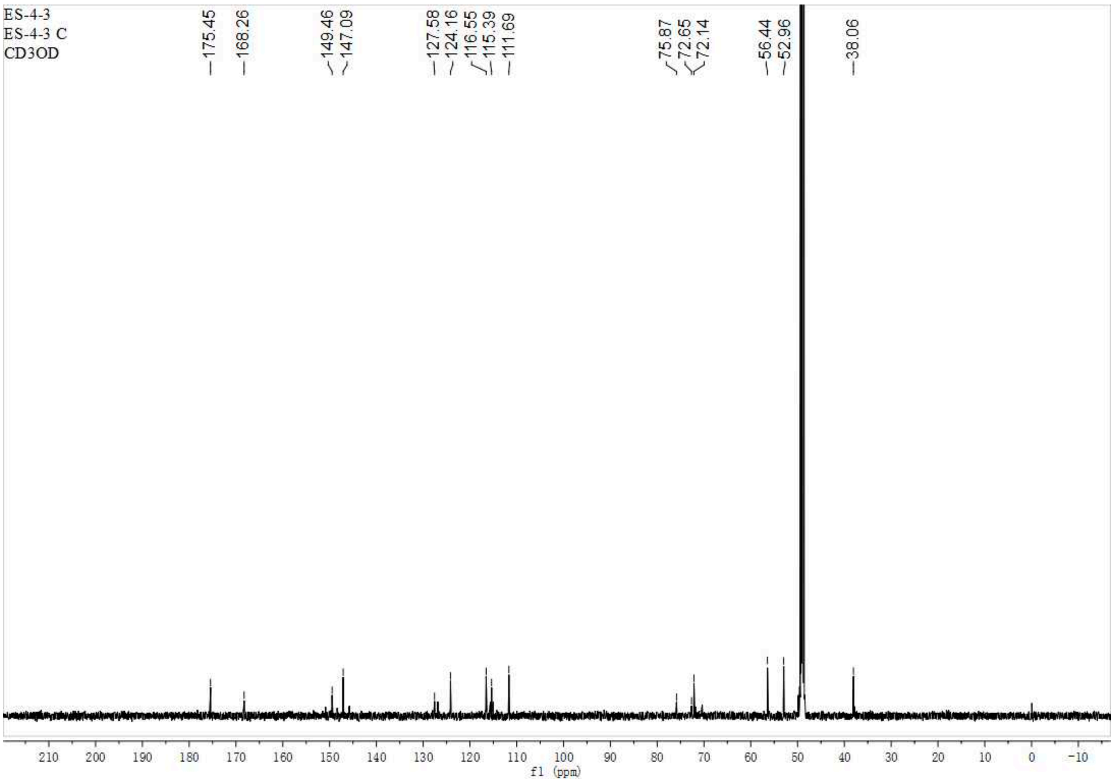

Figure S58. ^{13}C NMR spectrum (125MHz, CD_3OD) of **17**

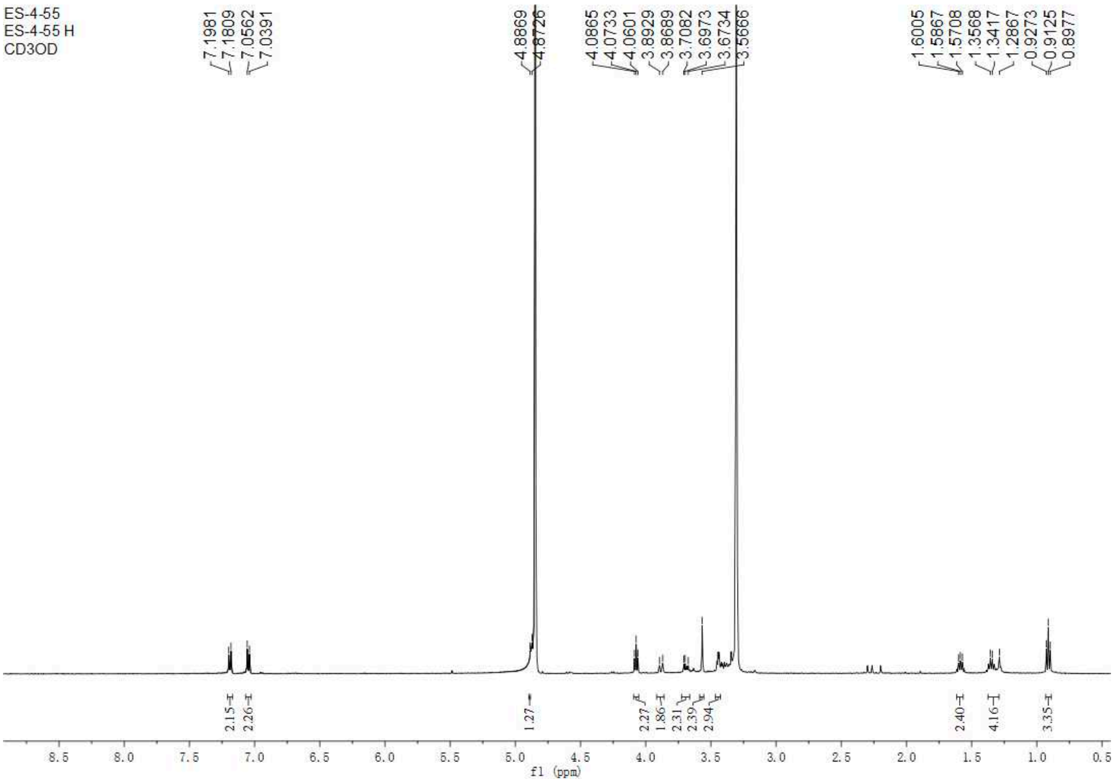

Figure S59. ^{1}H NMR spectrum (500MHz, CD_3OD) of **18**

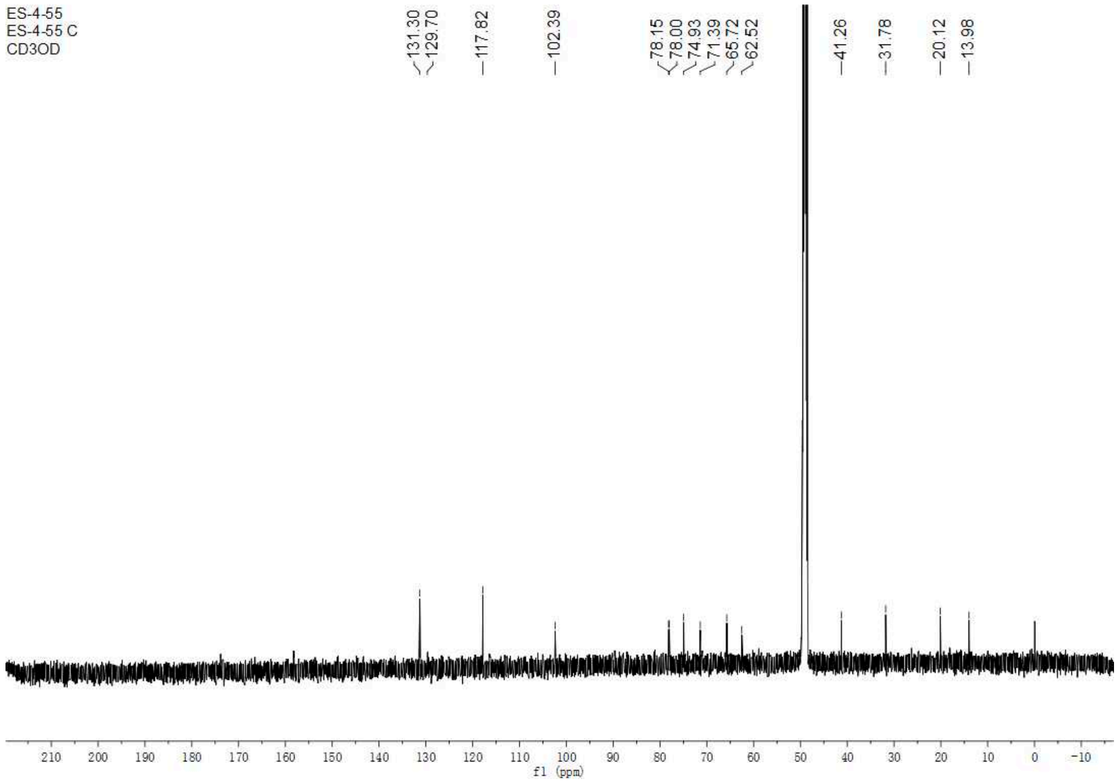

Figure S60. ^{13}C NMR spectrum (125MHz, CD_3OD) of **18**

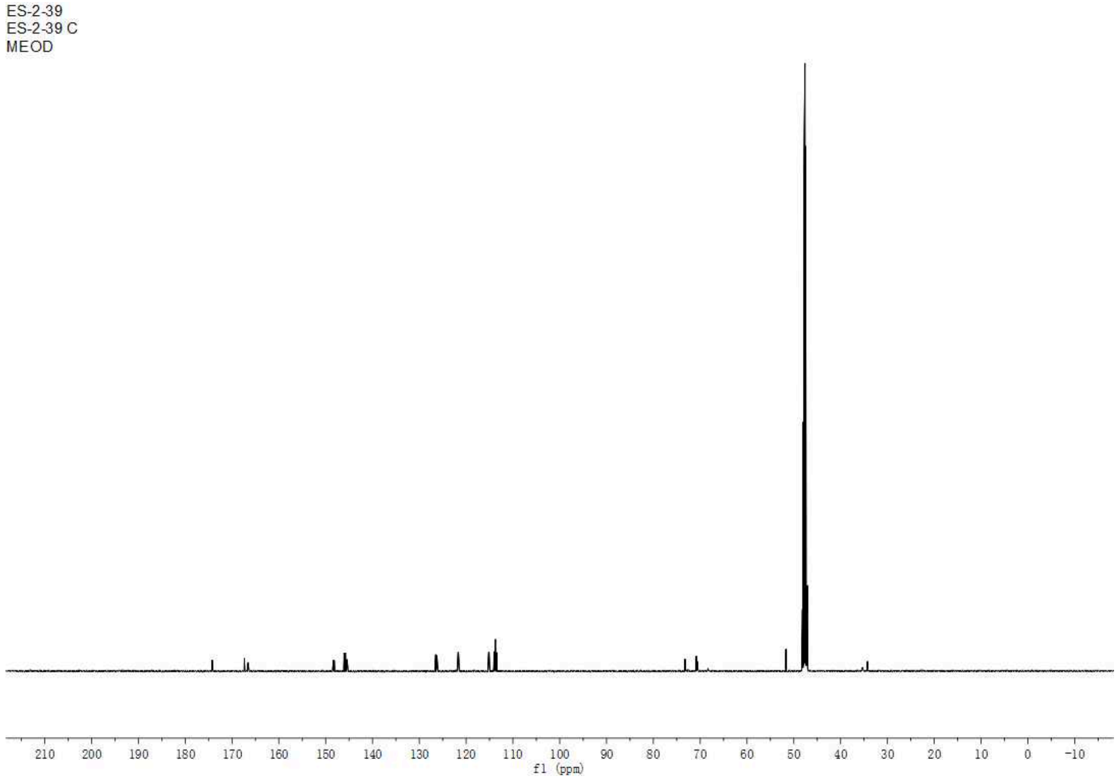

Figure S61. ^{1}H NMR spectrum (500MHz, CD_3OD) of **19**

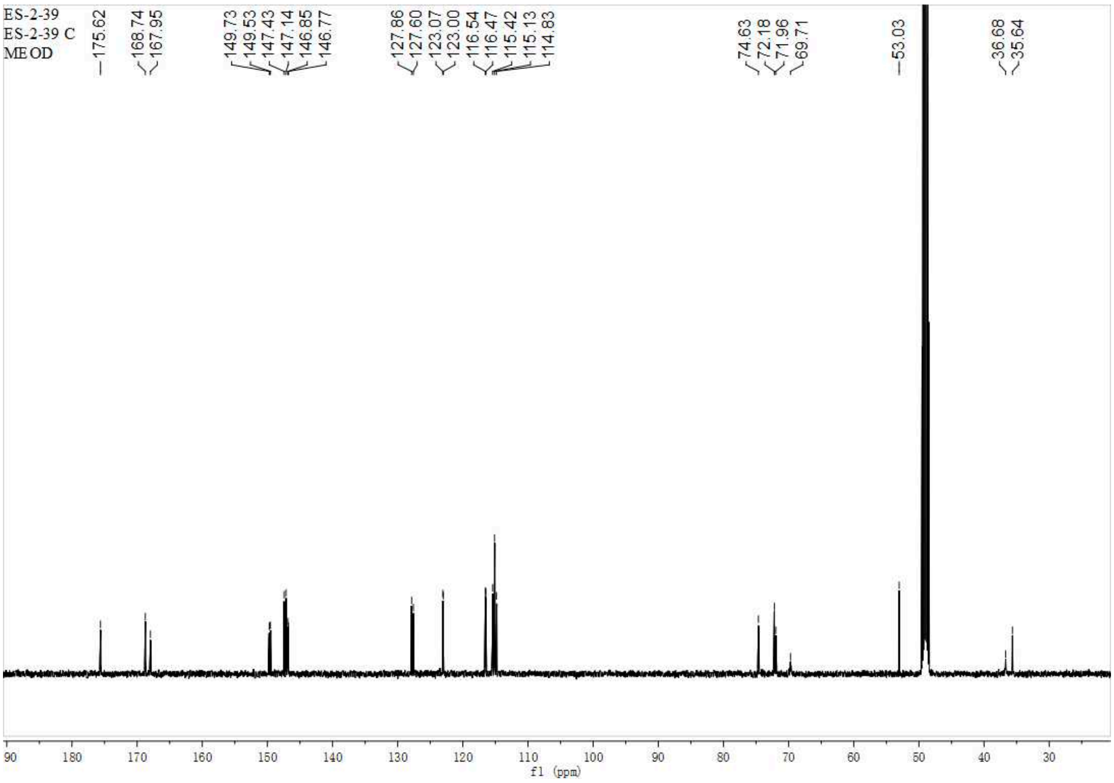

Figure S62. ^{13}C NMR spectrum (125MHz, CD_3OD) of **19** Compounds **8** and **9** (2.0 mg) were treated with 2.5 N aqueous HCl (2.0 mL) (sealed flask, 80 °C, 3 h). For compound **8** and **9**, the acidic aqueous mixture was dried, H_2O (2.5 mL) was added, and the mixture was extracted with EtOAc (4 × 2.5 mL). The dry aqueous layer was subjected to chiral-phase HPLC. The carbohydrate products of the hydrolysis of **8** and **9** were separated by a CHIRALPAK AD-H column (250 × 4.6 mm) using nhexane: EtOH:TFA (750:250:0.25) as the mobile phase (0.5 mL·min^{-1}) and detected by an evaporative light scattering detector (ELSD). By comparison with the retention time of standard substance, the sugar was identified as D-glucose (t_R = 18.3 min).

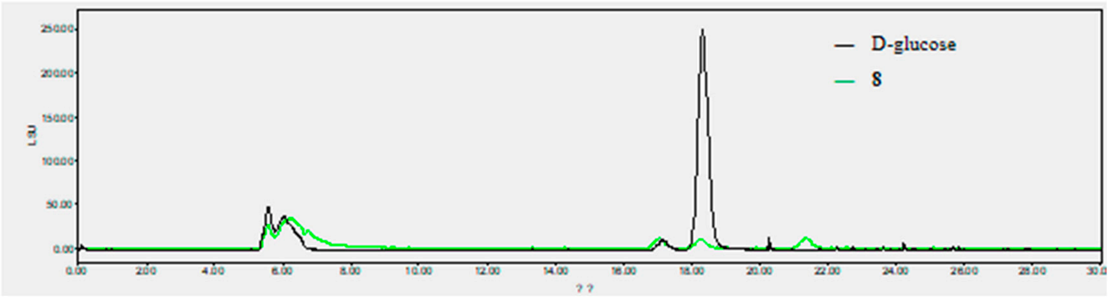

Figure S63. Chiral-HPLC profile from acid hydrolysis of **8** compared to authentic standard

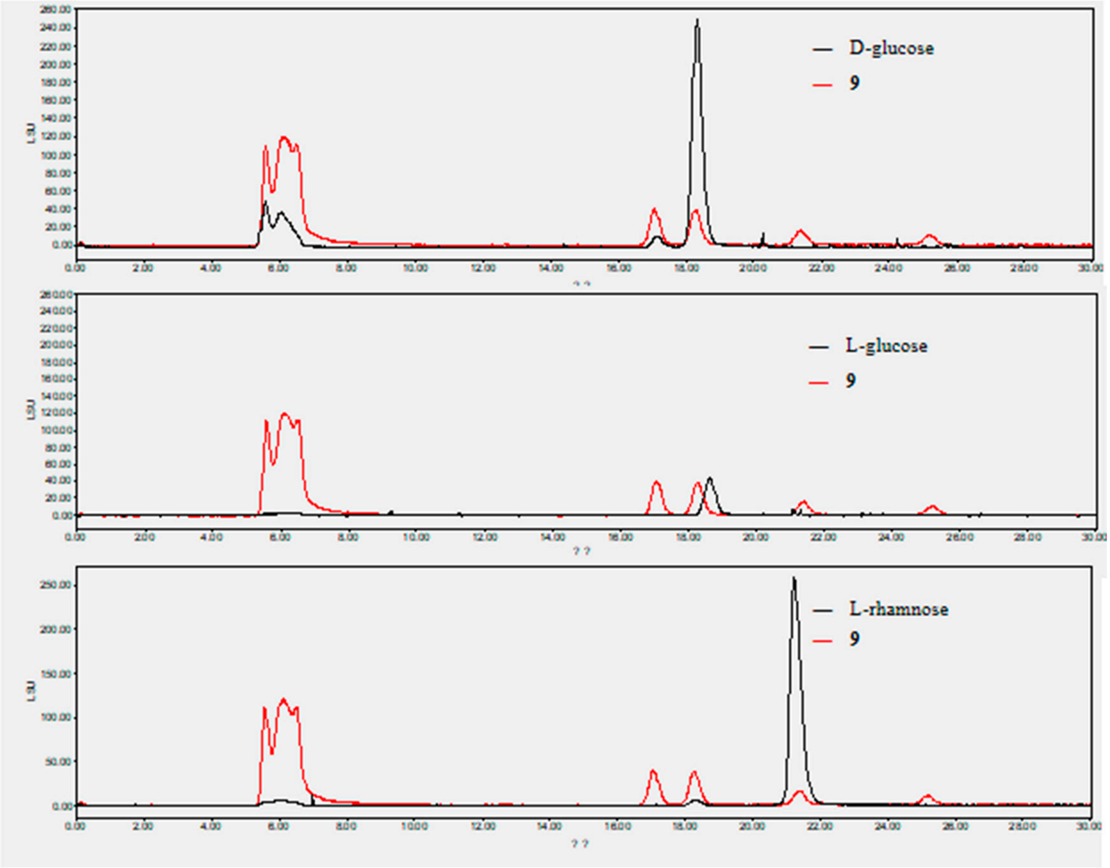

Figure S64. Chiral-HPLC profile from acid hydrolysis of **9** compared to authentic standard
